# Comparative Phylogeography Reveals Cryptic Diversity and Repeated Patterns of Cladogenesis for Amphibians and Reptiles in Northwestern Ecuador

**DOI:** 10.1371/journal.pone.0151746

**Published:** 2016-04-27

**Authors:** Alejandro Arteaga, R. Alexander Pyron, Nicolás Peñafiel, Paulina Romero-Barreto, Jaime Culebras, Lucas Bustamante, Mario H. Yánez-Muñoz, Juan M. Guayasamin

**Affiliations:** 1 Tropical Herping, Quito, Ecuador; 2 Centro de Investigación de la Biodiversidad y Cambio Climático (BioCamb), Ingeniería en Biodiversidad y Recursos Genéticos, Facultad de Ciencias de Medio Ambiente, Universidad Tecnológica Indoamérica, Quito, Ecuador; 3 Department of Biological Sciences, The George Washington University, Washington, D.C., United States of America; 4 Fundación EcoCiencia, Programa para la Conservación de Especies y Ecosistemas Amenazados en Ecuador, Quito, Ecuador; 5 División de Herpetología, Museo Ecuatoriano de Ciencias Naturales, Quito, Ecuador; Universita degli Studi di Roma La Sapienza, ITALY

## Abstract

Comparative phylogeography allow us to understand how shared historical circumstances have shaped the formation of lineages, by examining a broad spectrum of co-distributed populations of different taxa. However, these types of studies are scarce in the Neotropics, a region that is characterized by high diversity, complex geology, and poorly understood biogeography. Here, we investigate the diversification patterns of five lineages of amphibians and reptiles, co-distributed across the Choco and Andes ecoregions in northwestern Ecuador. Mitochondrial DNA and occurrence records were used to determine the degree of geographic genetic divergence within species. Our results highlight congruent patterns of parapatric speciation and common geographical barriers for distantly related taxa. These comparisons indicate similar biological and demographic characteristics for the included clades, and reveal the existence of two new species of *Pristimantis* previously subsumed under *P*. *walkeri*, which we describe herein. Our data supports the hypothesis that widely distributed Chocoan taxa may generally experience their greatest opportunities for isolation and parapatric speciation across thermal elevational gradients. Finally, our study provides critical information to predict which unstudied lineages may harbor cryptic diversity, and how geology and climate are likely to have shaped their evolutionary history.

## Introduction

Northwest Ecuador lies at the intersection of two of the most diverse terrestrial ecoregions on the planet, the Andes and the Choco. Together, these harbor ~18.5% of the total diversity of terrestrial vertebrates [1, 2, 3]. For example, in Mindo, Ecuador, a transitional valley of only 268 km^2^ located at the Choco/Andes transition ~1000m ASL, 101 species of amphibians and reptiles have been registered [4]. Another locality in NW Ecuador, Bilsa Biological Station, harbors 109 species of herpetofauna in only 33 km^2^ [5]. In comparison, richer herpetofaunal communities in the Neotropics are found only in the upper Amazon basin [6], with 220 in Leticia, Colombia [7], 185 in Santa Cecilia, Ecuador [8], and 271 in Yasuni National Park, Ecuador [9].

Diversity in this transitional area could be explained by a history of biotic interchanges characterized by greater immigration than emigration of lineages [10, 11, 12], lower rates of extinction [12, 13], or greater rates of speciation than other regions [12, 13]. The latter could be explained by a synergic effect between: i) the geographic and climatic complexity of tropical mountainous areas, which promotes vicariance-based speciation during uplift and through dispersal-based speciation following orogenesis [14, 15]; ii) the evolutionary conservatism of climatic niches, which limits elevational dispersal [16, 17]; and iii) the time that lineages have persisted in the region, which increases the lineages’ opportunities to disperse and differentiate across geographical barriers [13, 17, 18].

Some evidence (see below) points to faster speciation in the complex watersheds and montane ecosystems of the Andes and Choco, through simple models of climate-induced vicariance [17, 19, 20, 21]. Several authors [4, 22, 23, 24, 25] have already suggested that both the valleys and the large river systems of this region have effectively limited dispersion among populations. However, no studies determined if these elements of the landscape have affected distantly related lineages of herpetofauna in the same way. Barriers and ecological gradients might be common to all lineages, but ultimately what determines the pattern of speciation in an area is the commonality by which those barriers affect dispersal across taxa [26, 27, 28]. Evidence for allopatric speciation driven by geographical barriers is abundant [29], but evidence for parapatric speciation along ecological gradients remains scarce [30, 31], although this latter pattern is suggested to have played an important role in the speciation of amphibians in the Andes [22].

One way to study the effect that geographical barriers have on the diversification of distinct groups of organisms is comparative phylogeography [32, 33, 34]. These studies at the molecular and geographical level make it possible to: i) infer patterns of species diversification from the current geographic distribution of genetic diversity [35, 36], ii) evaluate the impact of historical events on the genetic composition and structure of biotic assemblages [37, 38, 39], and iii) unveil cryptic lineage diversity that may be common to other co-distributed, but as yet unstudied, groups.

Recent studies in Ecuador addressing geographic patterns of diversification have been focused on groups of closely related amphibians [10, 17] and reptiles [40, 41]. None of these studies have used a comparative phylogeographic approach across reptiles and amphibians. Studies from other regions [27, 35] that contain a diverse sample of taxonomic and ecological groupings have been able to answer different questions and provide a wider perspective of the co-diversification and speciation of their target area. In this study, we use two sister-species pairs of reptiles belonging to the families Gymnopthalmidae and Viperidae; and three of amphibians belonging to the family Craugastoridae, to describe geographic patterns of diversification. The sister-species pairs were chosen for i) being co-distributed in northwestern Ecuador and ii) having been considered conspecific in the past.

Across frogs, lizards, and snakes, we find a generalized pattern of geographic displacement of sister species across elevational gradients, with a widely distributed Chocoan lineage replaced by a localized Andean cloudforest lineage. This suggests that climate-mediated diversification along elevational gradients is a primary driver of herpetological diversity in the northern Andes. This mirrors results from other taxa, such as butterflies [42], glassfrogs [17], and birds [43].

## Materials and Methods

### Ethics statement

This study was carried out in strict accordance with the guidelines for use of live amphibians and reptiles in field research compiled by the American Society of Ichthyologists and Herpetologists (ASIH), The Herpetologists' League (HL) and the Society for the Study of Amphibians and Reptiles (SSAR). All procedures with animals (see below) were approved by the Centro de Investigación de la Biodiversidad y Cambio Climático (BioCamb) of the Universidad Tecnológica Indoamérica. They also were reviewed by the Ministerio de Ambiente del Ecuador (MAE) and specifically approved as part of obtaining the following field permits for research and collection: N°14-2011-IC-FAU-DPAP-MA, N°05-2013-IC-FAU-DPAP-MA, N°01-2014-AD-RIC-FAU-DPAP-MA and MAE-DNB-CM-2015-0017, granted to Juan M. Guayasamin through Universidad Tecnológica Indoamérica; permit N°012-IC-FAN-DPEO-MAE, granted to Mario Yánez-Muñoz through the Museo Ecuatoriano de Ciencias Naturales; and permit N°005–15 IC-FAU-DNB/MA, granted to Luis A. Coloma through the Centro Jambatu de Investigación y Conservación de Anfibios. Specimens were euthanized with 20% benzocaine, fixed in 10% formalin or 70% ethanol, and stored in 70% ethanol. Museum vouchers were deposited at the Museo de Zoología of the Universidad Tecnológica Indoamérica (MZUTI).

### Sampling

**Fig 1:**
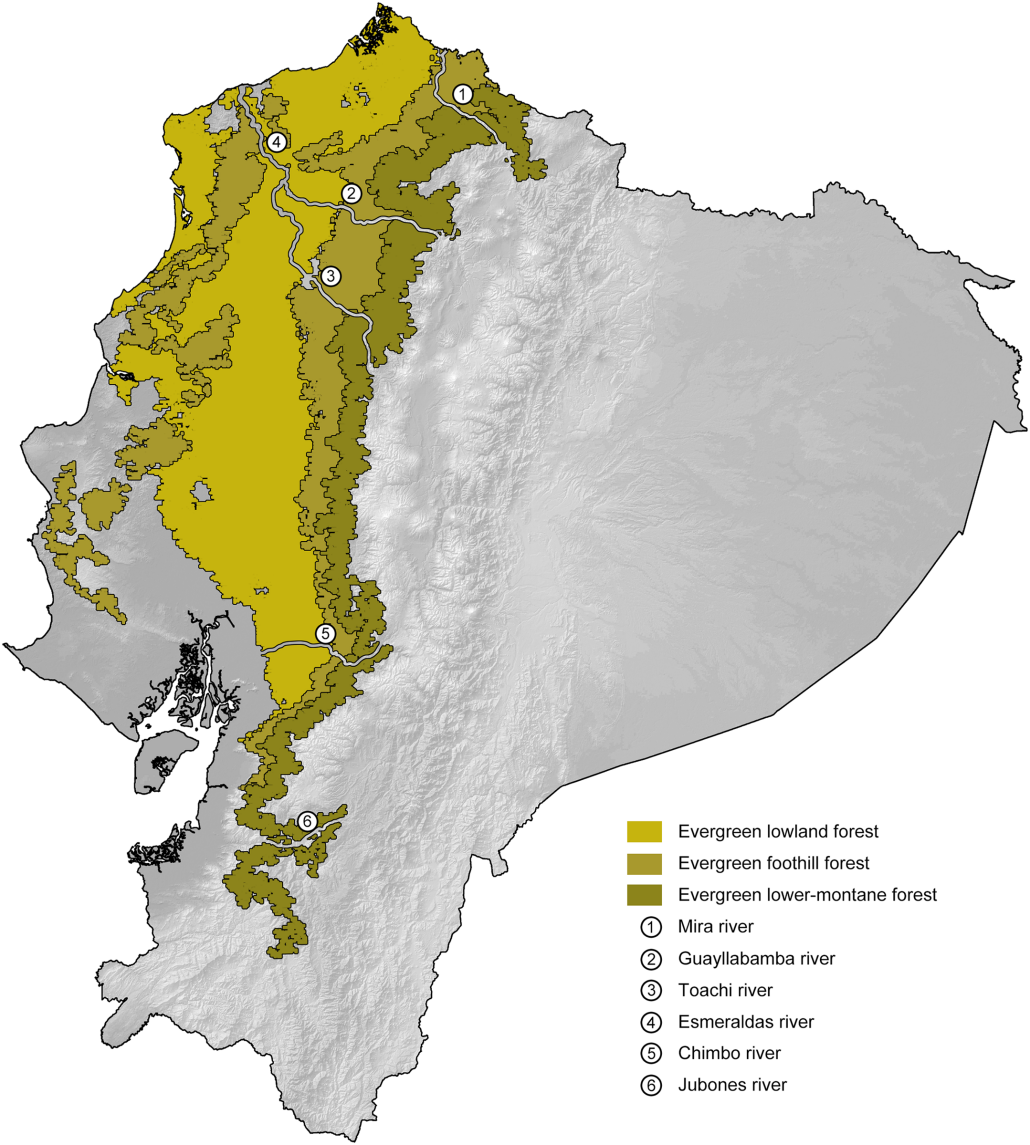
Main vegetation zones and rivers in the Ecuadorian northwest. The map is a simplified version of the main vegetation zones of Sierra [55].

Tissue samples from 112 individuals representing 15 species (including two new species described here) were obtained from 24 localities throughout their distributions in western Ecuador (Table 1) (Fig 1). At each locality, sampling sites were chosen to coincide with previously established trails, or along bodies of water or roads. In these sites, groups of 2–8 people carried out visual encounter surveys [44] from 20h00 to 02h00 for no less than three consecutive nights. The majority of individuals were located by space-constrained visual examination of vegetation and ground-level substrates [45]. The remaining individuals were detected by turning over logs, rocks and other surface objects. No more than four specimens per species per locality were collected by us, and in some cases, individuals were released after sampling (Vouchers AA and ANF under S1 Table). Our study focuses on five lineages. Each lineage contains two species known to be the closest morphological relative of each other based on most recent works [4, 11, 23, 46, 47, 48, 49, 50, 51]. Based on these topologies, we also chose the species to be used as outgroups in phylogenetic analyses. The five lineages share similar patterns of distribution, but have dispersal characteristics and life history traits that range from habitat generalists capable of long-distance migrations in *Bothrops* [52, 53, 54], to habitat specialists with narrow capacity of dispersal in *Pristimantis* [22].

**Table 1:** Localities in Ecuador sampled during this study.

Province	Locality	Latitude	Longitude	Elev.
Azuay	Flor y Selva	-2.65706	-79.53111	136
Cañar	Huatacón	-2.49018	-79.18223	1048
El Oro	Buenaventura	-3.66598	-79.73933	1042
El Oro	California	-3.37146	-79.73430	328
Esmeraldas	Bilsa	0.34910	-79.70967	555
Esmeraldas	Canandé	0.52645	-79.20937	360
Esmeraldas	Itapoa	0.51306	-79.13396	341
Esmeraldas	Mache Chindul	0.51032	-79.72552	175
Esmeraldas	Tundaloma	1.18317	-78.75245	74
Imbabura	Los Cedros	0.31842	-78.78373	1764
Pichincha	Cascadas de Mindo	-0.07837	-78.76429	1438
Pichincha	Chontilla	0.11187	-78.90275	1191
Pichincha	El Abrazo	-0.00916	-78.81133	1086
Pichincha	Las Gralarias	-0.00158	-78.73858	1793
Pichincha	Mashpi lodge	0.16352	-78.87274	1060
Pichincha	Milpe	0.03489	-78.86713	1070
Pichincha	Sachatamia	-0.02470	-78.75909	1704
Pichincha	Selva Virgen	0.10673	-78.18542	355
Pichincha	Séptimo Paraíso	-0.02808	-78.76667	1537
Pichincha	Silanche	0.14577	-79.14338	418
Pichincha	Sueños de Bambú	-0.06655	-78.77158	1391
Pichincha	Tandayapa Lodge	0.00249	-78.68083	1730
Pichincha	Yellow House	-0.04505	-78.75938	1498
Santo Domingo	Otongachi	-0.32145	-78.95094	661

All specimens included in the genetic analyses were morphologically identified according to Lynch and Duellman [22], Arteaga *et al*. [4], Campbell and Lamar [56] and Torres-Carvajal and Lobos [23]. We generated sequence data for samples marked with an asterisk under S1 Table, which includes museum vouchers at the Museo de Zoología of the Universidad Tecnológica Indoamérica (MZUTI) and the División de Herpetología del Museo Ecuatoriano de Ciencias Naturales (MECN), along with individuals released after sampling (AA, ANF).

### Laboratory techniques

Genomic DNA was extracted from 96% ethanol-preserved tissue samples (liver, muscle tissue or scales) using a modified salt precipitation method based on the Puregene DNA purification kit (Gentra Systems). For amphibians, we amplified the mitochondrial 12S gene using the primers t-Phe-frog and t-Val-frog developed by Wiens *et al*. [57]. When low-quality product or no product at all was retrieved, 12S primers for reptiles were used (see below). In the same way, for the 16S gene we initially tried the primers 16Sc-F and 16Sbr-H-R developed by Darst and Cannatella [58] and Palumbi *et al*. [59], respectively. If the amplification was unsuccessful or unsatisfactory, the 16S primers for reptiles described ahead were used. For reptiles, we amplified the 12S gene using the primers Snake_12S_F, a shortened version of L1091 from Kocher *et al*. [60], and Snake_12S_R developed as 12e by Wiens *et al*. [61] and the 16S gene using the primers Snake_16S_F, which consists in a slight modification of 16Sar-L from Palumbi *et al*. [59] and Snake_16S_R, which is exactly the same as 16Sbr-H-R. Additionally, the Cytb gene was obtained with the primers Snake_Cytb_F and Snake_Cytb_R developed as L14910 and H16064 respectively by Burbrink *et al*. [62], whereas the subunit 4 of the NADH dehydrogenase mitochondrial gene was amplified with primers Snake_ND4_F and Snake_ND4_R developed as ND4 and Leu, respectively, by Arévalo *et al*. [63]. The nucleotide sequences of the primers and the PCR conditions applied to each primer pair are detailed in S2 Table. PCR reactions were set up to a total volume of 25 μL containing 1μL of total DNA, 3 mM MgCl_2_, 200 μM each dNTP, 0.2 μM each primer, and 1.25 units of DNA *Taq* polymerase (Invitrogen) with the accompanying PCR buffer at 1X final concentration. These conditions applied for all primer pairs, except for those for ND4 and 12S (reptiles) genes. In these cases, final concentration of 2 mM MgCl_2_, 0.8 μM each primer and 0.625 of *Taq* polymerase were used. PCR products were visualized in 1.5% agarose gel, and unincorporated primers and dNTPs were removed from PCR products by ExoI/SAP digestion. Cycle sequencing reactions were performed by Macrogen Labs (Macrogen Inc., Korea). All fragments were sequenced in both forward and reverse directions with the same primers that were used for amplification. The sequences were deposited in GenBank (S1 Table).

### DNA sequence analyses

A total of 357 mtDNA sequences (212 generated during this work and 145 downloaded from GenBank) were incorporated in the analyses (S1 Table). From these, we used 129 sequences to build a mitochondrial phylogenetic tree of the genus *Bothrops*; 50 for the genus *Alopoglossus*; 47 for the *Pristimantis* (*Hypodictyon*) *rubicundus* species series [51] [64]; 53 for the *Pristimantis lacrimosus* species group [51, 65], which includes *P*. *subsigillatus* and *P*. *mindo* [4]; and 78 to build a mitochondrial phylogenetic tree of the Ecuadorian yellow-groined rainfrogs of the *Pristimantis unistrigatus* species group [22, 51]. Based on the topology recovered in previous studies [11, 48, 49, 51], we decided to include three members of the *Pristimantis* (*Hypodiction*) *ridens* series [51] as outgroups, along with novel sequences for *P*. *luteolateralis*, *P*. *parvillus*, *P*. *walkeri*, and two other species previously subsumed under *P*. *walkeri*.

Novel sequences were edited and assembled using the program Geneious ProTM 5.4.7 [66], and aligned with those downloaded from Genbank (S1 Table) using MAFFT v.7 [67] under the default parameters in Geneious ProTM 5.4.7. For snakes, genes were combined into a single matrix with eight partitions, one per non-coding gene and three per protein coding gene corresponding to each codon position. For amphibians, genes were combined into a single matrix with two partitions, one per each gene. The best partition strategies along with the best-fit models of evolution were obtained in PartitionFinder 1.1.1 [68] and jModeltest [69] under the Bayesian information criterion. Phylogenetic relationships were assessed under a Bayesian approach in MrBayes 3.2.0 [70]. Four independent analyses were performed to reduce the chance of converging on a local optimum. Each analysis consisted of 6.7 million generations and four Markov chains with default heating settings. GenBank accession numbers are listed in S1 Table. Trees were sampled every 1,000 generations, resulting in 5,000 saved trees per analysis after 25% of those were arbitrarily discarded as ‘‘burn-in.” Stationarity was confirmed by plotting the–ln L per generation in the program Tracer 1.2 [71]. Genetic distances were calculated using the uncorrected distance matrix in PAUP 4.0 [72].

### Morphological data

Generic and family names used in this study follow Pyron and Wiens [48] for amphibians, Hendry *et al*. [46] for vipers and Pellegrino *et al*. [73] for lizards. To examine species boundaries within *Pristimantis*, our diagnoses and descriptions generally follow Duellman and Lehr [74]. We examined comparative alcohol-preserved specimens from the herpetology collections at the MZUTI, MECN and Fundación Herpetológica Gustavo Orcés (FHGO) (S3 Table). When providing the standard deviation, we use the ± symbol. Morphological measurements were taken with digital calipers to the nearest 0.1 mm, as described by Lehr and Coloma [75]. These are as follows: (1) snout–vent length (SVL), (2) tibia length, (3) foot length, (4) head length, (5) head width, (6) eye diameter, (7) interorbital distance, (8) upper eyelid width, (9) internarial distance, (10) eye–nostril distance. Sexual maturity was determined by the presence of testis or vocal slits in males and by the presence of eggs or convoluted oviducts in females.

### Nomenclatural acts

The electronic edition of this article conforms to the requirements of the amended International Code of Zoological Nomenclature, and hence the new names contained herein are available under that Code from the electronic edition of this article. This published work and the nomenclatural acts it contains have been registered in ZooBank, the online registration system for the ICZN. The ZooBank LSIDs (Life Science Identifiers) can be resolved and the associated information viewed through any standard web browser by appending the LSID to the prefix "http://zoobank.org/". The LSID for this publication is: urn:lsid:zoobank.org:pub: 1192CFA6-7964-441E-BE22-6DC932A748E0. The electronic edition of this work was published in a journal with an ISSN, and has been archived and is available from the following digital repositories: PubMed Central, LOCKSS.

### Distribution maps

We present ranges of occurrence graphically in the form of spatially distributed dots on a colored representation of Ecuador's relief. Each dot indicates a locality where the species has been observed. This includes published records, photographic vouchers (Tropical Hering database), HerpNET data for reptiles, and museum specimens deposited at MZUTI, MECN and FHGO (S3 Table). For all species in the study, a binary environmental niche model (ENM) accompanies the dot maps, whereas each corresponding non-binary environmental niche model is presented under S1 Fig. ENMs for sister species are included on the same map to show their degree of overlapping. These models estimate potential areas of distribution, on the basis of observed presences and a set of environmental predictors [76]. To delimit the ocupancy areas and the potencial species distribution, we used the BAM diagram proposal [77, 78]. To create the models, we used presence localities listed under S4 Table, 19 bioclimatic variables from Worldclim 1.4 [79] and Maxent 3.3.3k, an algorithm based on the principle of maximum entropy [80, 81, 82]. The analysis is detailed below:

For the first explorative exercise, we used the 19 climate layers from the WorldClim project and assessed which variables were the most important for the model, according to the Jackniffe test calculated in MaxEnt [83]. Correlated environmental variables (r < 0.8) were identified using the PEARSON correlation test of PAST 3. In a second modelling exercise, we used the locality records for each species (S4 Table) and the variables identified in the first approach to generate the species distribution.

The overall predictive models of distribution were generated with 80% of the locality records (training data) and the other 20% were used for evaluation (testing data). In addition, 5,000 iterations were specified to the program with clamping and no extrapolation. All other parameters in MaxEnt were maintained at default settings.

To create the binary environmental niche models, suitable areas were distinguished from unsuitable areas by setting a *minimum training presence* threshold value. The logistic format was used to obtain the values for habitat suitability (continuous probability from 0 to 1), which were subsequently converted to binary presence-absence values on the basis of the established threshold value, defined herein as the *minimum training presence*.

The convergence threshold was set to 10^−5^, maximum iterations to 500, and the regularization parameter to “auto.” Finally, to assess the performance of the model, we used the area under the curve (AUC), a metric that compares model outputs with null expectations using a threshold-independent measure [84]. A value of 0.5 indicates that the model is no better than random, and AUC = 1 indicates that the model discriminates perfectly between presence and absence [85].

The percentage overlap of each species’ ENM with the main biogeographic regions of western Ecuador was calculated using the raster calculator tool of ArcGIS 10. The layers for the ecological niche models and those for the biogeographic have 1 km^2^ pixels that represent absence (0) and presence (1). When applied, the raster calculator tools adds up the values of presence each pixel, and these can be extracted to a table of contents to obtain the total numbers of pixel with a combined value (overlap = 2).

Based on museum specimens and literature records [4, 22, 23, 86, 87, 88, 89, 90, 91, 92, 93, 94, 95, 96, 97, 98, 99, 100, 101, 102, 103, 104, 105] (S4 Table), we estimated altitude limits of distributions for *Alopoglossus festae* (85 localities), *A*. *viridiceps* (9 localities), *Bothrops osbornei* (16 localities), *B*. *punctatus* (18 localities), *Pristimantis crenunguis* (22 localities), *P*. *labiosus* (47 localities), *P*. *luteolateralis* (39 localities), *P*. *mindo* (12 localities), *P*. *subsigillatus* (45 localities), *P*. *walkeri* (51), and two undescribed species of *Pristimantis* (17 localities).

## Results

### Molecular analyses

**Fig 2:**
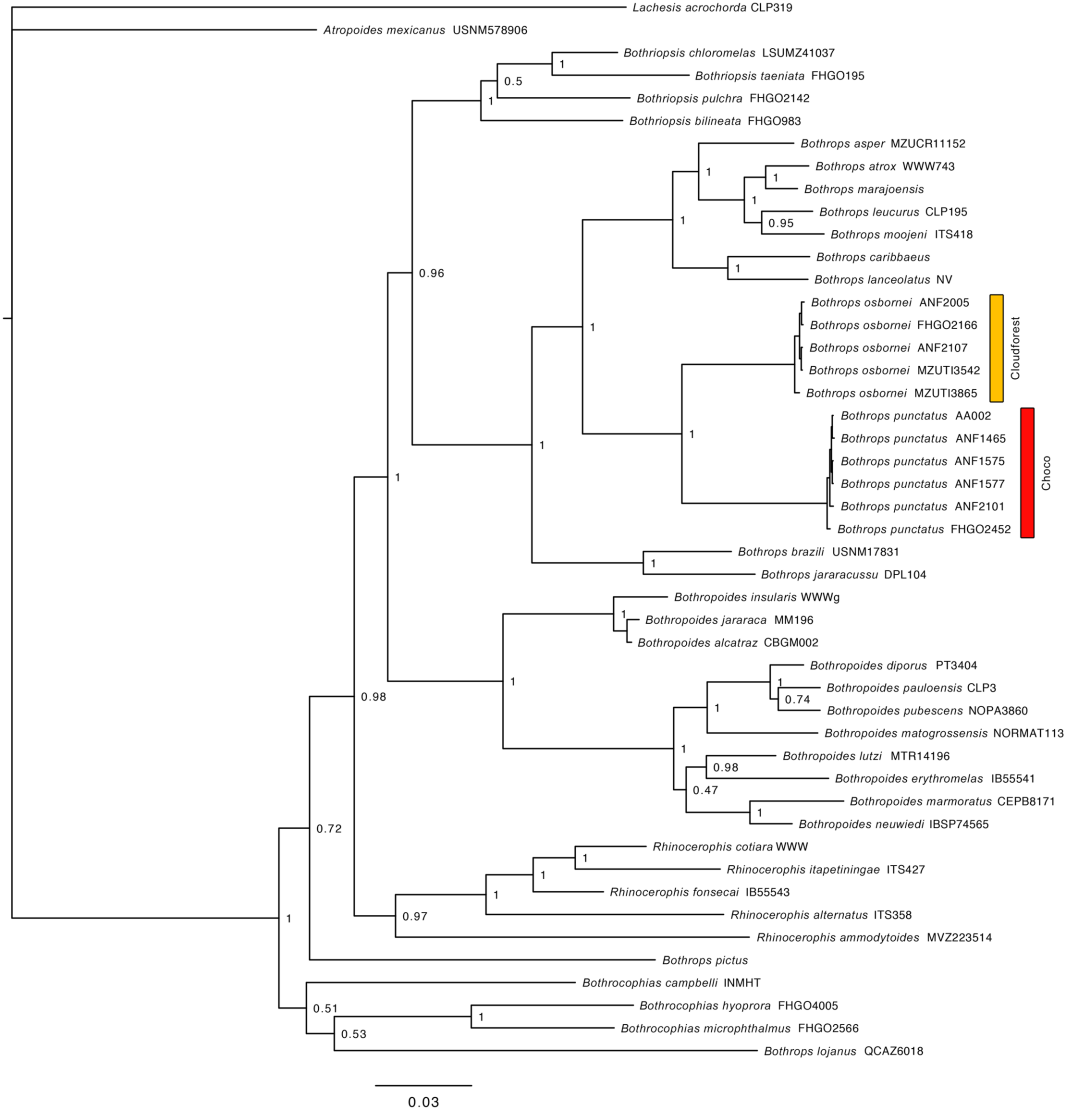
Bayesian consensus phylogram depicting relationships within bothropoid pitvipers. The phylogram was derived from analysis of 2908 bp of mitochondrial DNA (gene fragments 12S, 16S, Cytb and ND4). Posterior probabilities and voucher numbers are shown.

**Fig 3:**
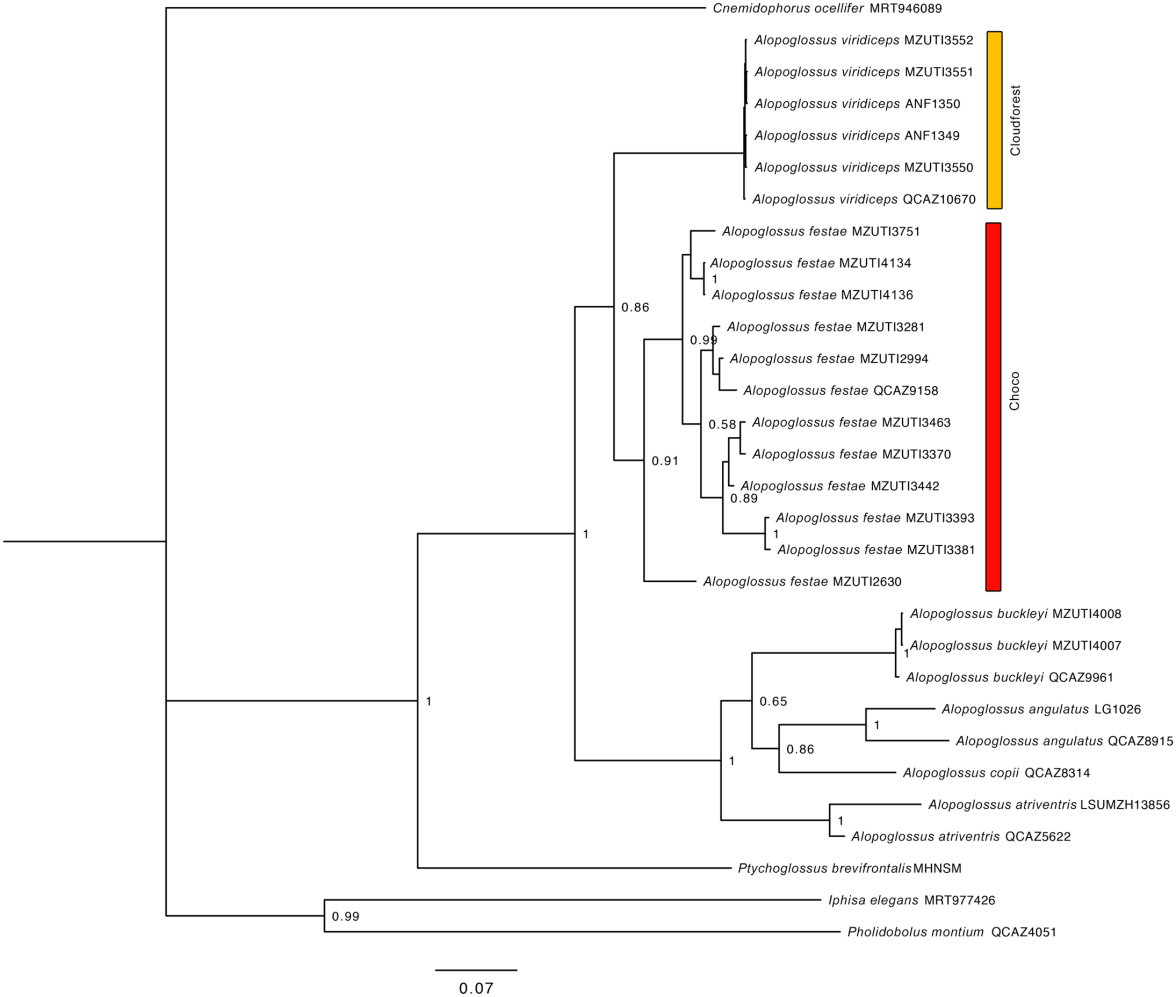
Bayesian consensus phylogram depicting relationships within the genus *Alopoglossus*. The phylogram was derived from analysis of 1139 bp of mitochondrial DNA (gene fragments 12S, 16S, Cytb and ND4). Posterior probabilities and voucher numbers are shown.

**Fig 4:**
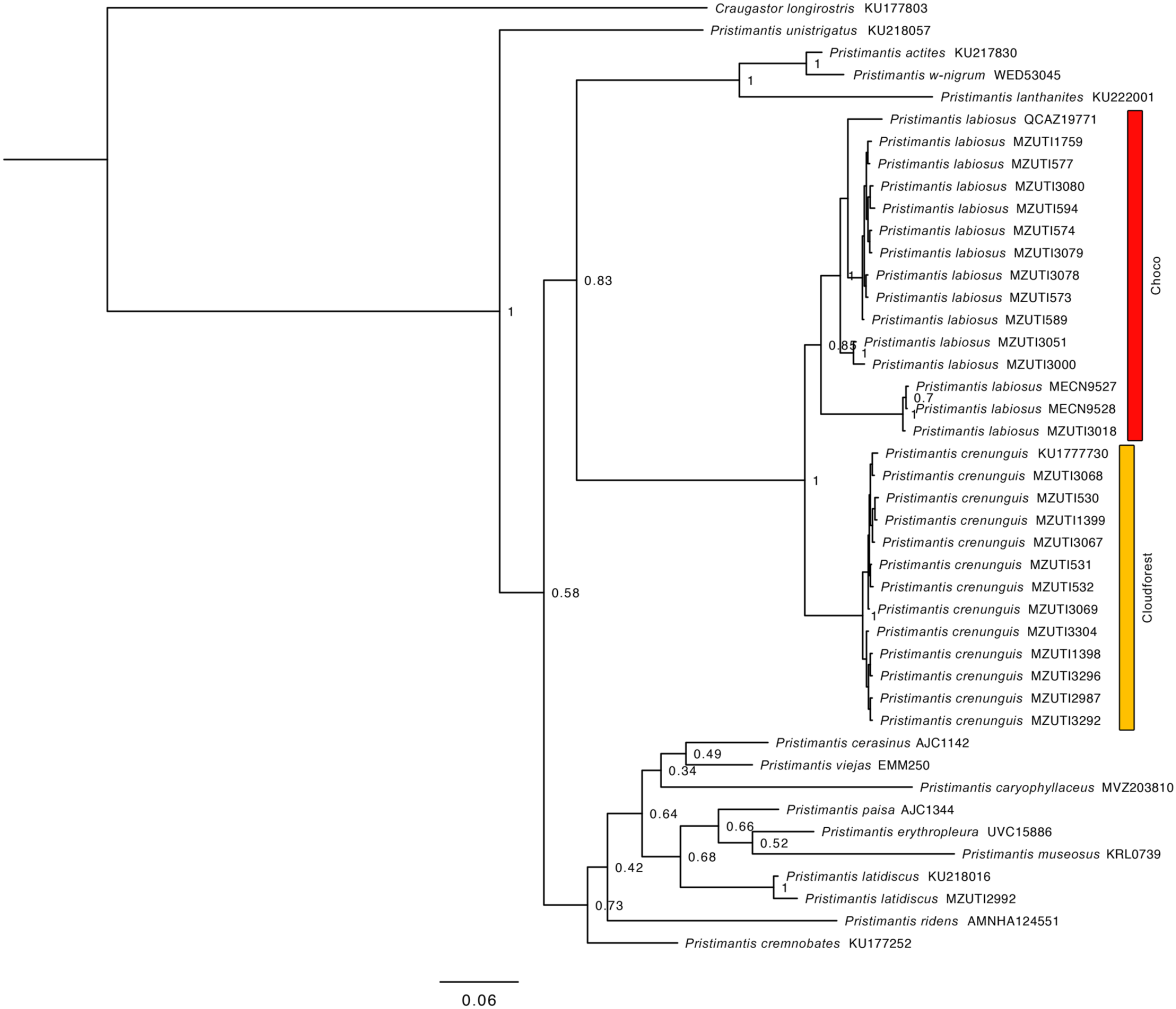
Bayesian consensus phylogram depicting relationships within the *Pristimantis* (*Hypodictyon*) *rubicundus* species series. The phylogram was derived from analysis of 1032 bp of mitochondrial DNA (gene fragments 12S and 16S). Posterior probabilities and voucher numbers are shown.

**Fig 5:**
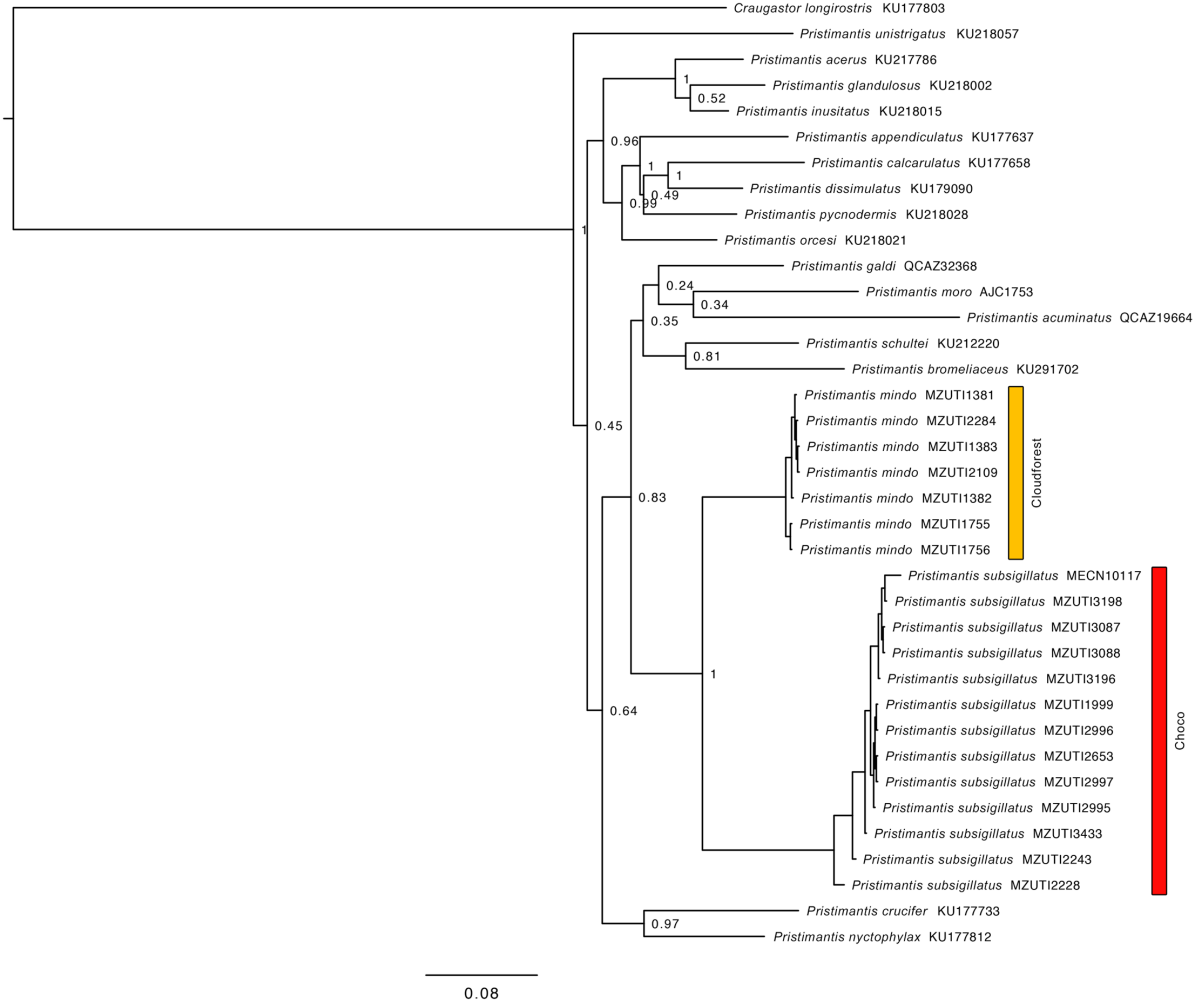
Bayesian consensus phylogram depicting relationships within the *Pristimantis lacrimosus* species group. The phylogram was derived from analysis of 2598 bp of mitochondrial DNA (gene fragments 12S and 16S). Posterior probabilities and voucher numbers are shown.

**Fig 6:**
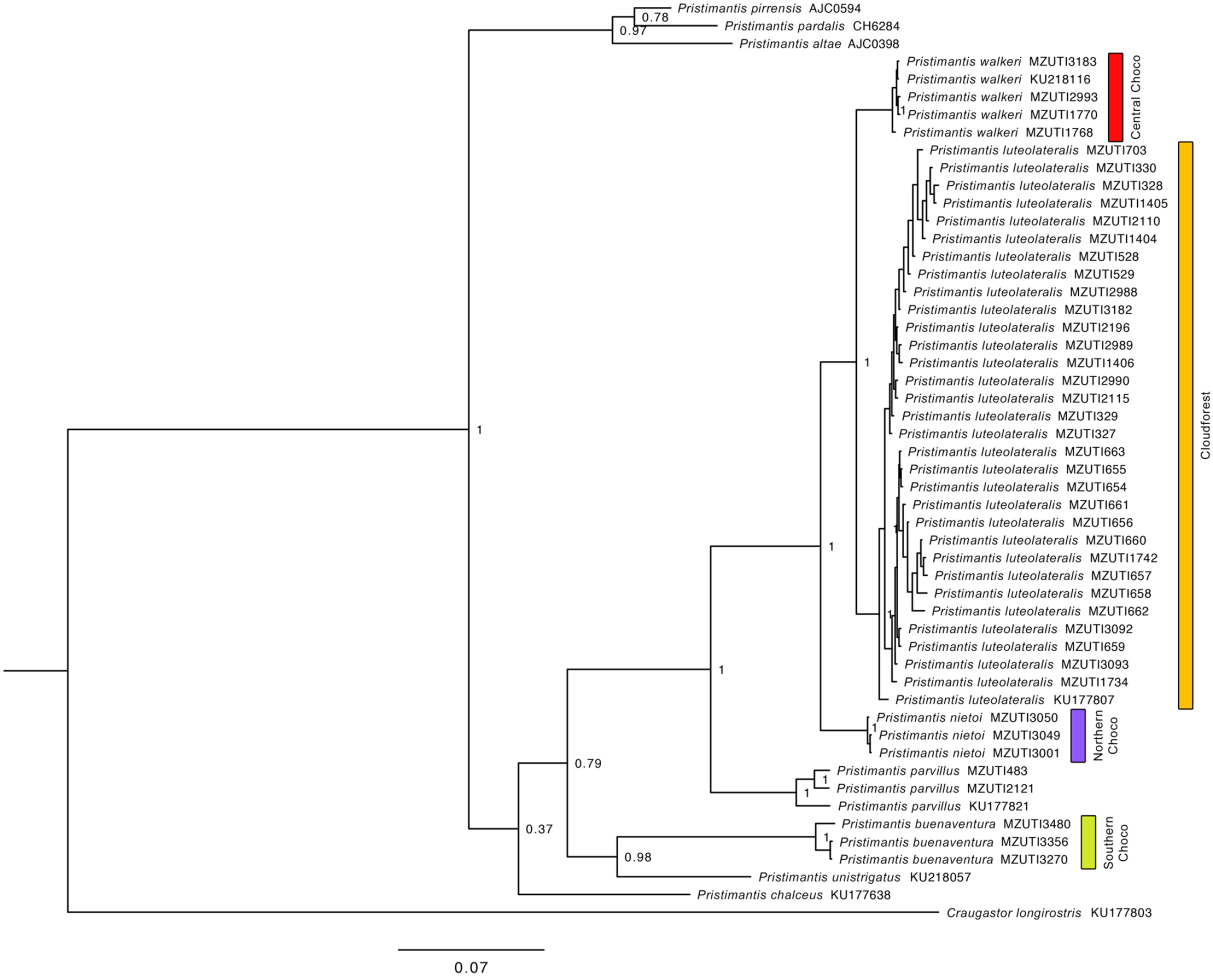
Bayesian consensus phylogram depicting relationships of the yellow-groined Trans-Andean *Pristimantis* of Ecuador. The phylogram was derived from analysis of 1905 bp of mitochondrial DNA (gene fragments 12S and 16S). Posterior probabilities and voucher numbers are shown.

The resulting hypotheses of species relationships and support four our five mitochondrial phylogenetic trees is similar to numerous recent works (Table 2, Figs 2–6). In agreement with previous results (see recent studies under Table 2), all five studied species pairs were recovered as sister species. In all cases, comparisons of a fragment of the mitochondrial genome between the species pairs shows greater interspecific than intraspecific genetic distance (Table 2). Intraspecific variation was 2% or lower in all cases except *Alopoglossus festae* and *Pristimantis labiosus*.

**Table 2:** Summary of mtDNA genetic distances, and phylogenetic relationship among studied species pairs. Figure numbers and relevant literature are included.

	Genetic distance				
Species pair	Gene fragment analyzed	Between species	Within species	Species relationship	Figure
*B*. *osbornei*, *B*. *punctatus*	759bp of NADH 4	7.6%	0%	Sister species	2
*A*. *festae*, *A*. *viridiceps*	596bp of NADH 4	12.4–13.4%	0–9.6%, 0%	Sister species	3
*P*. *crenunguis*, *P*. *labiosus*	495bp of 16S	5.9–8.1%	0–0.6%, 0–6.5%	Sister species	4
*P*. *mindo*, *P*. *subsigillatus*	695bp of 16S	10.4–10.9%	0–0.4%, 0–2.0%	Sister species	5
*P*. *luteolateralis*, *P*. *walkeri*	731bp of 12S	2.9–4.5%	0–0.7%, 0–0.1%	Sister species	6

In topology and support, our phylogenetic tree of yellow-groined Trans-Andean *Pristimantis* of Ecuador is similar to other recent studies (Table 2, Fig 6). However, as currently circumscribed [4, 22], *P*. *walkeri* is paraphyletic, with *P*. *luteolateralis* and *P*. *parvillus* nested within *P*. *walkeri*. To cope with this problem and to accurately reflect their distinct evolutionary histories, we treat each of the three clades (currently recognized as *P*. *walkeri*) as distinct species: *P*. *nietoi* **new species**, *P*. *walkeri sensu stricto* and *P*. *buenaventura* **new species** (together referred to as the *P*. *walkeri* species complex). As well as in other studies (Table 2), the yellow-groined rainfrogs *Pristimantis luteolateralis*, *P*. *parvillus* and *P*. *walkeri* form a strongly supported clade (Fig 6). Our study shows that *P*. *nietoi* belongs to the complex, but *P*. *chalceus*, *P*. *esmeraldas* and *P*. *buenaventura* do not. A comparison of a 731-bp long fragment of the mitochondrial 12S gene between *P*. *walkeri sensu stricto* and *P*. *nietoi* show a genetic distance of 5.2–5.5%, whereas sequence variation within *P*. *nietoi* is 0–0.1%.

### Distribution maps

**Fig 7:**
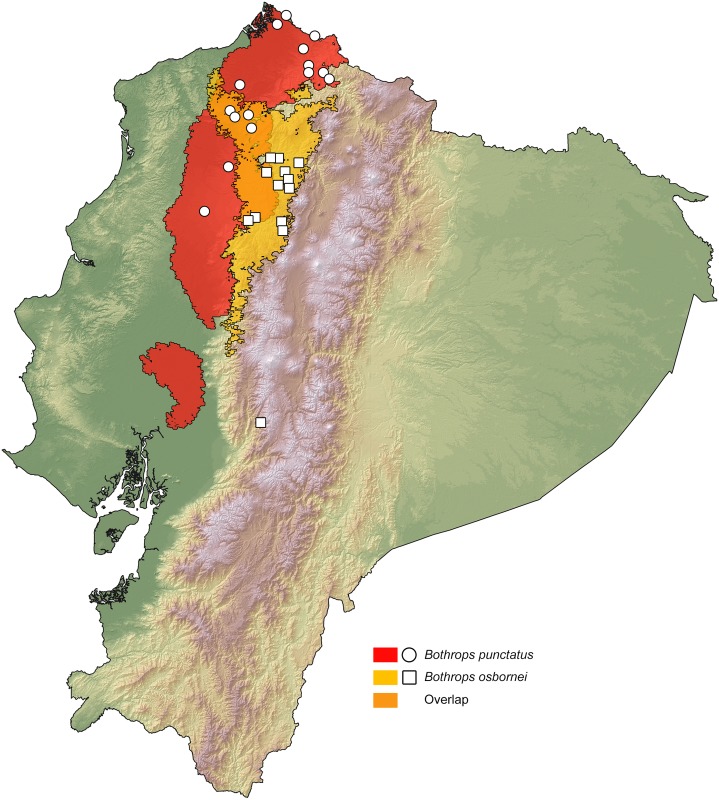
Distribution of the sister species *Bothrops osbornei* and *B*. *punctatus* in Ecuador. White dots represent known localities. Each colored area is a geographic representation of the suitable environmental conditions for one of the clades recovered in the phylogeny of Fig 2.

**Fig 8:**
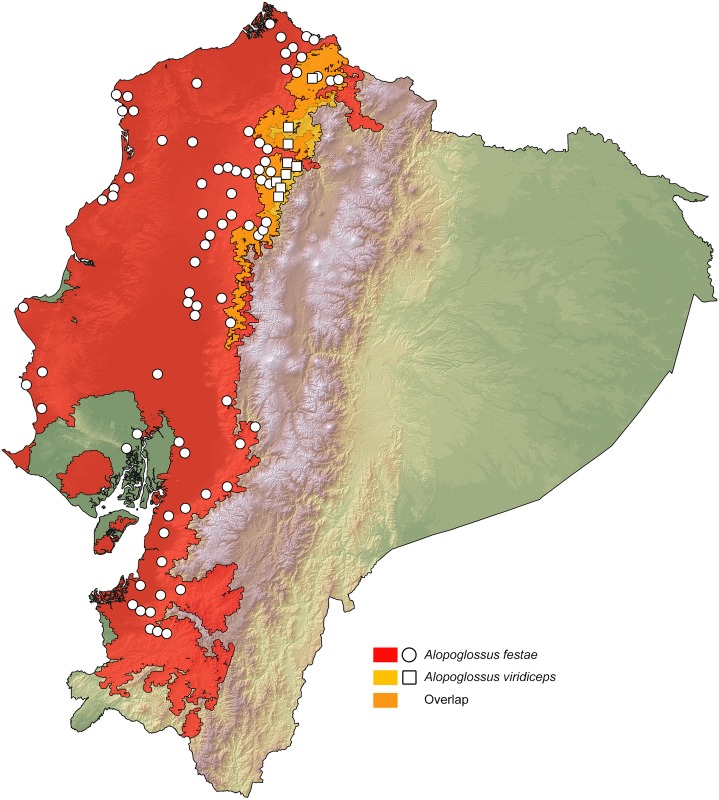
Distribution of the sister species *Alopoglossus festae* and *A*. *viridiceps* in Ecuador. White dots represent known localities. Each colored area is a geographic representation of the suitable environmental conditions for one of the clades recovered in the phylogeny of Fig 3.

**Fig 9:**
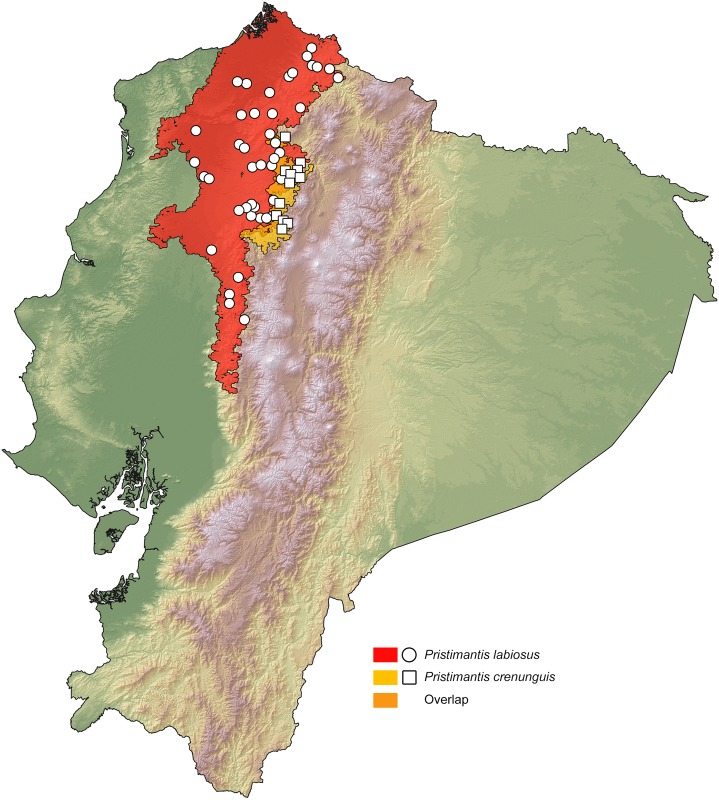
Distribution of the sister species *Pristimantis labiosus* and *P*. *crenunguis* in Ecuador. White dots represent known localities. Each colored area is a geographic representation of the suitable environmental conditions for one of the clades recovered in the phylogeny of Fig 4.

**Fig 10:**
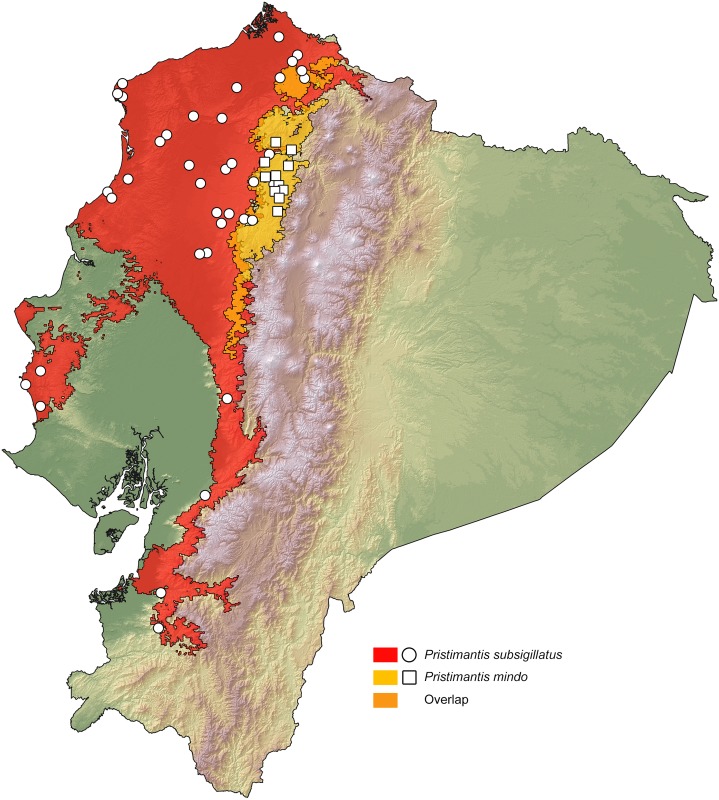
Distribution of the sister species *Pristimantis mindo* and *B*. *subsigillatus* in Ecuador. White dots represent known localities. Each colored area is a geographic representation of the suitable environmental conditions for one of the clades recovered in the phylogeny of Fig 5.

**Fig 11:**
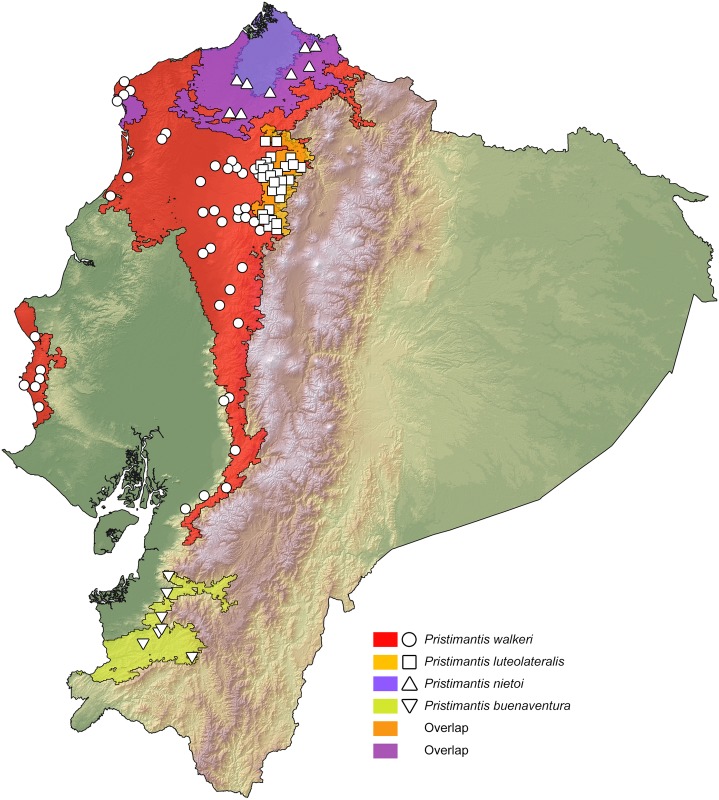
Distribution of *Pristimantis buenaventura*, *P*. *luteolateralis*, *P*. *nietoi* and *P*. *walkeri* in Ecuador. White dots represent known localities. Each colored area is a geographic representation of the suitable environmental conditions for one of the clades recovered in the phylogeny of Fig 6.

Our resulting distribution maps update previous works (see Table 3), increasing the number of known localities of occurrence for the studied taxa (S4 Table) and show a distinct geographical separation between sister species (Figs 7–11). In all cases, however, the predicted areas of suitable habitat for each of the studied sister species overlap with each other. They also overlap between 51.7% and 79.8% (see Table 3) with one of the main vegetation zones of northwestern Ecuador (Fig 1). In those species with primarily lowland distribution, their predicted areas of distribution overlap mainly with evergreen lowland forest or evergreen foothill forest, whereas their highland sister species overlap mainly with evergreen lower-montane forest (Table 3).

**Table 3:** Summary of key biogeographic traits among studied species pairs. Figure numbers and relevant literature are included. Abbreviations correspond to the vegetation zones of Fig 1. ELF = evergreen lowland forest; EFF = evergreen foothill forest; ELMF = evergreen lower-montane forest.

Species	Percentage overlap of ENM with main vegetation zones	Sympatry with sister species	ENM overlap between sister species	Altitude limits (masl)	Figure
*Bothrops osbornei*	53.77% ELMF, 31.14% EFF	No	19.11%	775–1657	7
*B*. *punctatus*	66.04% ELF, 26.63% EFF	No	19.11%	15–864	7
*Alopoglossus festae*	52.22% ELF, 28.04% EFF	No	5.96%	3–1377	8
*A*. *viridiceps*	70.33% ELMF, 21.37% EFF	No	5.96%	1165–1879	8
*Pristimantis crenunguis*	79.79% ELMF	No	1.71%	1165–1793	9
*P*. *labiosus*	51.66% ELF, 41.27% EFF	No	1.71%	63–1161	9
*P*. *mindo*	72.34% ELMF, 25.83% EFF	Yes	7.07%	1056–1810	10
*P*. *subsigillatus*	57.92% EFF, 54.09% ELF	Yes	7.07%	27–1092	10
*P*. *luteolateralis*	76.69% ELMF, 38.68% EFF	No	4.67%	905–1879	11
*P*. *walkeri*	38.72% ELF, 27.28% EFF	No	4.67%	27–1155	11

For the studied *Bothrops* species, we found that their elevational limits overlap latitudinally (Table 3), but with *B*. *punctatus* occurring at higher elevations north of the known distribution of *B*. *osbornei* (Fig 7). A similar scenario was observed for the studied *Alopoglossus* species, with *A*. *festae* occuring at elevations higher than 1165 m only north and south of the known distribution of *A*. *viridiceps* (Fig 8).

For *Pristimantis luteolateralis*, our resulting distribution map greatly expands that of Lynch and Duellman [22], and closely resembles that of Arteaga *et al*. [4]. Unlike previous works [4, 22], our distribution map of *P*. *walkeri sensu stricto* shows that it is endemic to the evergreen lowland and foothill forests of central Ecuador [55], whereas the northern and southern portion of its previously reported range now corresponds to that of *P*. *nietoi* **new species** and *P*. *buenaventura* **new species**, respectively.

### Systematics

**Fig 12:**
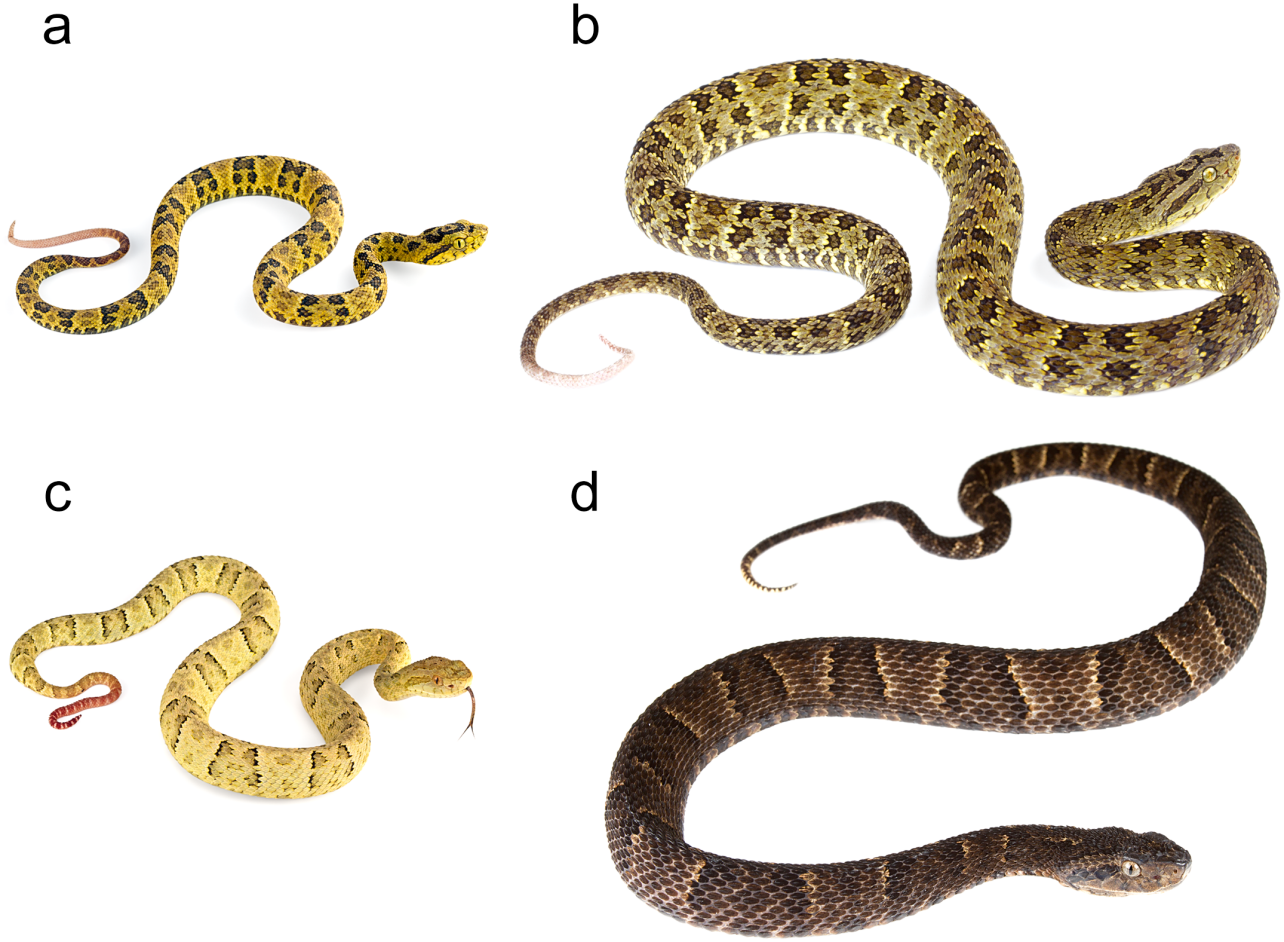
Morphological variation within sampled *Bothrops* species. (a) Juvenile of *B*. *punctatus* (ANF 1575). (b) Adult (ANF 2101) of *B*. *punctatus*. (c) Juvenile of *B*. *osbornei* (ANF 2005). (d) Adult of *B*. *osbornei* (ANF 2767).

Several authors [4, 88, 106] have considered *Bothrops punctatus* and *B*. *osbornei* to be conspecific. Our results at the molecular and ecological level now support the view of other authors [56, 89, 100] who have used morphological data to support the validity of *B*. *osbornei*. Although similar in external morphology and scale counts [56, 107] our sampled individuals show subtle but consistent differences in coloration (Fig 12). We found that *B*. *osbornei* has a dorsal pattern of dark trapezoidal blotches, whereas *B*. *punctatus* has a pattern of spots arranged in in the form of squares [4].

**Fig 13:**
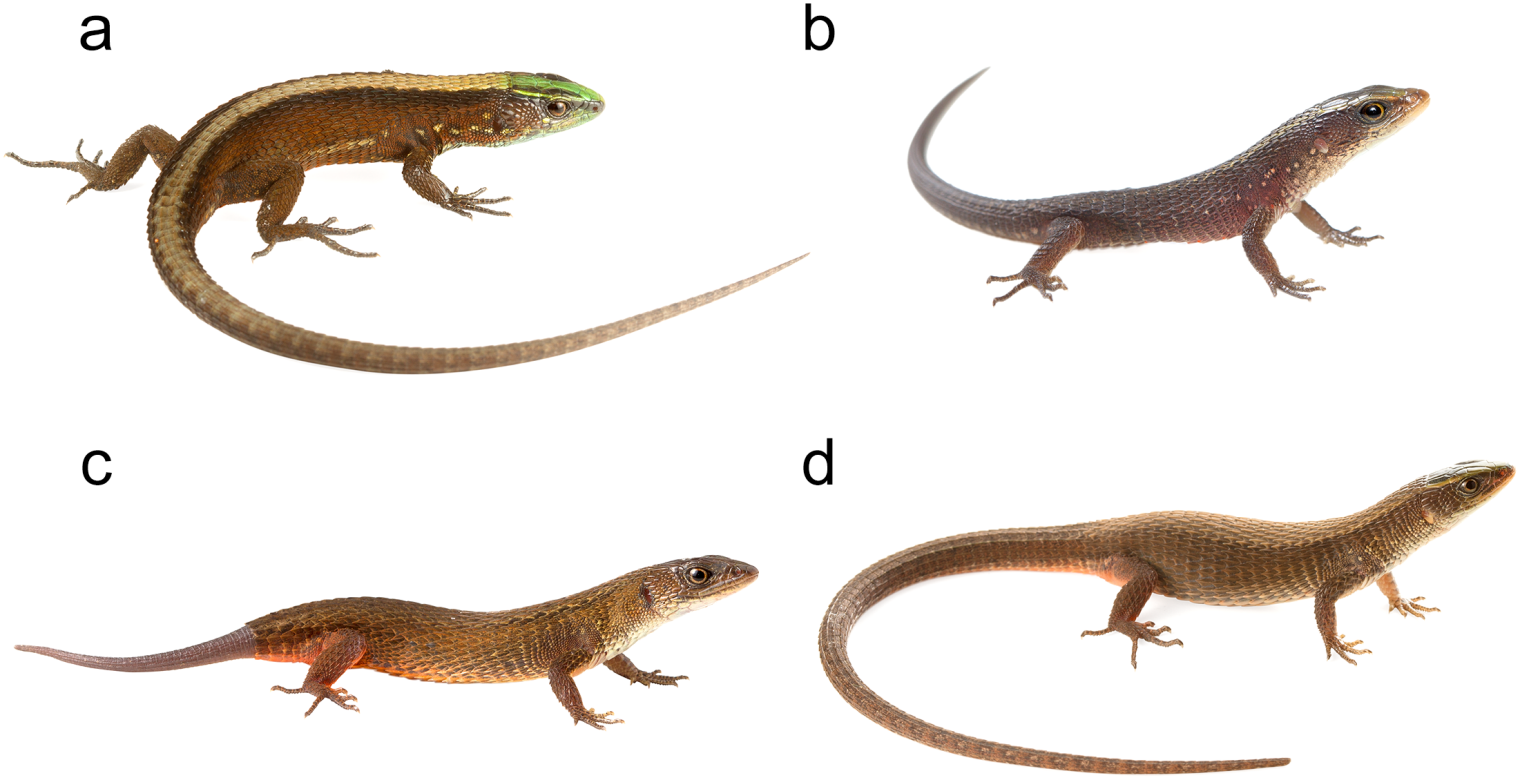
Morphological variation within sampled *Alopoglossus* species. (a) Adult male of *A*. *viridiceps* (MZUTI 3552). (b) Juvenile of *A*. *festae* (MZUTI 2630). (c) Adult of *A*. *festae* (MZUTI 2994). (d) Adult female of *A*. *festae* (MZUTI 3370).

Based on morphological characters, *Alopoglossus viridiceps* resambles its sister species *A*. *festae* [23] (Fig 13). This similarity might explain why several specimens of *A*. *viridiceps* housed at MZUTI and MECN, were previously identified as *A*. *festae*. Collections of the latter species from the highlands of Pichincha and Imbabura housed at the AMNH might actually represent *A*. *viridiceps*.

**Fig 14:**
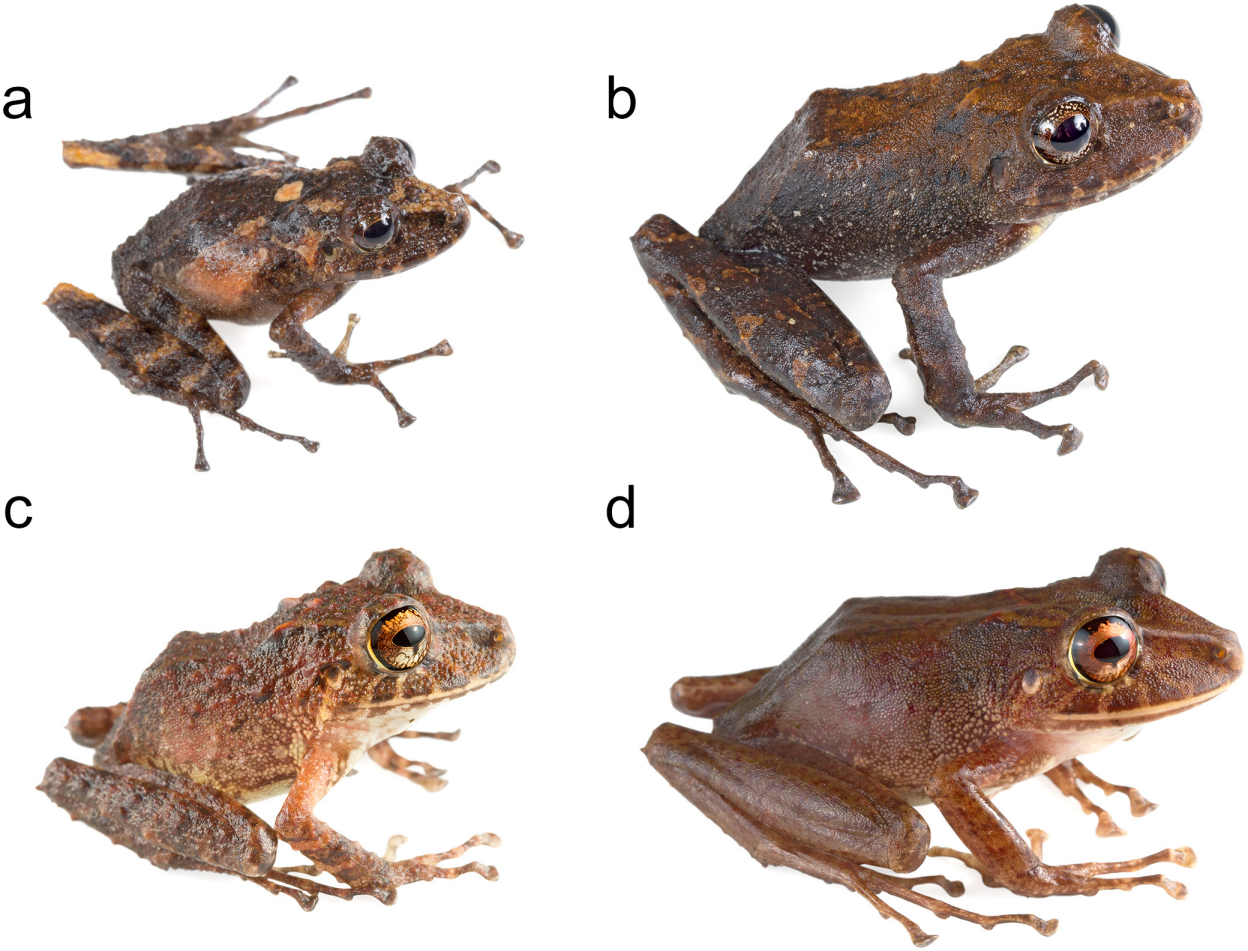
Morphological variation within *Pristimantis crenunguis* and *P*. *labiosus*. (a) Juvenile of *Pristimantis crenunguis* (Not vouchered). (b) Adult of *P*. *crenunguis* (Not vouchered). (c) Juvenile of *P*. *labiosus* (MZUTI 3511). (d) Adult of *P*. *labiosus* (Not vouchered).

Based on morphological characters, *Pristimantis labiosus* is most closely related to *P*. *crenunguis* [4, 22, 108] (Fig 14). Literature [98, 109] and museum records of *P*. *labiosus* above 1200 m likely correspond to *P*. *crenunguis*. Three specimens (MECN 9527, 9528, MZUTI 3018; Fig 4) share the majority of diagnostic characters of *P*. *labiosus* (as described by Lynch and Duellman [22]), but are distinct in external coloration from the rest of series examined by us (S3 Table).

**Fig 15:**
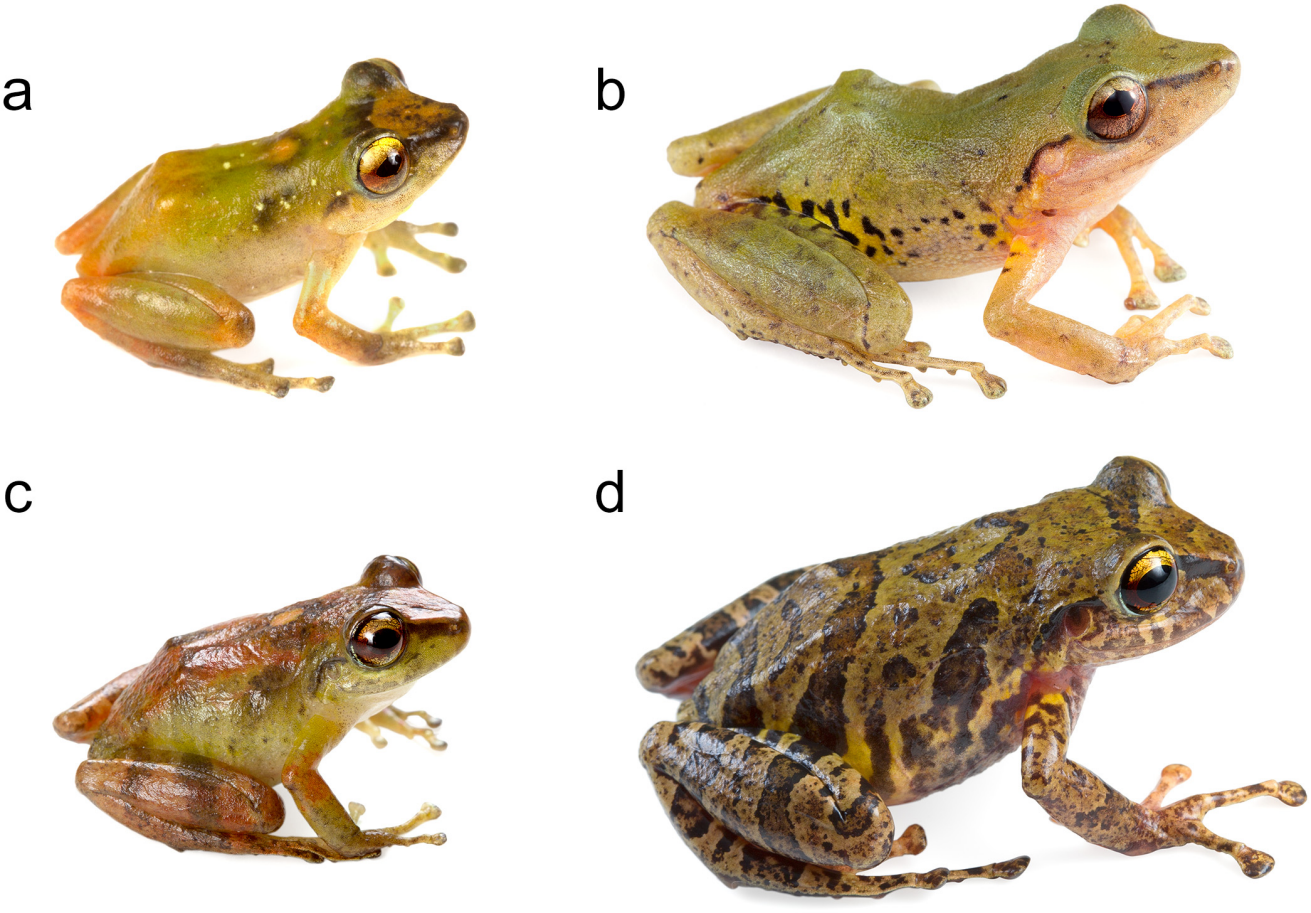
Morphological variation within *Pristimantis subsigillatus* and *P*. *mindo*. (a) Adult male of *Pristimantis subsigillatus* (MZUTI 2228). (b) Adult female of *P*. *subsigillatus* (MZUTI 2653). (c) Adult male of *P*. *mindo* (MZUTI 1382). (d) Adult female of *P*. *mindo* (MZUTI 1766).

Based on morphological characters, *Pristimantis subsigillatus* is most closely related to *P*. *mindo* [4] (Fig 15). This similarity might explain why several specimens of *P*. *mindo* housed at MZUTI and MECN, were previously identified as *P*. *subsigillatus*. Literature and museum records of *P*. *subsigillatus* above 1200 m likely correspond to *P*. *mindo*. Although not examined by us, it appears that one *Pristimantis subsigillatus* (KU 218147) from a previous study [110] may have been misidentified, and is actually a *P*. *nyctophylax*.

**Fig 16:**
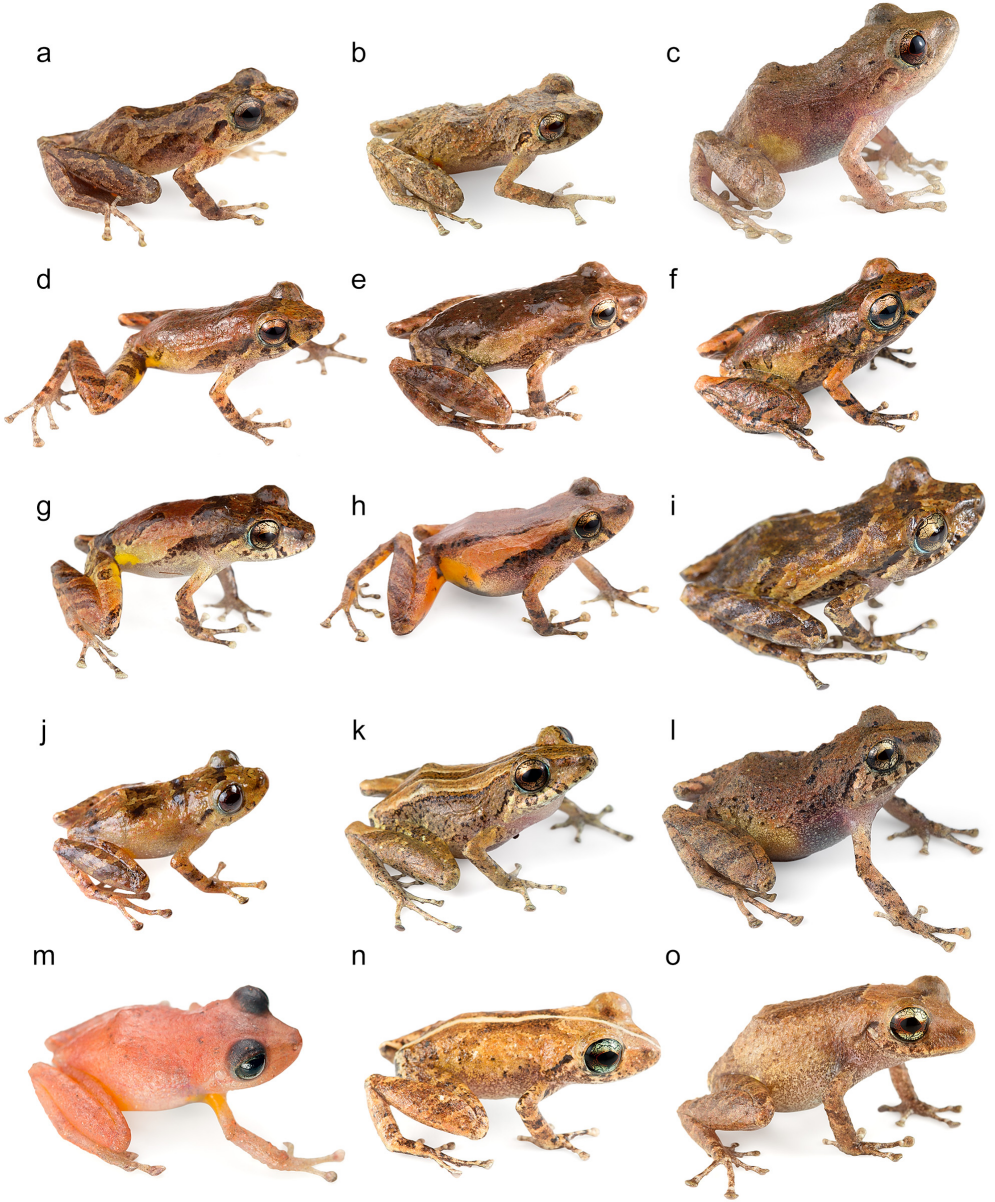
*Pristimantis walkeri* species complex and similar species. (a) Adult male paratype of *Pristimantis buenaventura* (MZUTI 3270). (b) Adult male holotype of *P*. *buenaventura* (MZUTI 3480). (c) Adult female paratype of *P*. *buenaventura* (MZUTI 3356). (d) Adult male holotype of *P*. *nietoi* (MZUTI 3913). (e) Adult male paratype of *P*. *nietoi* (MZUTI 3914). (f) Adult male paratype of *P nietoi* (MZUTI 3915). (g) Adult male of *P*. *luteolateralis* (MZUTI 3092. (h) Adult male of *P*. *luteolateralis* (MZUTI 3904). (i) Adult female of *P*. *luteolateralis* (Not vouchered). (j) Adult male of *P*. *parvillus* (Not vouchered). (k) Adult male of *P*. *walkeri* (MZUTI 1768). (l) Adult female of *P*. *walkeri* (Not vouchered). (m) Adult female of *P*. *scolodiscus* (Not vouchered). (n) Adult male of *P*. *esmeraldas* (MZUTI 3545). (o) Adult female of *P*. *esmeraldas* (MZUTI 3375).

Based on morphological characters, *Pristimantis walkeri* is most closely related to *P*. *luteolateralis* [4, 22, 91] (Fig 16). Some authors [4, 104] have confused intermediate elevation populations of *P*. *luteolateralis* with *P*. *walkeri sensu stricto*. Our genetic analyses of the *P*. *walkeri* species complex demonstrate the existence of at least three distinct lineages that deserve full-species status. By examining specimens of the *P*. *walkeri* species complex, we found consistent morphological differences among the three genetic lineages (see below). One of these, *P*. *walkeri sensu stricto*, is herein restricted to the populations in the central Pacific lowlands of Ecuador, where the type locality of *P*. *walkeri* lies (Las Palmas). Populations of the type locality were included in the molecular analyses, and they are nested within the clade herein defined as *P*. *walkeri sensu stricto*. Below, we describe the new species.

### Phylogeographic patterns

Our species distribution models (Figs 7–11) for the five sister-species pairs, their morphological similarities (Figs 12–16), and their position in the mitochondrial phylogenetic tree (Fig 2) suggests that species originated by allopatric or parapatric speciation. For some sister-species pairs, this scenario was already suggested: *Alopoglossus festae* and *A*. *viridiceps* by Torres-Carvajal and Lobos [23]; *Pristimantis crenunguis* and *P*. *labiosus* by Lynch and Duellman [22] and Arteaga *et al*. [4]; and *P*. *mindo* and *P*. *subsigillatus* by Arteaga *et al*. [4]. These assumptions were made based on the similarities in body size, outer morphology, microhabitat, and the adjacent ranges of distribution of the sister-species pairs.

Published [23, 99] and museum (S3 Table) distribution records of *A*. *viridiceps* are located south of the Río Lita and north of the Río Toachi (Figs 1 and 8). These two rivers might have acted as effective barriers for latitudinal dispersal of *A*. *viridiceps*. Although most samples of *A*. *festae* show a degree of geographical structure in accordance to the presence of main river systems (e.g. north and south of the Río Chimbo), the most genetically divergent sample (MZUTI 2630) (Fig 3) is geographically isolated from all other samples not by river systems but by an elevation gradient along the Coastal Cordillera.

The existence of at least two genetically distinct (Fig 4), but not geographically structured, lineages within *Pristimantis labiosus* suggest a more complex scenario of diversification than just one event of vicarance between *P*. *labiosus* and *P*. *crenunguis*. Samples of *P*. *labiosus* of evergreen lowland forest north of the Río Esmeraldas (MZUTI 3000, 3051) form a clade distinct from samples of the same species inhabiting evergreen foothill forests south of the Río Esmeraldas (Figs 1 and 4). Although most samples of *P*. *crenunguis* show a degree of geographical structure (north and south of the Río Guayllabamba), some samples from south of the Río Guayllabamba (MZUTI 1398, 2987) are nested within the samples north of that river (Fig 4). All published [22, 91, 104, 111] distribution records of *P*. *crenunguis* are located south of the Río Lita and north of the Río Toachi (Figs 1 and 9). These two rivers might have acted as effective barriers for latitudinal dispersal of *P*. *crenunguis*.

The two known populations of *Pristimantis mindo* are reciprocally monophyletic and exhibit greater genetic distance from each other than within populations (Table 2). This pattern is best explained by the presence of the Río Guayllabamba (Fig 1) that seems to be acting as a dispersal barrier. On the contrary, *P*. *subsigillatus* is not geographically structured, with samples north and south of the different river systems not clustering together (Fig 5).

The topology of our mitochondrial phylogenetic tree (Fig 6) suggests that the clade containing the *P*. *walkeri* species complex originated North of the Río Esmeraldas, where *P*. *nietoi* **new species** is currently extant. Lynch and Duellman [22] and Arteaga *et al*. [4] suggested that *Pristimantis walkeri* and *P*. *luteolateralis* are altitudinal replacements of each other. Our species distribution models and the mitochondrial phylogenetic tree support this relationship when the name *P*. *walkeri* is restricted to the populations south of the Río Esmeraldas. We found that populations of *P*. *buenaventura* **new species** to be more closely related to *P*. *unistrigatus* than to *P*. *walkeri*, suggesting no direct common ancestry between *P*. *buenaventura* and *P*. *walkeri*.

#### *Pristimantis* *nietoi*

Arteaga, Pyron, Peñafiel, Romero-Barreto, Culebras, Bustamante, Yánez-Muñoz & Guayasamin **sp. nov.** urn:lsid:zoobank.org:act:6B7476EA-5DC8-4746-A4F2-FD45F363E9C3

#### Common English name

Nieto’s Rainfrog

#### Common Spanish name

Cutín de Nieto

#### Holotype

**Fig 17:**
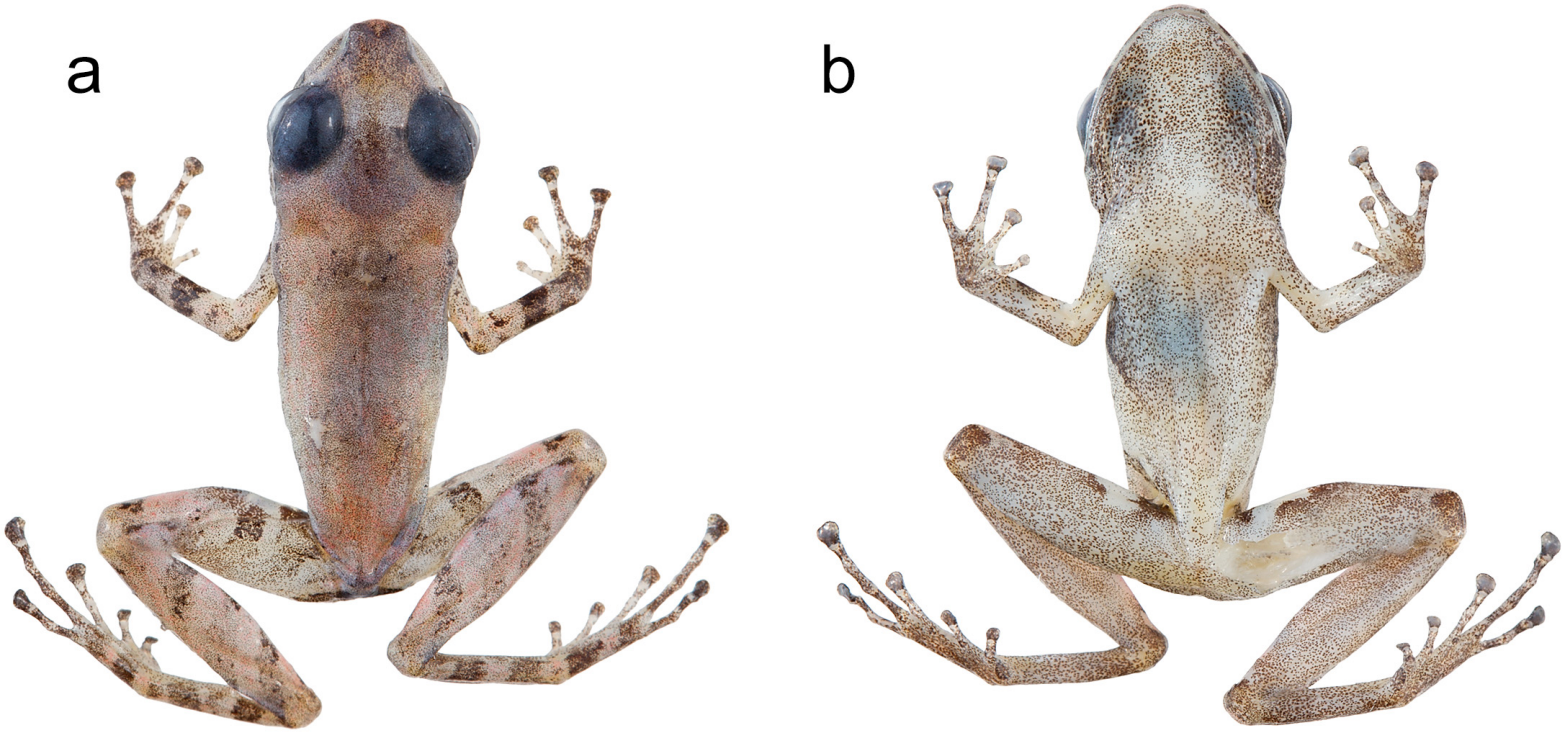
Adult male holotype of *Pristimantis nietoi*. MZUTI 3913, SVL 16.3 mm.

**Fig 18:**
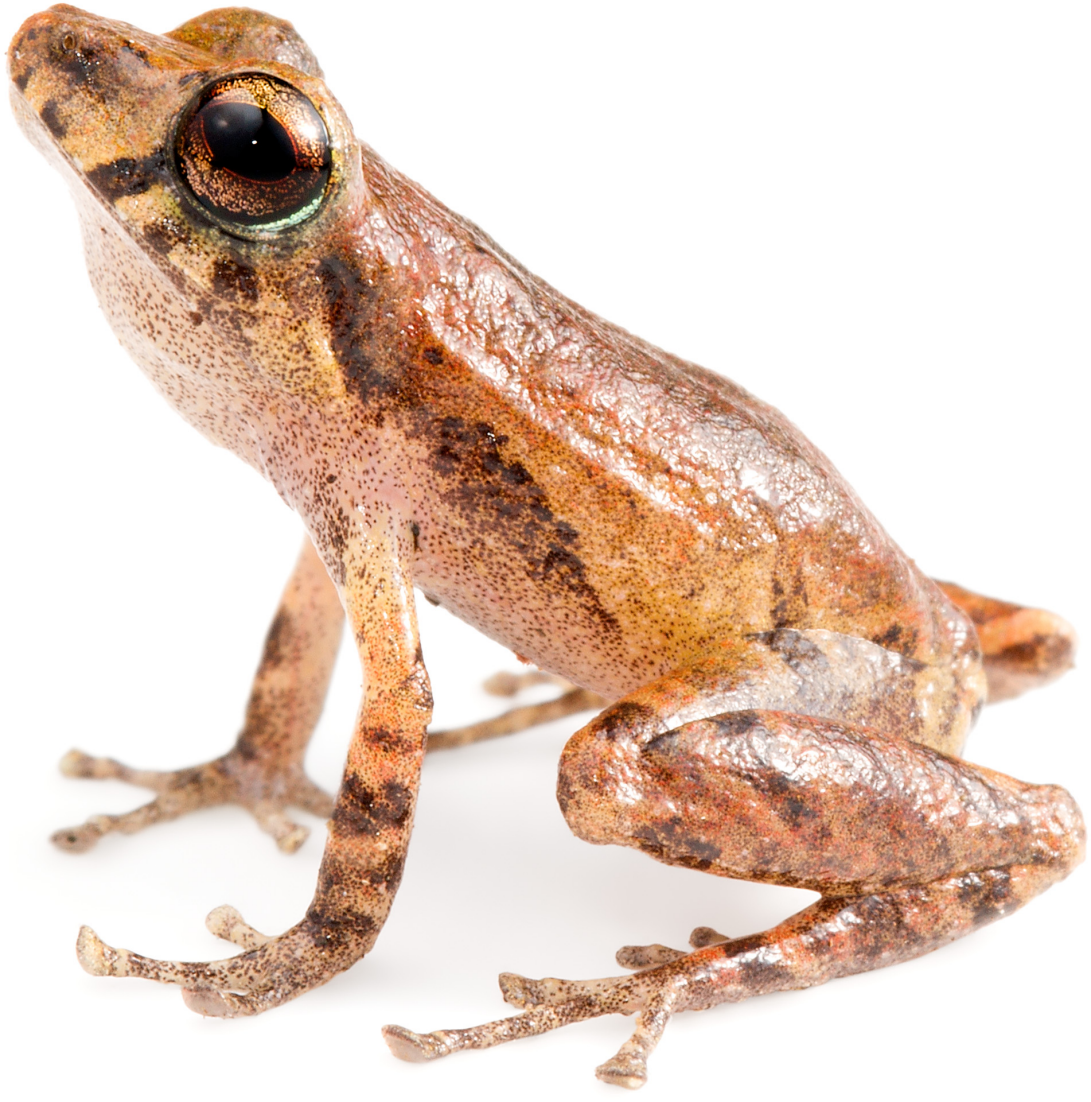
*Pristimantis nietoi* in life. MZUTI 3913, SVL 16.3 mm, adult male, holotype.

MZUTI 3913, an adult male (Figs 17 and 18) obtained by Jaime Culebras on November 11, 2014, at Reserva Itapoa (00.51307 N 79.13401 W; 321 m), Esmeraldas province, Ecuador.

#### Paratopotypes

Jaime Culebras and Lucas Bustamante collected seven specimens between June 2013 and November 2014. From these, two (MZUTI 3001,3049) (Fig 16d and 16f) are adult females; five (MZUTI 3050,3273,3914–16) are adult males.

#### Paratypes

One adult male (MZUTI 2231) was collected by Alejandro Arteaga on August 2012 at the vicinities of Reserva Río Canandé (00.47502 N 79.21889 W; 373 m), Esmeraldas province, Ecuador.

#### Diagnosis

The new species is placed in the genus *Pristimatis*, as diagnosed by Hedges *et al*. [51], based on phylogenetic evidence (Fig 6). It is included in a phenetic assemblage of Ecuadorian Trans-Andean *Pristimantis* characterized by their yellow to orange pigmentation in the hidden surfaces of the hind limbs (Fig 16; Table 4). *Pristimantis nieto* is diagnosed by having the following features. (1) Skin texture of the dorsum and venter finely shagreen (Fig 17). (2) Tympanic membrane absent, tympanic annulus evident, 34–40% the diameter of the eye, with upper rim obscured by supratympanic fold. (3) Snout 17–20% of SVL, rounded in dorsal view and rounded in profile. (4) Upper eyelid bearing low, non-conical tubercles. In females, upper eyelid width 68–80% of interorbital distance (60–83% of that distance in males). Cranial crests absent. (5) Dentigerous processes of vomers well developed, oblique in outline, each bearing 3–5 teeth. (6) Males with small subgular vocal sac and vocal slits, but no nuptial pads. (7) Finger I shorter than II. Discs on fingers expanded, rounded to slightly truncate, except for Finger I that is barely expanded. (8) Fingers bearing narrow lateral fringes. Outer palmar tubercle cordate and distally divided. Supernumerary tubercles low and indistinct. (9) Ulnar tubercles absent. (10) Heel and tarsal tubercles absent. (11) Toes bear narrow fringes but no webbing. Toe V slightly longer than Toe III. Toe discs expanded, rounded to slightly truncate. (12) Inner metatarsal tubercle elliptical, about three times the size of outer tubercle, which is rounded and low. Supernumerary plantar tubercles round and weakly developed. (13) In ethanol (Fig 17), the dorsum of *P*. *nietoi* is dull reddish-brown (rich reddish-brown in life; Figs 16 and 18) with dark, faint irregular markings, a dark supratympanic stripe and 1–3 supralabial bars; groin dark brown and enclosing irregular smoky-white blotches (bright yellow in life). Background color of throat, belly and ventrolateral surfaces dingy white, with different levels of fine brown mottling. In life, iris golden with black reticulations and crossed by a coppery median streak. (14) SVL in females 18.5–19.5 mm (mean = 19.0 ± 0.7; n = 2). In males 14.4–17.0 mm (mean = 15.9 ± 0.9; n = 7).

**Table 4:** Character states in the Ecuadorian Trans-Andean *Pristimantis* with yellow to orange pigmentation in the hidden surfaces of the hind limbs.

Species	Heel tubercles	Groin pattern	Oblique lateral stripe
*P*. *buenaventura*	Present, low	Orange spots outlined in black	Absent
*P*. *nietoi*	Present, low	Yellow blotches outlined in black	Present, faint
*P*. *esmeraldas*	Absent	Yellow blotches, sometimes absent	Absent
*P*. *luteolateralis*	Present, subconical	Yellow blotches outlined in black	Present, distinct
*P*. *parvillus*	Present, low	Large yellow oval spot	Absent
*P*. *scolodiscus*	Present, low	Large yellow oval spot	Absent
*P*. *walkeri*	Present, low	Yellow blotches outlined in black	Absent

#### Genetic characters

This species can be distinguished from its closest morphological relative, *Pristimantis walkeri*, by a sequence variation of 5.2–5.5% in a 731-bp long fragment of the mitochondrial 12S gene. Sampled specimens (MZUTI 3001, 3049–50) of *P*. *nietoi* form a cluster genetically divergent from the other members of the *P*. *walkeri* species complex (Fig 6).

#### Similar species

*Pristimantis nietoi* differs from other Trans-Andean yellow-to-orange groined members of the *P*. *unistrigatus* group and other morphologically similar congeners (Table 4) by having its yellow groin pigment in the form blotches outlined in black, low heel tubercles, and a faint oblique-lateral stripe. The most similar rainfrog species are *P*. *luteolateralis* (which has subconical heel tubercles) and *P*. *walkeri* (which lacks an oblique-lateral stripe). Moreover, the new species is smaller than any other in the group. Males are 14.4–17.0 mm in SVL and females are 18.5–19.5 mm in SVL, whereas males of *P*. *walkeri* are 16.8–22.8 mm in SVL and females, 22.6–26.7 mm. SVL in males of *P*. *luteolateralis* is 16.6–23.6 mm, and in females is 25.6–29.5 mm. In *P*. *parvillus*, males are 15.5–19.4 mm in SVL, and females are 18.4–25.9 mm in SVL [22].

#### Description of the holotype

Adult male (MZUTI 3913; Figs 17 and 18). Head slightly wider than body and slightly longer than wide. Upper eyelid bearing several feebly visible tubercles both in life and preserved. Head width 29% of SVL. Head length 39% of SVL. Snout 19% of SVL, rounded in dorsal view (Fig 17) and rounded in profile. Tongue longer than wide, with posterior half notched and not adherent to floor of mouth. Eye diameter slightly larger than eye–nostril distance. Nostrils not protuberant and directed anterolaterally. Canthus rostralis and loreal region weakly concave in profile. Upper eyelid width 80% of interorbital distance. Cranial crests and tympanic membrane absent, but tympanic annulus distinct and round. Two postrictal tubercles present, but barely visible. Choanae round and not concealed by palatal shelf of maxillary. Vomerine odontophores oblique in outline and about 50% longer than diameter of choana, located posteromedial to the choanae and separated medially by distance less than width of odontophore; each bearing 5 teeth. Vocal slits and median, subgular vocal sac present. Skin on all surfaces finely shagreen (Fig 17). Discoidal and thoracic folds absent (Fig 17). Cloacal sheath absent, but cloacal region bordered ventrally by low tubercles. Ulnar tubercles and fold absent; outer palmar tubercle present and distally divided. Subarticular tubercles round in section and supernumerary palmar tubercles indistinct. Fingers bear narrow lateral fringes. Finger I shorter than Finger II. Disc of Finger I barely expanded. All other discs expanded, twice the width of the proximal phalanx, and elliptical to slightly truncate. Ventral pads well defined by circumferential grooves. No nuptial pads are present. Tibia lenght 53% of SVL and foot length 40% of SVL. Inner metatarsal tubercle about 3 times the size of the outer tubercle. Subarticular tubercles round in section, but plantar supernumerary tubercles indistinct. Toes bearing narrow lateral fringes, but no webbing. Discs of Toe I barely expanded. All other toe discs expanded and rounded to slightly truncate. Toes have ventral pads well defined by circumferential grooves. Relative length of the toes is: I < II < III < V < IV. Toe V slightly longer than Toe III.

#### Measurements of holotype (in mm)

SVL 16.3; tibia length 8.7; foot length 6.5; head length 6.4; head width 4.8; eye diameter 2.4; interorbital distance 1.9; upper eyelid width 1.6; internarial distance 1.6; eye-nostril distance 2.0.

#### Coloration of holotype in preservative

Dorsal surfaces rosy brown with a fine dark speckling and a whitish rostral blotch (Fig 17). Dorsal surfaces of limbs banded with dark pigment; toe pads tinged with black. Three supralabial bars accompany a blackish supratympanic stripe. Background color of ventral surfaces dingy white with a gentle brown mottling overall.

#### Coloration of holotype in life (Figs 16 and 18)

Based on field notes by Alejandro Arteaga. Upper surfaces reddish brown with irregular areas of darker or lighter color, and transverse bars on the limbs. Flanks brownish cream with an oblique-lateral stripe running from behind the supratympanic stripe. Background color of ventral surfaces dingy white with a gentle brown mottling overall. Groin and hidden surfaces of hind limbs dark brown with irregular bright yellow blotches. Iris goldenrod with black reticulations and crossed by a coppery median streak.

#### Variation

Morphological variation is presented in Tables 5 and 6. Females are larger than males, but are otherwise identical to the males in coloration. MZUTI 3915 is dorsally darker than the rest of the series. Most samples lack dorsolateral folds, but this structure is present, albeit faintly visible, in MZUTI 3913 and 3950.

**Table 5:** Measurements (in mm) of adults of *Pristimantis nietoi*.

	Females (N = 2)	Males (N = 7)
SVL	18.5–19.5 (19.0 ± 0.7)	14.4–17.0 (15.9 ± 0.9)
Tibia length	9.9–10.4 (10.1 ± 0.3)	8.1–9.1 (8.6 ± 0.3)
Foot length	8.2–8.7 (8.5 ± 0.4)	6.1–6.9 (6.5 ± 0.3)
Head length	7.8–8.0 (7.9 ± 0.2)	6.0–7.0 (6.5 ± 0.3)
Head width	6.8–7.3 (7.0 ± 0.3)	4.8–6.4 (5.7 ± 0.5)
Interorbital distance	2.2–2.7 (2.5 ± 0.4)	1.8–2.2 (2.0 ± 0.1)
Upper eyelid width	1.8–1.8 (1.8 ± 0.1)	1.3–1.7 (1.5 ± 0.1)
Radioulna length	4.3–4.3 (4.3 ± 0.0)	3.3–4.0 (3.7 ± 0.2)
Eye-to-nostril distance	2.3–2.4 (2.4 ± 0.0)	1.6–2.1 (1.9 ± 0.2)
Snout-to-eye distance	3.6–3.7 (3.6 ± 0.1)	2.7–3.3 (3.0 ± 0.2)
Eye diameter	2.6–2.7 (2.7 ± 0.0)	2.1–2.6 (2.3 ± 0.2)
Tympanum diameter	0.9–1.0 (1.0 ± 0.0)	0.7–1.0 (0.9 ± 0.1)
Hand length	4.6–5.0 (4.8 ± 0.3)	3.7–4.4 (4.0 ± 0.3)
Finger I length	2.2–2.8 (2.5 ± 0.4)	1.9–2.5 (2.2 ± 0.2)

**Table 6:** Ranges of morphological proportions (in percentages) of adults of *Pristimantis nietoi*.

	Females (N = 2)	Males (N = 7)
Tibia length/SVL	53.3–53.4	50.2–61.1
Foot length/SVL	44.2–44.9	38.7–42.4
Foot length/tibia length	82.7–84.2	69.4–81.1
Head width/SVL	36.7–37.4	29.1–37.7
Head length/SVL	41.3–42.1	39.4–41.6
Head length/head width	110.6–114.9	108.1–135.4
Eye-to-nostril distance/eye diameter	86.6–90.8	62.1–95.1
Upper eyelid width/IOD	67.9–79.9	60.5–83.3
Radioulna length/SVL	22.0–23.1	21.2–25.1
Hand length/radioulna length	106.8–116.8	98.9–126.5
Finger I length/hand length	48.3–56.7	49.5–59.6

#### Natural history

Specimens of *Pristimantis nietoi* have been found active by night on vegetation 25–200 cm above the ground in primary and secondary evergreen lowland forest (Figs 1 and 15). Vocalizing males and females with mature eggs were found between June and November. At the type locality, *P*. *nietoi* is syntopic with *P*. *achatinus*, *P*. *esmeraldas*, *P*. *labiosus*, *P*. *latidiscus*, *P*. *parvillus* and *P*. *subsigillatus*.

#### Distribution

63–517 m. *Pristimantis nietoi* is endemic to the Chocoan lowlands of northwestern Ecuador (Fig 15). Besides the two localities of the type series, the species has been previously reported in seven localities under the name *P*. *walkeri*. See S4 Table.

#### Etymology

The specific epithet honors Raúl Nieto, for his life-long effort to protect the imperiled Choco forests and their biodiversity. Three of us (JC, LB and AA) have been lucky to enough to explore the forests of western Ecuador along with Raúl. In these trips, we have been inspired by Raúl’s passion to conserve Ecuador’s natural resources.

#### Conservation status

We consider *Pristimantis nietoi* to be Vulnerable following B1a IUCN criteria because its extent of occurrence is estimated to be 1,455 km^2^, its habitat is severely fragmented [112], and it is known to exist at no more than nine localities.

#### *Pristimantis* *buenaventura*

Arteaga, Pyron, Peñafiel, Romero-Barreto, Culebras, Bustamante, Yánez-Muñoz & Guayasamin, **sp. nov.** urn:lsid:zoobank.org:act:22FA1A2D-D7E2-4BDF-9089-867565EBB4D4

#### Common English name

Buenaventura Rainfrog

#### Common Spanish name

Cutín de Buenaventura

#### Holotype

**Fig 19:**
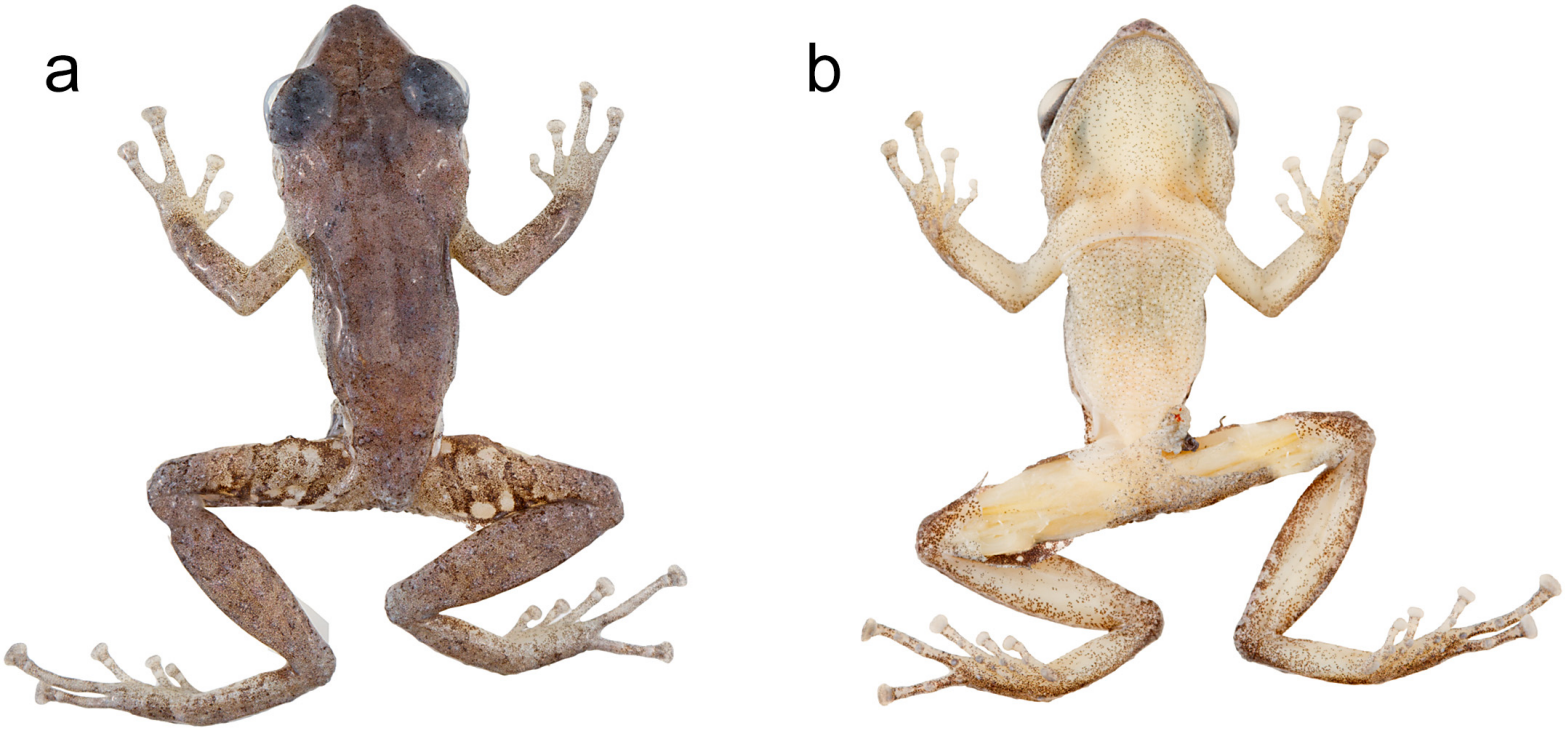
Adult male holotype of *Pristimantis buenaventura*. MZUTI 3480, SVL 19.2 mm.

**Fig 20:**
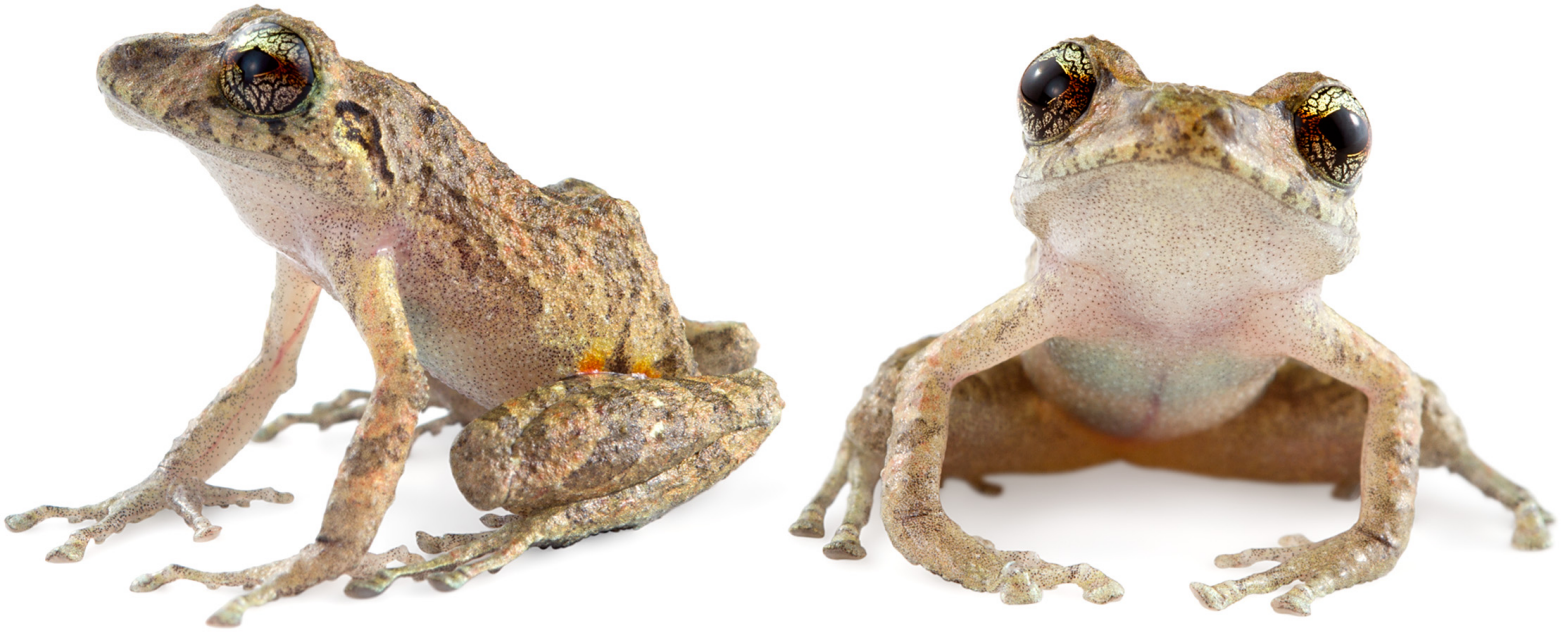
*Pristimantis buenaventura* in life. MZUTI 3480, SVL 19.2 mm, adult male, holotype.

MZUTI 3480, an adult male (Figs 19 and 20) obtained by Alejandro Arteaga, Lucas Bustamante, Alex Pyron, Jorge Castillo, Daniel Mideros, Diana Troya and Rita Hidalgo on December 24, 2013, at California (03.36799 S 79.73551 W; 225 m), El Oro province, Ecuador.

#### Paratopotypes

**Fig 21:**
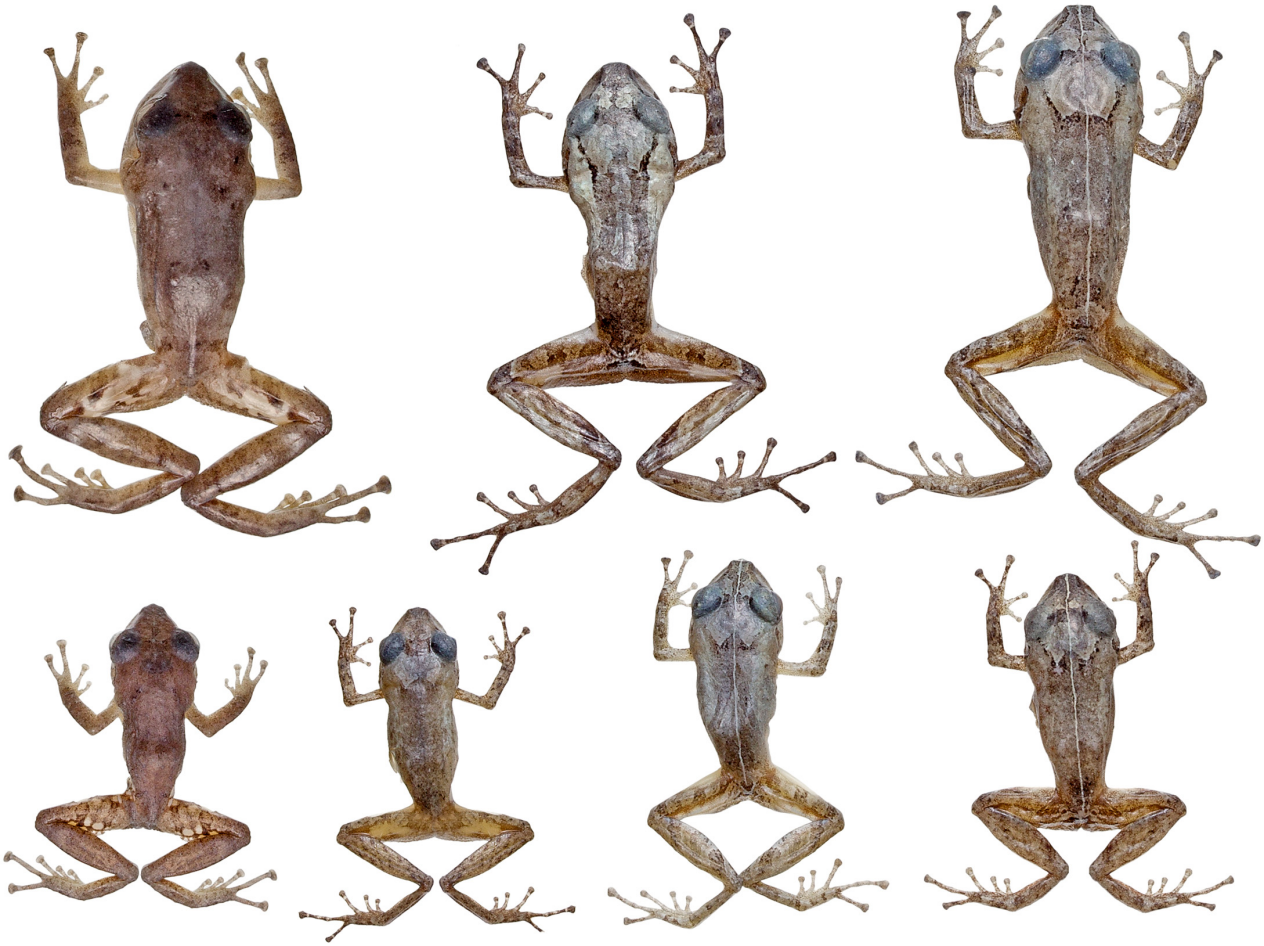
Color variation in the type series of *Pristimantis buenaventura*. From left to right, these are: MZUTI 3356, MECN 11337, 11338, MZUTI 3480, MECN 11333, 11339, 11335. Females are shown in the first row.

Alejandro Arteaga, Lucas Bustamante, Alex Pyron, Jorge Castillo, Daniel Mideros, Diana Troya and Rita Hidalgo collected two adult males (MZUTI 3476, 3432) (Figs 16 and 21) in December 2013.

#### Paratypes

On November 2013, Lucas Bustamante and Alejandro Arteaga collected one adult male (MZUTI 3270) and one adult female (MZUTI 3356) at the vicinities of Reserva Buenaventura (03.66014 S 79.77928 W; 565 m), El Oro province, Ecuador. On February 2014, Juan Carlos Sánchez, Karem López, Luis Oyagata and Paúl Guerrero collected two adult females (MECN 11337, 11338) (Fig 21) and seven adult males (MECN 11331–11336, 11339) at Cascadas de Manuel, El Oro province, Ecuador (03.21341 S 79.72598 W; 806 m). On November 2013, Juan Carlos Sánchez, Paúl Meza, Edison Rea and Karem López collected three adult males (MECN 10888, 10897, 10922) at Marcabelí, El Oro province, Ecuador (03.73918 S 79.89429 W; 662 m). On April 2012, Mario Yánez-Muñoz, Miguel Alcoser, María Perez and Marco Reyes collected two adult males (MECN 9472 and 9467) at Reserva Buenaventura, El Oro province, Ecuador (03.65858 S 79.77078 W; 534 m). On September 2012, María Perez, Karem López and David Brito collected one adult male (MECN 10778) on Playa Limón, El Oro province, Ecuador (03.50096 S 79.74701 W; 816 m).

#### Diagnosis

The new species is placed in the genus *Pristimatis*, as diagnosed by Hedges *et al*. [51], based on phylogenetic evidence (Fig 6). It is included in a phenetic assemblage of Trans-Andean *Pristimantis* characterized by their yellow to orange pigmentation in the hidden surfaces of the hind limbs. *Pristimantis buenaventura* is diagnosed by having the following features. (1) Skin texture of dorsum and flanks finely shagreen with low, scattered tubercles and no dorsolateral folds. Venter areolate (Fig 19). (2) Tympanic membrane absent, but tympanic annulus evident, 31–43% diameter of eye, with upper rim obscured by supratympanic fold. (3) Snout length 17–21% of SVL, rounded in dorsal view and rounded in profile. (4) Upper eyelid bearing low, non-conical tubercles. In females, upper eyelid width 80–85% of interorbital distance (63–109% of that distance in males). Cranial crests absent. (5) Dentigerous processes of vomers well developed, oblique in outline, each bearing 4–5 teeth. (6) Males have a small subgular vocal sac and vocal slits, but no nuptial pads. (7) Finger I shorter than II. Discs on fingers expanded, rounded to elliptical and sometimes spadate, except for Finger I that is only barely expanded. (8) Fingers bear narrow lateral fringes. Outer palmar tubercle distally divided, and supernumerary tubercles low and indistinct. (9) Ulnar tubercles low. (10) Low tubercles present on heel but not on tarsus. (11) Toes bear narrow fringes but no webbing. Toe V slightly longer than Toe III. Toe discs are expanded, rounded to elliptical and, in some individuals, spadate. (12) Inner metatarsal tubercle elliptical, about four times the size of the outer tubercle, which is rounded and low. Supernumerary plantar tubercles round and low. (13) In ethanol (Figs 19 and 21), the dorsum of *P*. *buenaventura* varies from pale reddish brown to grayish brown (light brown in life; Figs 16 and 20) with dark, faint irregular markings. Dark supratympanic stripe and 1–3 faint supralabial bars present. Hidden surfaces of hind limbs cream to brown and enclosing irregular smoky-white blotches (orange-red in life). Background color of throat, belly and ventrolateral surfaces dingy white, with different levels of fine brown mottling. In life, iris khaki with black reticulations and crossed by a coppery median streak. (14) SVL in females 25.2–29.9 mm (mean = 27.1 ± 2.5; n = 3). In males 18.7–21.9 mm (mean = 20.1 ± 1.1; n = 8).

#### Genetic characters

This species can be distinguished from its closest morphological relative, *Pristimantis walkeri*, by a sequence variation of 13.8–14.0% in a 731-bp long fragment of the mitochondrial 12S gene. Sampled specimens (MZUTI 3270, 3356, 3480) of *P*. *buenaventura* form a cluster genetically divergent from all other members of the *P*. *walkeri* species complex (Fig 6).

#### Similar species

*Pristimantis buenaventura* differs from other Trans-Andean yellow to orange groined members of the *P*. *unistrigatus* group and other morphologically similar congeners (see Table 4) by having orange-red blotches on a brown background in the hidden surfaces of the hind limbs, low heel tubercles, and a no oblique-lateral stripe (Fig 16). The most similar rainfrog is *P*. *walkeri*, but this species has bright yellow blotches on the groin (Table 4) and lacks scattered tubercles on dorsal surfaces.

#### Description of the holotype

Adult male (MZUTI 3480; Figs 19 and 20). Head slightly wider than body and slightly longer than wide. Upper eyelid bearing several low tubercles both in life and preserved. Head width 39% of SVL. Head length 42% of SVL. Snout 20% of SVL, rounded in dorsal view (Fig 19) and rounded in profile. Tongue longer than wide, with posterior half notched and not adherent to floor of mouth. Eye diameter slightly larger than eye–nostril distance. Nostrils not protuberant and directed anterolaterally. Canthus rostralis and loreal region weakly concave in profile. Upper eyelid width 79% of interorbital distance. Cranial crests and tympanic membrane absent, but tympanic annulus distinct and round. Two postrictal tubercles present. Choanae round and not concealed by palatal shelf of maxillary. Vomerine odontophores oblique in outline and about 30% shorter than diameter of choana, located posteromedial to choanae and separated medially by distance greater than width of odontophore. Each bearing 4 teeth. Vocal slits and median, subgular vocal sac present. Skin on dorsal and lateral surfaces finely shagreen (Fig 19). Skin on ventral surfaces areolate. Discoidal and thoracic folds absent (Fig 19). Cloacal sheath absent, but cloacal region bordered ventrally by low tubercles. Ulnar tubercles present, but no ulnar fold. Outer palmar tubercle present and distally divided. Palmar subarticular and supernumaray tubercles round in section. Fingers bearing narrow lateral fringes. Finger I shorter than Finger II. Disc of Finger I barely expanded. All other discs expanded, twice the width of proximal phalanx, and rounded to elliptical. Ventral pads well defined by circumferential grooves. No nuptial pads present. Tibia length 52% of SVL, and foot length 43% of SVL. Inner metatarsal tubercle about four times the size of the outer, rounded tubercle. Plantar subarticular and supernumerary tubercles round in section. Toes bear narrow lateral fringes, but no webbing. Discs of Toe I barely expanded. All other toe discs expanded and rounded to slightly elliptical. Toes have ventral pads well defined by circumferential grooves. Relative length of toes is: I < II < III < V < IV. Toe V slightly longer than Toe III.

#### Measurements of holotype (in mm)

SVL 19.2; tibia length 10.0; foot length 8.2; head length 8.0; head width 7.5; eye diameter 2.6; interorbital distance 2.6; upper eyelid width 2.0; internarial distance 1.7; eye-nostril distance 2.3.

#### Coloration of holotype in preservative

Dorsal surfaces rosy brown with faint irregular darker markings (Fig 1). Dorsal surfaces of hind limbs irregularly banded with dark pigment. Three faint supralabial bars accompany a blackish supratympanic stripe. Background color of the ventral surfaces dingy white with a gentle brown mottling overall.

#### Coloration of holotype in life

Based on field notes by Alejandro Arteaga. Upper and lateral surfaces orangish brown with irregular areas of darker or lighter color, and faint transverse bars on the limbs. Background color of ventral surfaces dingy white with a gentle brown mottling overall. Groin and hidden surfaces of hind limbs dark brown with irregular orange-red spots and blocthes. Iris goldenrod with black reticulations and crossed by a coppery median streak.

#### Variation

Morphological variation is presented in Tables 7 and 8. Females are larger than males, but overall less patterned. Most specimens in the series have rounded to elliptical digits, but MECN 11333–4, 11337–8 and MZUTI 3432 have some of their digital disks spadate (Fig 21). A pale paravertebral line is present in MECN 11335, 11338–9. A pale occipital patch is present in MECN 11332, 11334, 11337 and 11338. Most specimens in the series have low to indistinct tubercles scattered all over the dorsal surfaces. In MZUTI 3432, these tubercles are more numerous and elevated (Fig 21).

**Table 7:** Measurements (in mm) of adults of *Pristimantis buenaventura*.

	Females (N = 3)	Males (N = 8)
SVL	25.2–29.9 (27.1 ± 2.5)	18.7–21.9 (20.1 ± 1.1)
Tibia length	13.2–13.6 (13.4 ± 0.2)	9.9–11.8 (10.4 ± 0.6)
Foot length	10.5–11.2 (10.9 ± 0.3)	7.8–9.9 (8.7 ± 0.7)
Head length	8.8–11.9 (10.3 ± 1.6)	7.4–8.7 (8.0 ± 0.5)
Head width	9.7–11.2 (10.2 ± 0.8)	7.3–8.3 (7.7 ± 0.4)
Interorbital distance	2.8–3.6 (3.2 ± 0.4)	2.2–2.6 (2.4 ± 0.2)
Upper eyelid width	2.3–2.9 (2.6 ± 0.3)	1.4–2.4 (2.0 ± 0.3)
Radioulna length	5.7–6.5 (6.2 ± 0.5)	4.4–5.6 (4.9 ± 0.4)
Eye-to-nostril distance	2.9–3.8 (3.3 ± 0.4)	2.2–2.9 (2.5 ± 0.2)
Snout-to-eye distance	4.7–5.3 (5.0 ± 0.3)	3.3–4.2 (3.8 ± 0.3)
Eye diameter	3.2–3.8 (3.4 ± 0.3)	2.6–3.1 (2.8 ± 0.2)
Tympanum diameter	1.0–1.5 (1.3 ± 0.3)	0.8–1.1 (1.0 ± 0.1)
Hand length	6.2–6.8 (6.6 ± 0.4)	5.2–6.0 (5.4 ± 0.3)
Finger I length	4.1–4.2 (4.2 ± 0.0)	2.8–3.5 (3.1 ± 0.2)

**Table 8:** Ranges of morphological proportions (in percentages) of adults of *Pristimantis buenaventura*.

	Females (N = 3)	Males (N = 8)
Tibia length/SVL	44.3–52.4	45.8–55.1
Foot length/SVL	36.9–42.5	39.5–46.3
Foot length/tibia length	79.8–83.3	79.2–88.8
Head width/SVL	37.3–38.4	36.5–39.9
Head length/SVL	34.9–39.7	36.8–42.4
Head length/head width	90.9–106.5	95.8–107.8
Eye-to-nostril distance/eye diameter	87.7–101.3	80.7–92.6
Upper eyelid width/IOD	80.1–84.5	62.8–109.3
Radioulna length/SVL	21.8–24.6	23.1–25.8
Hand length/radioulna length	95.2–119.0	94.1–119.9
Finger I length/hand length	61.2–67.3	53.0–59.5

#### Natural history

Specimens of *Pristimantis buenaventura* have been found active by night on vegetation 60–160 cm above the ground in secondary semideciduous foothill forest and evergreen lower-montane forests (Figs 1 and 15). *P*. *buenaventura* inhabits hilly forests where the canopy cover is 75–90%. Most individuals have been found far from water and active on rainless nights. At Reserva Buenaventura, *P*. *buenaventura* is syntopic with *P*. *achatinus*, *P*. *subsigillatus* and two undescribed species of *Pristimantis*.

#### Distribution

225–1070 m. *Pristimantis buenaventura* is endemic to the foothill forests of southwestern Ecuador (Fig 15). Besides the six localities of the type series, the species has been previously reported in two localities under the name *P*. *walkeri* [22], all south of the Río Jubones (Fig 1) (S4 Table).

#### Etymology

The specific epithet *buenaventura* refers to Reserva Buenaventura, a protected forest where the new species is known to occur. The epithet is a noun in apposition.

#### Conservation status

We consider *Pristimantis buenaventura* to be Vulnerable following B1a IUCN criteria because its extent of occurrence is estimated to be 961 km^2^, its habitat is severely fragmented [112], and it is known to exist at no more than eight localities.

## Discussion

When analyzed together, our five mtDNA phylogenies and 14 species distribution models reveal a pattern of cladogenesis that is common for five pairs of codistributed sister-species pairs in northwestern Ecuador. The pattern can be described as a speciation event in which a widely distributed lowland (Chocoan) taxon, whose predicted area of suitable habitat overlaps mainly with evergreen lowland forest, is sister to a more restricted montane-forest vicariant, whose predicted area of suitable habitat overlaps mainly with evergreen lower-montane forest [42]. Although different models of diversification were not tested in this study, we speculate that a parapatric model is the most likely scenario because populations of the sister species are not separated by a geographical barrier, but by changes in vegetation zones and climatic regimes along the elevational gradient.

In this common phylogeographic pattern, the suitable habitat for the upland vicariant corresponds mainly (Table 3) with lower-montane forests in the area between the Río Mira and Toachi valleys, the area in Ecuador where these two vegetation zones are wider (Fig 1) and closer to the Equatorial line. Closer to the Equator, elevation gradients may have a stronger effect on the dispersal of organisms than a similar gradient on temperate regions [113], because species occupy more restricted elevational ranges and have narrower thermal tolerances [114, 115, 116, 117, 118, 119]. This greater climatic stratification is hypothesized to increase the likelihood of parapatric speciation along elevational gradients [15, 120], and may explain why our sampled Chocoan lineages have upland vicariants in the montane forests closer to the Equatorial line.

Besides the deep and geographically structured split between the sister taxa included in this study, our results also show geographically structured mitochondrial subdivisions within species. In Ecuador, we can identify at least two barriers where our sampled taxa share a major break in genetic composition. (1) The Río Guayllabamba (Fig 1) is likely responsible for the majority of the genetic heterogeneity observed in *Pristimantis crenunguis* (Fig 4), *P*. *mindo* (Fig 5), and *P*. *luteolateralis* (Fig 6). The Río Guayllabamba has also been recognized as a genetic boundary in other cloudforest taxa [24]. (2) The Río Esmeraldas (Fig 1) may also be responsible for some of the genetic heterogeneity observed in *P*. *labiosus*, and is the main barrier separating populations of *P*. *nietoi* and *P*. *walkeri*. Two other rivers (Mira, Toachi) have presumably acted as effective barriers of dispersal for *Alopoglossus viridiceps*, *P*. *crenunguis*, *P*. *mindo* and *P*. *luteolateralis*, since none of these species has been found either north of Río Mira or south of Río Toachi. Likewise, the dry valley of the Río Chimbo (Fig 1) may explain part of the observed genetic diversity within sampled populations of *A*. *festae* (Fig 3), and may be the barrier that has most likely prevented *Bothrops osbornei* from colonizing cloudforests South of its know distribution [89].

In one species, *Pristimantis labiosus*, the presence of a distinct genetic lineage formed by MECN 9527, 9528 and MZUTI 3018 (Fig 4) suggests that not all mitochondrial subdivisions within our sampled species are geographically structured. This lineage is sister to all other sampled populations of *P*. *labiosus* (Fig 4), and might represent a distinct species separated not by a geographic barrier (both lineagues of *P*. *labiosus* are sympatric at Reserva Itapoa), but by niche partitioning, differential selection or secondary contact after allopatric speciation.

The shared phylogenetic breaks at the species and population level across the vegetation zones and river valleys in northwestern Ecuador suggests that speciation due to shared responses to environmental features, probably coupled with fragmentation of the once countinuous lowlands [22, 28], may have driven an important part of the current observed diversity of this region. Our study suggests that widely distributed Chocoan taxa may generally experience their greatest opportunities for isolation and parapatric speciation across elevational gradients in the adjacent montane forests. Future research should estimate the history of colonization to pinpoint the timing and direction of elevational transitions and speciation events across a broad spectrum of taxa. This will allow us to determine if more lineages originate in the Andes over time and disperse infrequently to the lowlands [121], or if lowland lineages more frequently colonize and diversify in the cloudforests [122].

We suggest that our discovery of hidden species richness and their common patterns of speciation represent working hypotheses for other unstudied taxa or communities that range both in Chocoan lowlands and their adjacent Equatorial montane forests (e.g. *Anadia rhombifera*, *Tantilla melanocephala*, *Hyloscirtus alytolylax* [25], *Pristimantis ornatissimus* and *P*. *parvillus*). Many such groups may in fact be species complexes, with populations inhabiting the montane forests representing distinct evolutionary units that deserve full-species status.

## Supporting Information

S1 Fig Ecological niche models (ENM) for the sampled sister-species pairs, and new species described in this study. The Minimum Training Presence (MTP) threshold was used to validate the models; over-predicted areas east of the Andes are not shown.
(PDF)

S1 Table GenBank accession numbers for loci and terminals of taxa and outgroups sampled in this study. Specimens for which novel sequence data was produced in this study are marked with an asterisk (*).
(DOCX)

S2 Table List of PCR and sequencing primers and their respective PCR conditions used in this study. All PCR protocols included an initial 3-min step at 94°C and a final extension of 10 min at 72°C.
(DOCX)

S3 Table Additional specimens examined. (DOCX)

S4 Table Locality data for species included in this study. In general, localities are given as transcribed from the literature, museum records, Tropical Herping photographic database or HerpNET. Coordinates represent georeferencing attempts from gazetteers under standard guidelines, though some variation from the exact collecting locality will inevitably be present. Similarly, elevations are taken from Google Earth, and may not exactly match the elevations as originally reported.
(DOCX)
