# Supplementary figures and images for: Comparative Phylogeography Reveals Cryptic Diversity and Repeated Patterns of Cladogenesis for Amphibians and Reptiles in Northwestern Ecuador
Source: PLoS One. 2016 Apr 27;11(4):e0151746. doi: 10.1371/journal.pone.0151746 (PMC4847877; doi:10.1371/journal.pone.0151746)

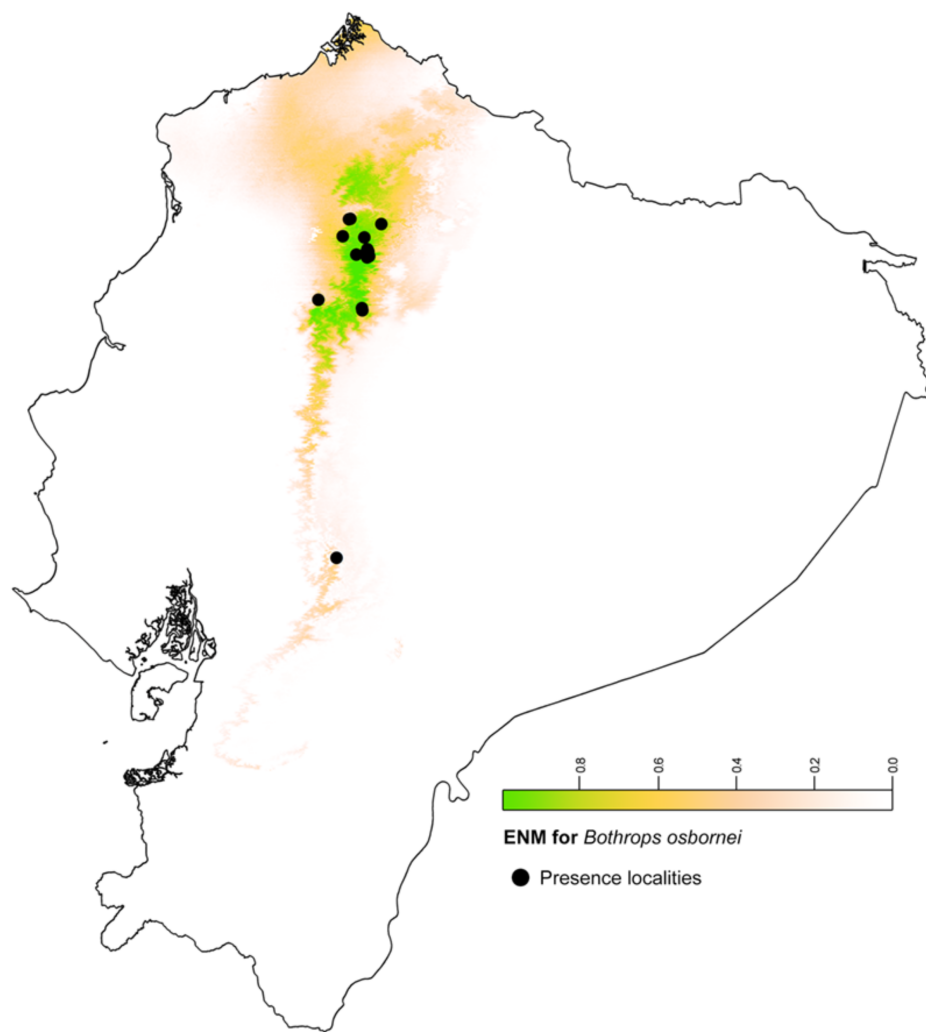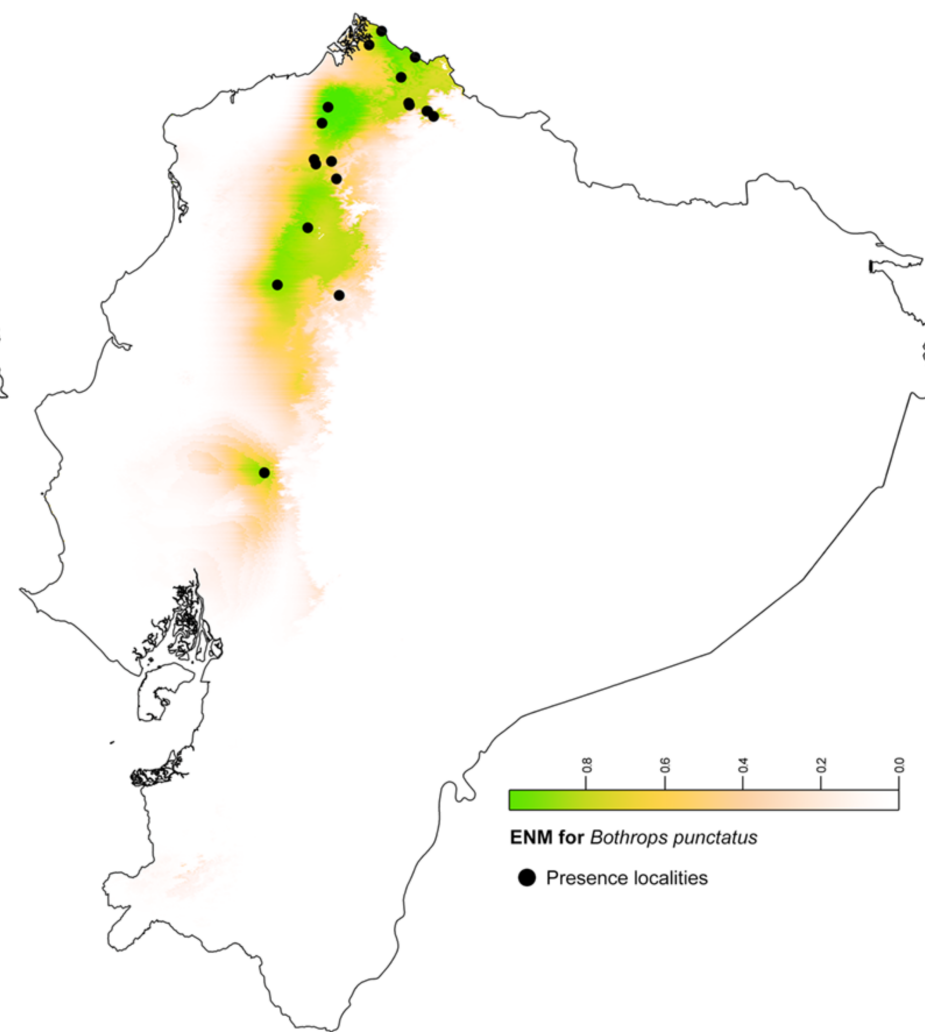

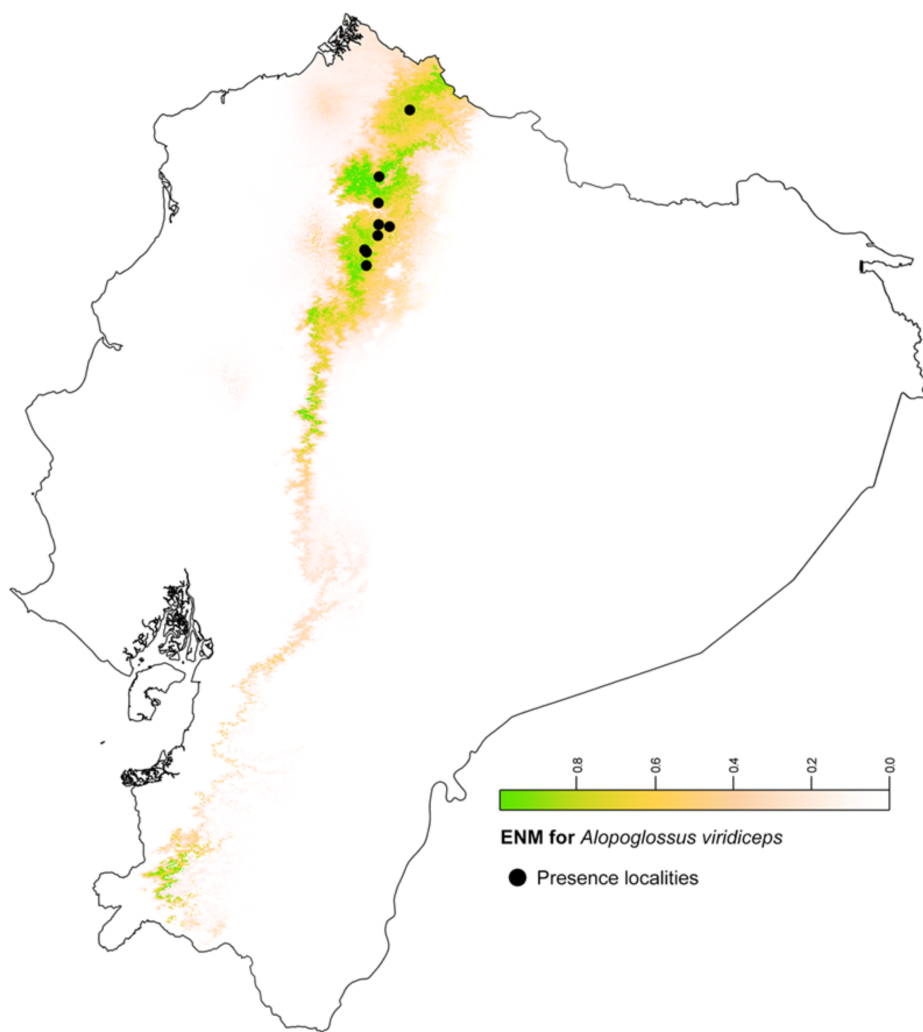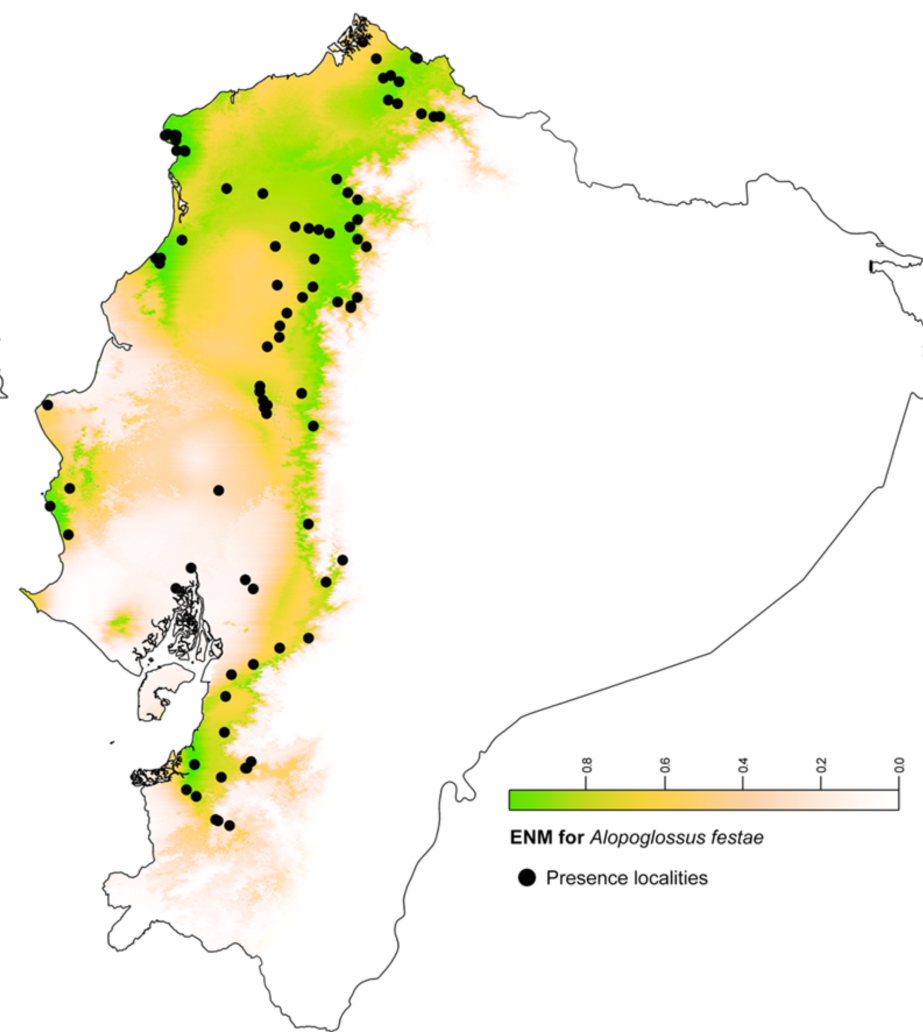

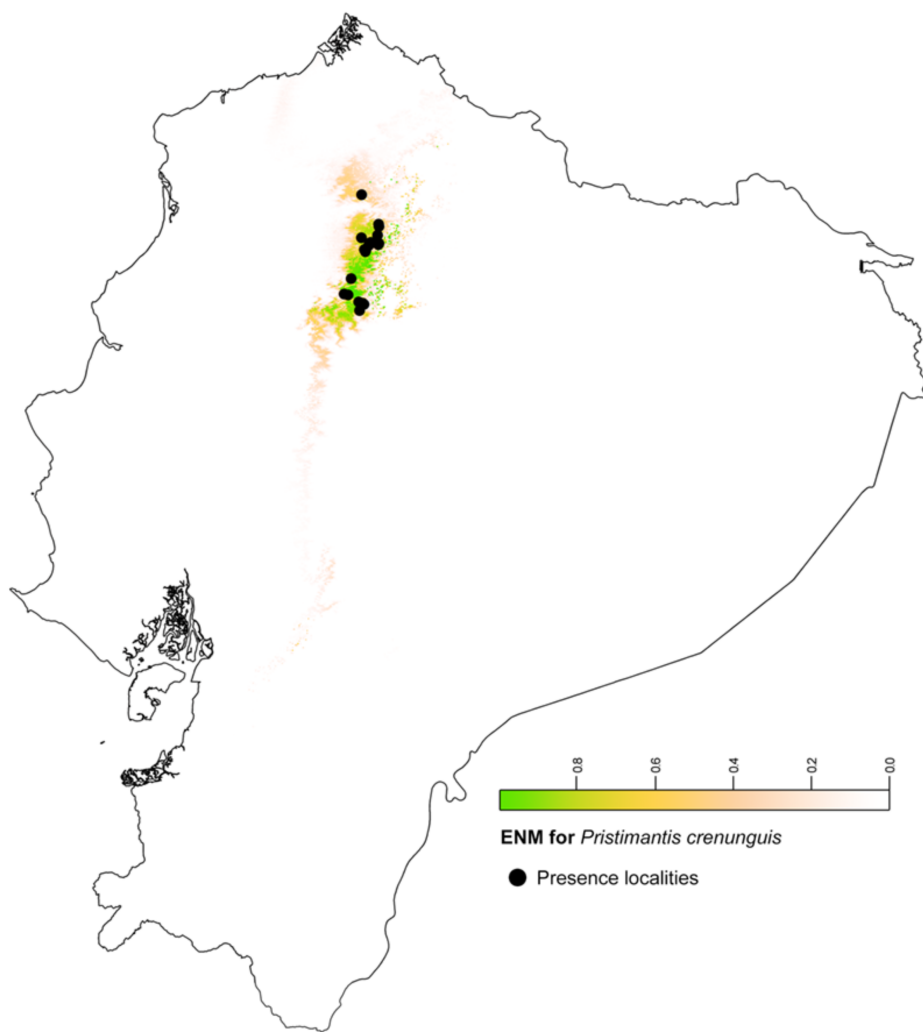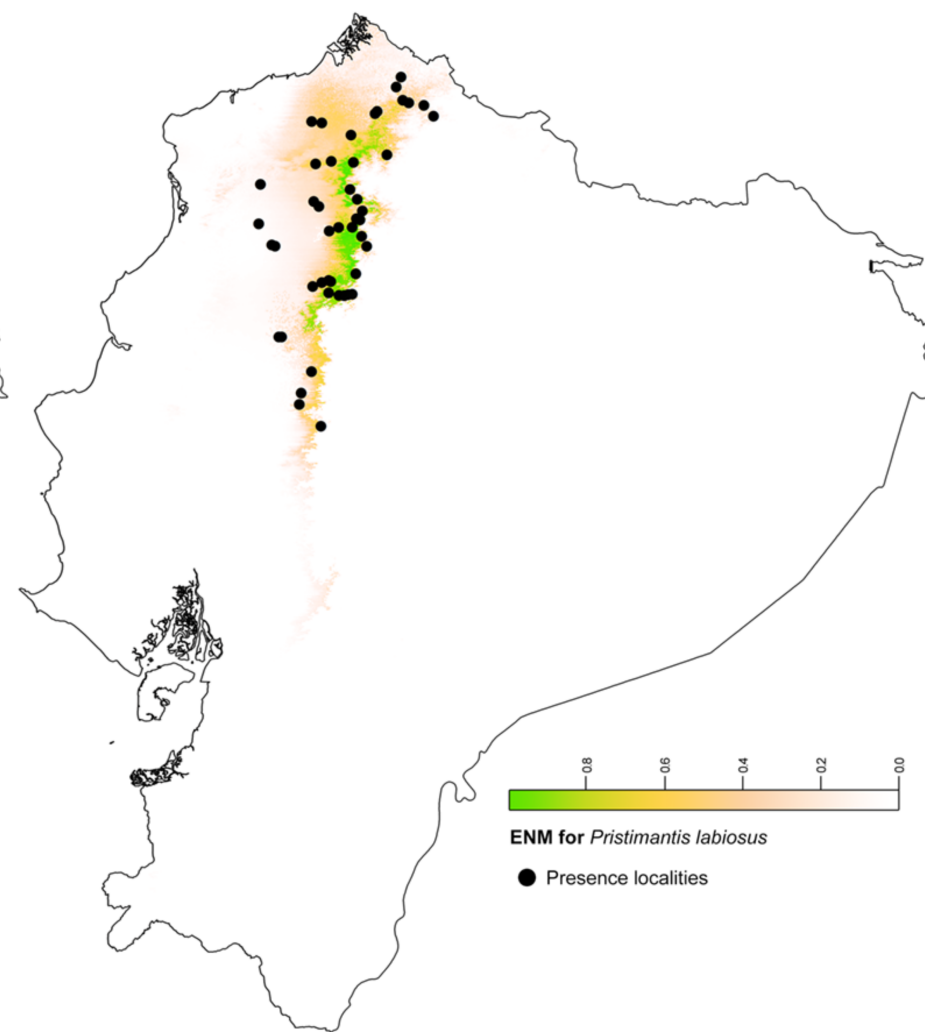

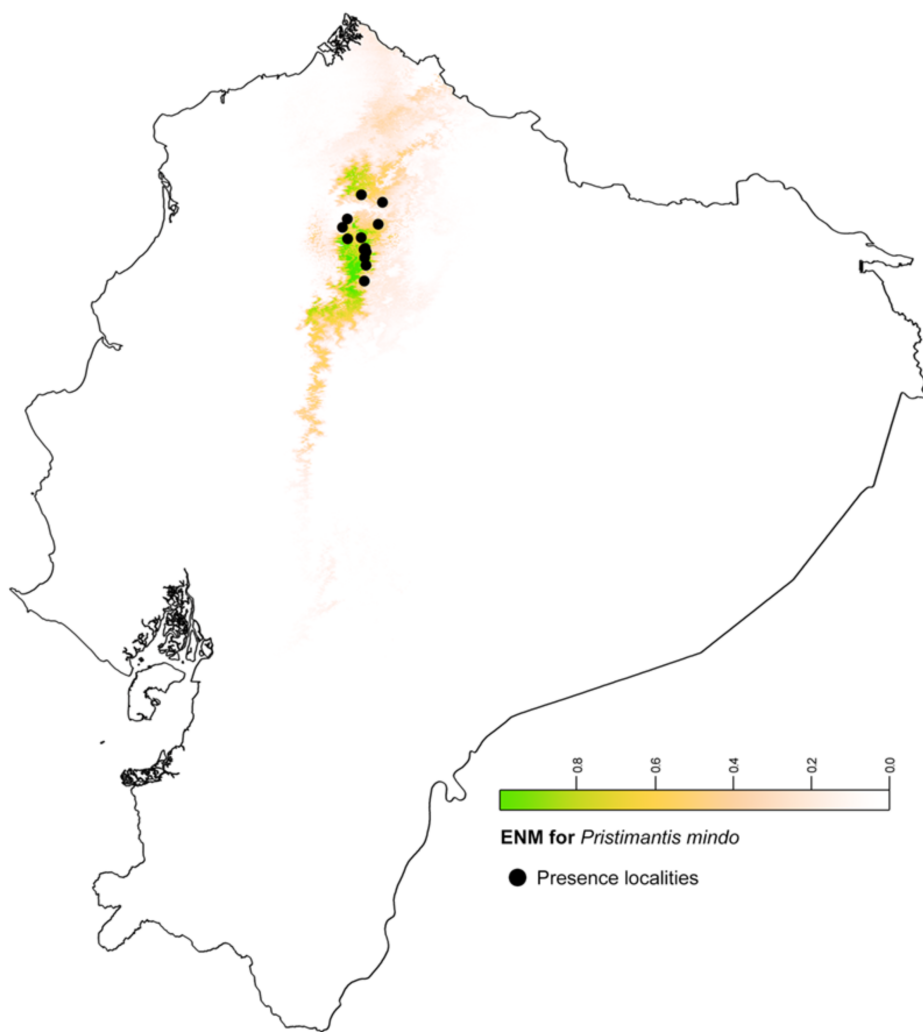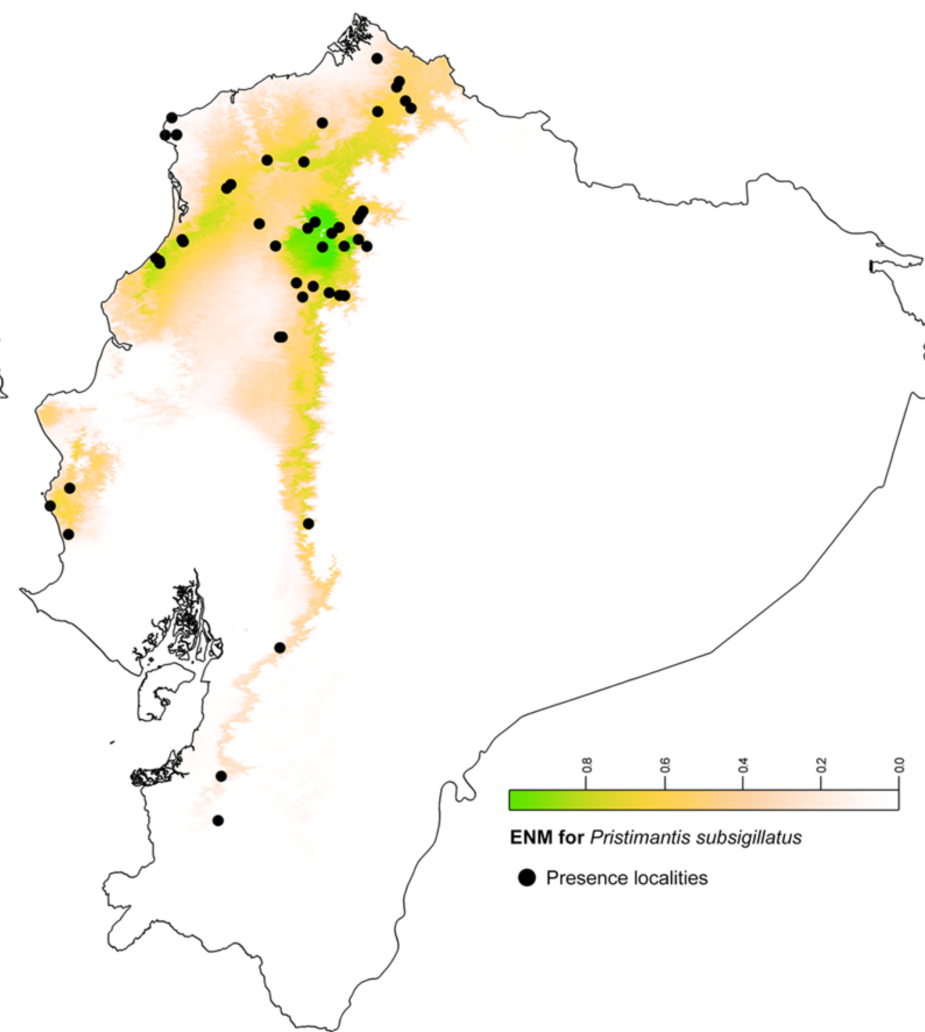

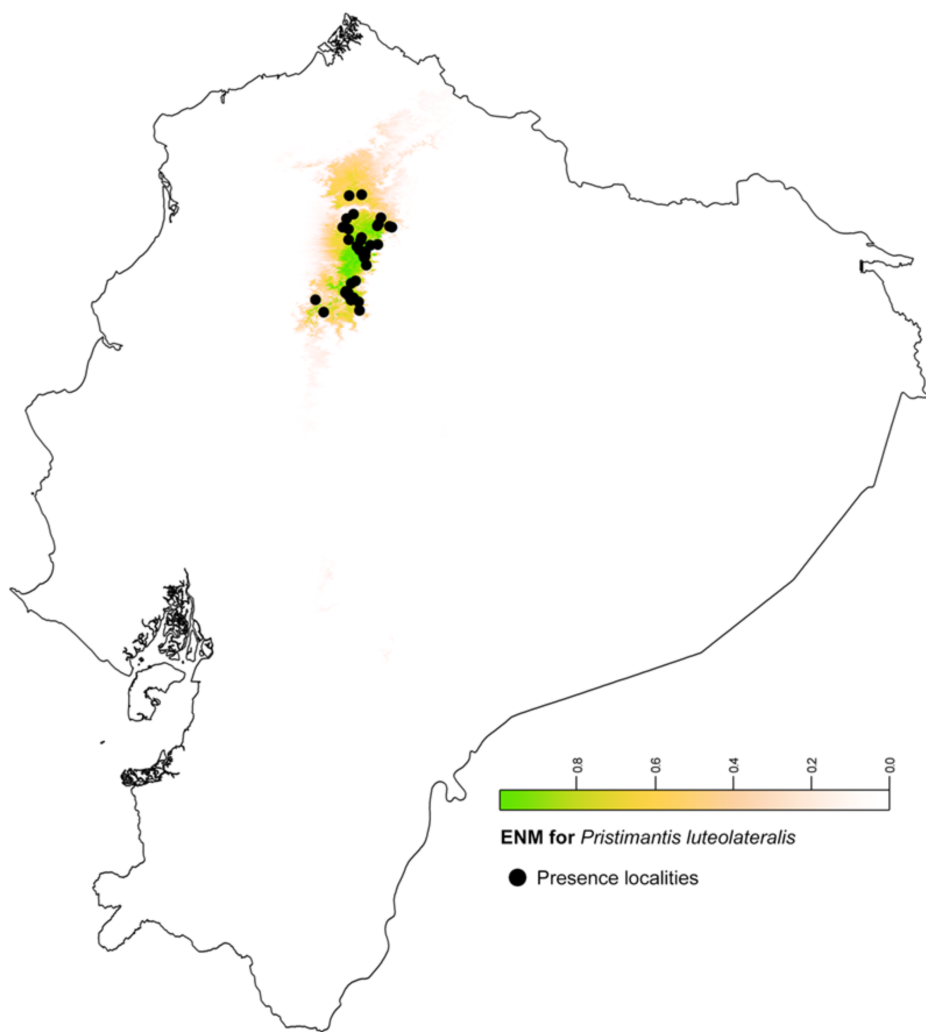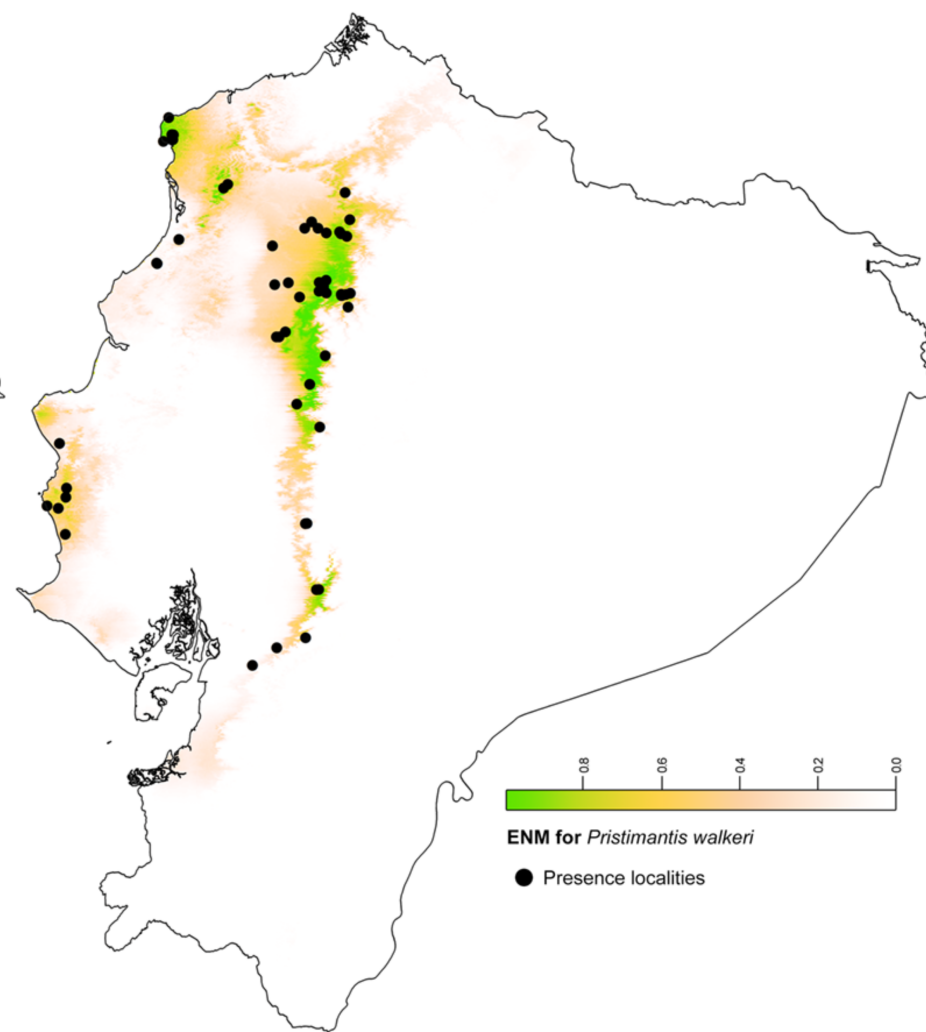

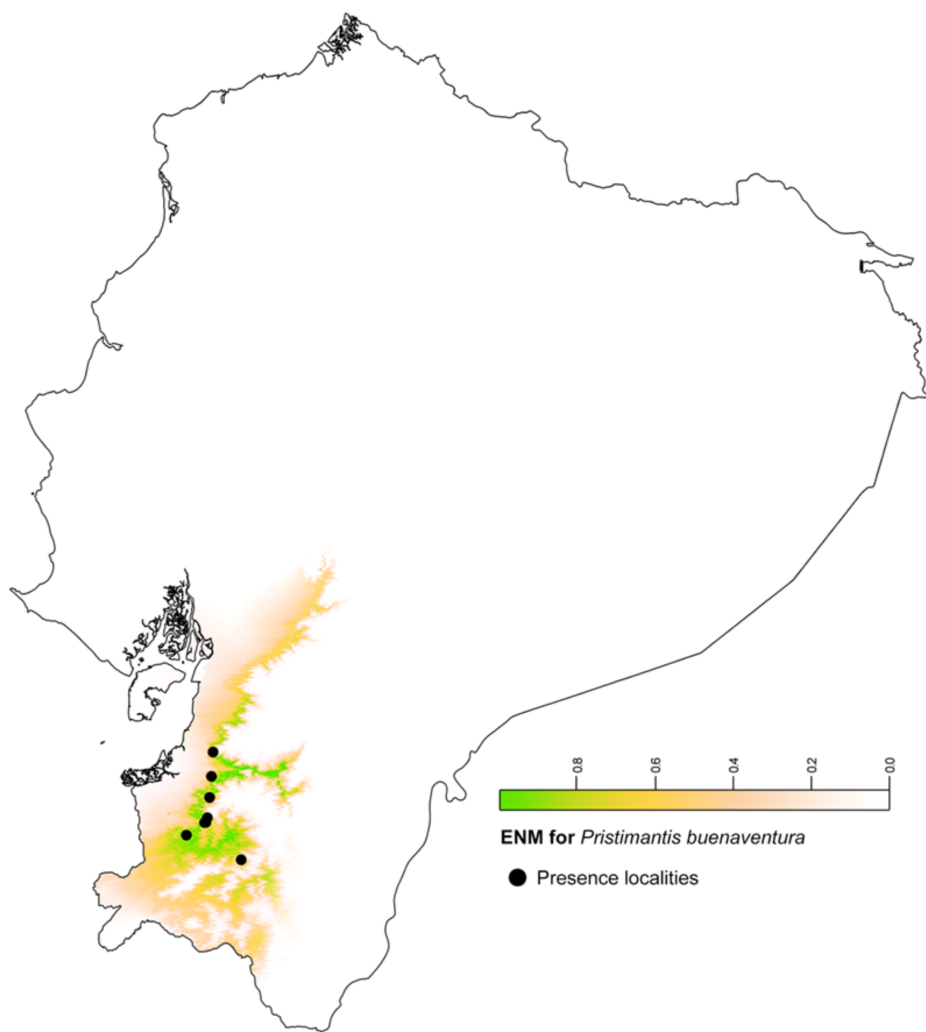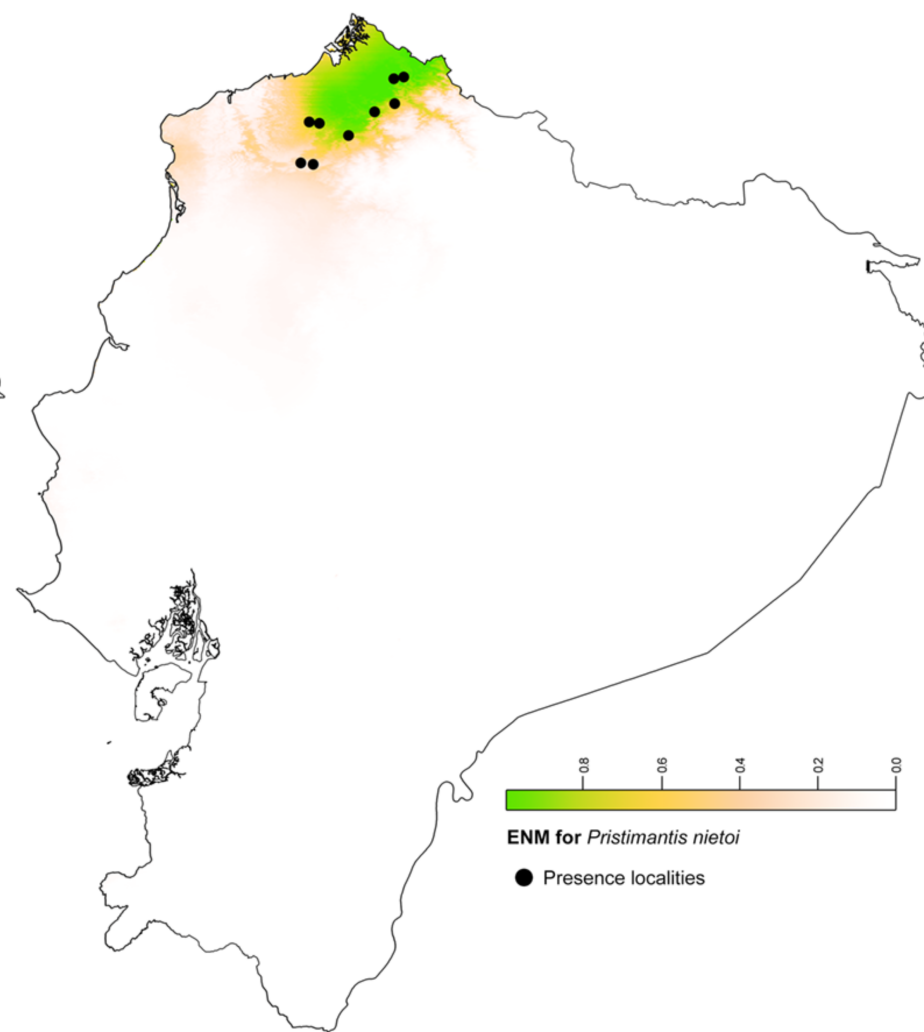

Supplement: S1 Fig — The Minimum Training Presence (MTP) threshold was used to validate the models; over-predicted areas east of the Andes are not shown. (PDF) [file pone.0151746.s001.pdf]
