# Supplementary material for: Comparative Phylogeography Reveals Cryptic Diversity and Repeated Patterns of Cladogenesis for Amphibians and Reptiles in Northwestern Ecuador
Source: PLoS One. 2016 Apr 27;11(4):e0151746. doi: 10.1371/journal.pone.0151746 (PMC4847877; doi:10.1371/journal.pone.0151746)
Supplement: S1 Table — Specimens for which novel sequence data was produced in this study are marked with an asterisk (*). (DOCX) [file pone.0151746.s002.docx]

| **Species** | **Voucher** | **12S** | **16S** | **cytb** | **ND4** |
| --- | --- | --- | --- | --- | --- |
| ***Craugastor longirostris*** | KU177803 | EF493395 | EF493395 | – | – |
| ***Pristimanits latidiscus*** | KU218016 | EF493698 | EF493698 | – | – |
| ***Pristimantis acerus*** | KU217786 | EF493678 | EF493678 | – | – |
| ***Pristimantis actites*** | KU217830 | EF493696 | EF493696 | – | – |
| ***Pristimantis acuminatus*** | QCAZ19664 | – | EU130579 | – | – |
| ***Pristimantis altae*** | AJC0398 | JN991496 | – | – | – |
| ***Pristimantis appendiculatus*** | KU177637 | EF493524 | EF493524 | – | – |
| ***Pristimantis bromeliaceus*** | KU291702 | EF493351 | EF493351 | – | – |
| ***Pristimantis buenaventura**** | MZUTI3270 | KU999240 | KU999169 | – | – |
| ***Pristimantis buenaventura**** | MZUTI3356 | KU999241 | KU999170 | – | – |
| ***Pristimantis buenaventura**** | MZUTI3480 | KU999242 | – | – | – |
| ***Pristimantis calcarulatus*** | KU177658 | EF493523 | EF493523 | – | – |
| ***Pristimantis caryophyllaceus*** | MVZ203810 | EU186686 | EU186686 | – | – |
| ***Pristimantis cerasinus*** | AJC1142 | JN991502 | JN991438 | – | – |
| ***Pristimantis chalceus*** | KU177638 | EF493675 | EF493675 | – | – |
| ***Pristimantis cremnobates*** | KU177252 | EF493528 | EF493528 | – | – |
| ***Pristimantis crenunguis*** | KU1777730 | EF493693 | EF493666 | – | – |
| ***Pristimantis crenunguis**** | MZUTI530 | KU999243 | KU999171 | – | – |
| ***Pristimantis crenunguis**** | MZUTI531 | KU999244 | KU999172 | – | – |
| ***Pristimantis crenunguis**** | MZUTI532 | KU999245 | KU999173 | – | – |
| ***Pristimantis crenunguis**** | MZUTI1398 | – | KU999174 | – | – |
| ***Pristimantis crenunguis**** | MZUTI1399 | – | KU999175 | – | – |
| ***Pristimantis crenunguis**** | MZUTI2987 | – | KU999176 | – | – |
| ***Pristimantis crenunguis**** | MZUTI3067 | – | KU999177 | – | – |
| ***Pristimantis crenunguis**** | MZUTI3068 | – | KU999178 | – | – |
| ***Pristimantis crenunguis**** | MZUTI3069 | KU999246 | KU999179 | – | – |
| ***Pristimantis crenunguis**** | MZUTI3292 | – | KU999180 | – | – |
| ***Pristimantis crenunguis**** | MZUTI3296 | – | KU999181 | – | – |
| ***Pristimantis crenunguis**** | MZUTI3304 | KU999247 | KU999182 | – | – |
| ***Pristimantis crucifer*** | KU177733 | EU186736 | EU186718 | – | – |
| ***Pristimantis dissimulatus*** | KU179090 | EF493522 | EF493522 | – | – |
| ***Pristimantis erythropleura*** | UVC15886 | – | JN371036 | – | – |
| ***Pristimantis galdi*** | QCAZ32368 | EU186670 | EU186670 | – | – |
| ***Pristimantis glandulosus*** | KU218002 | EF493676 | EF493676 | – | – |
| ***Pristimantis inusitatus*** | KU218015 | EF493677 | EF493677 | – | – |
| ***Pristimantis labiosus**** | MECN9527 | KU999248 | KU999183 | – | – |
| ***Pristimantis labiosus**** | MECN9528 | KU999249 | KU999184 | – | – |
| ***Pristimantis labiosus**** | MZUTI573 | – | KU999185 | – | – |
| ***Pristimantis labiosus**** | MZUTI574 | – | KU999186 | – | – |
| ***Pristimantis labiosus**** | MZUTI577 | – | KU999187 | – | – |
| ***Pristimantis labiosus**** | MZUTI589 | KU999250 | KU999188 | – | – |
| ***Pristimantis labiosus**** | MZUTI594 | – | KU999189 | – | – |
| ***Pristimantis labiosus**** | MZUTI1759 | KU999251 | KU999190 | – | – |
| ***Pristimantis labiosus**** | MZUTI3000 | – | KU999191 | – | – |
| ***Pristimantis labiosus**** | MZUTI3018 | KU999252 | KU999192 | – | – |
| ***Pristimantis labiosus**** | MZUTI3051 | KU999253 | KU999193 | – | – |
| ***Pristimantis labiosus**** | MZUTI3078 | – | KU999194 | – | – |
| ***Pristimantis labiosus**** | MZUTI3079 | – | KU999195 | – | – |
| ***Pristimantis labiosus**** | MZUTI3080 | KU999254 | KU999196 | – | – |
| ***Pristimantis labiosus*** | QCAZ19771 | EF493694 | EF493694 | – | – |
| ***Pristimantis lanthanites*** | KU222001 | EF493695 | EF493695 | – | – |
| ***Pristimantis latidiscus**** | MZUTI2992 | – | KU999197 | – | – |
| ***Pristimantis luteolateralis*** | KU177807 | EF493517 | EF493517 | – | – |
| ***Pristimantis luteolateralis**** | MZUTI327 | KU999255 | – | – | – |
| ***Pristimantis luteolateralis**** | MZUTI328 | KU999256 | – | – | – |
| ***Pristimantis luteolateralis**** | MZUTI329 | KU999257 | – | – | – |
| ***Pristimantis luteolateralis**** | MZUTI330 | KU999258 | – | – | – |
| ***Pristimantis luteolateralis**** | MZUTI528 | KU999259 | – | – | – |
| ***Pristimantis luteolateralis**** | MZUTI529 | KU999260 | – | – | – |
| ***Pristimantis luteolateralis**** | MZUTI654 | KU999261 | KU999198 | – | – |
| ***Pristimantis luteolateralis**** | MZUTI655 | KU999262 | KU999199 | – | – |
| ***Pristimantis luteolateralis**** | MZUTI656 | KU999263 | KU999200 | – | – |
| ***Pristimantis luteolateralis**** | MZUTI657 | KU999264 | KU999201 | – | – |
| ***Pristimantis luteolateralis**** | MZUTI658 | – | KU999202 | – | – |
| ***Pristimantis luteolateralis**** | MZUTI659 | KU999265 | - | - | - |
| ***Pristimantis luteolateralis**** | MZUTI660 | KU999266 | KU999203 | – | – |
| ***Pristimantis luteolateralis**** | MZUTI661 | KU999267 | – | – | – |
| ***Pristimantis luteolateralis**** | MZUTI662 | KU999268 | – | – | – |
| ***Pristimantis luteolateralis**** | MZUTI663 | KU999269 | KU999204 | – | – |
| ***Pristimantis luteolateralis**** | MZUTI665 | – | – | – | – |
| ***Pristimantis luteolateralis**** | MZUTI703 | KU999270 | – | – | – |
| ***Pristimantis luteolateralis**** | MZUTI1404 | KU999271 | KU999205 | – | – |
| ***Pristimantis luteolateralis**** | MZUTI1405 | KU999272 | – | – | – |
| ***Pristimantis luteolateralis**** | MZUTI1406 | KU999273 | – | – | – |
| ***Pristimantis luteolateralis**** | MZUTI1734 | – | KU999206 | – | – |
| ***Pristimantis luteolateralis**** | MZUTI1742 | KU999274 | – | – | – |
| ***Pristimantis luteolateralis**** | MZUTI2110 | KU999275 | – | – | – |
| ***Pristimantis luteolateralis**** | MZUTI2115 | KU999276 | – | – | – |
| ***Pristimantis luteolateralis**** | MZUTI2196 | KU999277 | KU999207 | – | – |
| ***Pristimantis luteolateralis**** | MZUTI2988 | KU999278 | – | – | – |
| ***Pristimantis luteolateralis**** | MZUTI2989 | KU999279 | – | – | – |
| ***Pristimantis luteolateralis**** | MZUTI2990 | KU999280 | – | – | – |
| ***Pristimantis luteolateralis**** | MZUTI3092 | KU999281 | – | – | – |
| ***Pristimantis luteolateralis**** | MZUTI3093 | KU999282 | – | – | – |
| ***Pristimantis luteolateralis**** | MZUTI3182 | KU999283 | KU999208 | – | – |
| ***Pristimantis mindo*** | MZUTI1381 | – | KF801583 | – | – |
| ***Pristimantis mindo*** | MZUTI1382 | – | KF801584 | – | – |
| ***Pristimantis mindo**** | MZUTI1383 | – | KU999209 | – | – |
| ***Pristimantis mindo*** | MZUTI1755 | – | KF801582 | – | – |
| ***Pristimantis mindo*** | MZUTI1756 | – | KF801581 | – | – |
| ***Pristimantis mindo**** | MZUTI2109 | – | KU999210 | – | – |
| ***Pristimantis mindo**** | MZUTI2284 | – | KU999211 | – | – |
| ***Pristimantis moro*** | AJC1753 | JN991519 | JN991453 | – | – |
| ***Pristimantis museosus*** | KRL0739 | – | FJ784354 | – | – |
| ***Pristimantis nietoi**** | MZUTI3001 | KU999284 | KU999212 | – | – |
| ***Pristimantis nietoi**** | MZUTI3049 | KU999285 | KU999213 | – | – |
| ***Pristimantis nietoi**** | MZUTI3050 | KU999286 | KU999214 | – | – |
| ***Pristimantis nyctophylax*** | KU177812 | EF493526 | EF493526 | – | – |
| ***Pristimantis orcesi*** | KU218021 | EF493679 | EF493679 | – | – |
| ***Pristimantis paisa*** | AJC1344 | JN991524 | JN991459 | – | – |
| ***Pristimantis pardalis*** | CH6284 | JN991525 | JN991460 | – | – |
| ***Pristimantis parvillus*** | KU177821 | EF493352 | EF493352 | – | – |
| ***Pristimantis parvillus**** | MZUTI2121 | KU999288 | KU999216 | – | – |
| ***Pristimantis parvillus**** | MZUTI483 | KU999287 | KU999215 | – | – |
| ***Pristimantis pirrensis*** | AJC0594 | JN991528 | JN991462 | – | – |
| ***Pristimantis pycnodermis*** | KU218028 | EF493680 | EF493680 | – | – |
| ***Pristimantis ridens*** | AMNHA124551 | EF493355 | EF493355 | – | – |
| ***Pristimantis schultei*** | KU212220 | EF493681 | EF493681 | – | – |
| ***Pristimantis subsigillatus*** | MECN10117 | – | KF801580 | – | – |
| ***Pristimantis subsigillatus**** | MZUTI1999 | – | KU999217 | – | – |
| ***Pristimantis subsigillatus**** | MZUTI2228 | – | KU999218 | – | – |
| ***Pristimantis subsigillatus**** | MZUTI2243 | – | KU999219 | – | – |
| ***Pristimantis subsigillatus**** | MZUTI2653 | – | KU999220 | – | – |
| ***Pristimantis subsigillatus**** | MZUTI2995 | – | KU999221 | – | – |
| ***Pristimantis subsigillatus**** | MZUTI2996 | – | KU999222 | – | – |
| ***Pristimantis subsigillatus**** | MZUTI2997 | – | KU999223 | – | – |
| ***Pristimantis subsigillatus**** | MZUTI3087 | – | KU999224 | – | – |
| ***Pristimantis subsigillatus**** | MZUTI3088 | – | KU999225 | – | – |
| ***Pristimantis subsigillatus**** | MZUTI3196 | – | KU999226 | – | – |
| ***Pristimantis subsigillatus**** | MZUTI3198 | – | KU999227 | – | – |
| ***Pristimantis subsigillatus**** | MZUTI3433 | – | KU999228 | – | – |
| ***Pristimantis unistrigatus*** | KU218057 | EF493387 | EF493387 | – | – |
| ***Pristimantis viejas*** | EMM250 | JN991546 | JN991476 | – | – |
| ***Pristimantis w-nigrum*** | WED53045 | AY326004 | AY326004 | – | – |
| ***Pristimantis walkeri*** | KU218116 | EF493518 | EF493518 | – | – |
| ***Pristimantis walkeri**** | MZUTI1768 | KU999289 | – | – | – |
| ***Pristimantis walkeri**** | MZUTI1770 | KU999290 | – | – | – |
| ***Pristimantis walkeri**** | MZUTI2993 | KU999291 | KU999229 | – | – |
| ***Pristimantis walkeri**** | MZUTI3183 | KU999292 | KU999230 | – | – |
| ***Alopoglossus angulatus*** | LG1026 | – | AF420744 | – | AF420909 |
| ***Alopoglossus angulatus*** | QCAZ8915 | – | – | – | KJ705317 |
| ***Alopoglossus atriventris*** | LSUMZH13856 | – | AF420746 | – | AF420908 |
| ***Alopoglossus atriventris*** | QCAZ5622 | – | – | – | KJ705319 |
| ***Alopoglossus buckleyi**** | MZUTI4008 | – | KU999143 | – | KU999132 |
| ***Alopoglossus buckleyi**** | MZUTI4007 | – | KU999142 | – | KU999131 |
| ***Alopoglossus buckleyi*** | QCAZ9961 | – | – | – | KJ705320 |
| ***Alopoglossus copii*** | QCAZ8314 | – | – | – | KJ705318 |
| ***Alopoglossus festae**** | MZUTI4136 | – | KU999144 | – | KU999128 |
| ***Alopoglossus festae**** | MZUTI4134 | – | KU999145 | – | KU999129 |
| ***Alopoglossus festae**** | MZUTI2630 | – | KU999146 | – | KU999130 |
| ***Alopoglossus festae**** | MZUTI2994 | – | KU999147 | – | KU999133 |
| ***Alopoglossus festae**** | MZUTI3281 | – | KU999148 | – | KU999134 |
| ***Alopoglossus festae**** | MZUTI3370 | – | KU999149 | – | – |
| ***Alopoglossus festae**** | MZUTI3381 | – | KU999150 | – | – |
| ***Alopoglossus festae**** | MZUTI3393 | – | KU999151 | – | KU999135 |
| ***Alopoglossus festae**** | MZUTI3442 | – | KU999152 | – | – |
| ***Alopoglossus festae**** | MZUTI3463 | – | KU999153 | – | – |
| ***Alopoglossus festae**** | MZUTI3751 | – | KU999154 | – | KU999136 |
| ***Alopoglossus festae*** | QCAZ9158 | – | – | – | KJ705315 |
| ***Alopoglossus viridiceps**** | ANF1349 | – | KU999155 | – | KU999137 |
| ***Alopoglossus viridiceps**** | ANF1350 | – | KU999156 | – | KU999138 |
| ***Alopoglossus viridiceps**** | MZUTI3550 | – | KU999157 | – | KU999139 |
| ***Alopoglossus viridiceps**** | MZUTI3551 | – | KU999158 | – | KU999140 |
| ***Alopoglossus viridiceps**** | MZUTI3552 | – | KU999159 | – | KU999141 |
| ***Alopoglossus viridiceps*** | QCAZ10670 | – | – | – | KJ705316 |
| ***Cnemidophorus ocellifer*** | MRT946089 | – | AF420759 | – | AF420914 |
| ***Iphisa elegans*** | MRT977426 | – | AF420714 | – | AF420889 |
| ***Pholidobolus montium*** | QCAZ4051 | – | KC894360 | – | KC894374 |
| ***Ptychoglossus brevifrontalis*** | MHNSM | – | AY507884 | – | AY507895 |
| ***Atropoides mexicanus*** | USNM578906 | KC847268 | KC847255 | KC847271 | KC847289 |
| ***Bothriopsis bilineata*** | FHGO983 | – | – | AF292592 | AF292630 |
| ***Bothriopsis chloromelas*** | LSUMZ41037 | DQ305430 | DQ305453 | DQ305471 | DQ305488 |
| ***Bothriopsis pulchra*** | FHGO2142 | – | – | AF292593 | AF292631 |
| ***Bothriopsis taeniata*** | FHGO195 | AF057215 | AF057262 | AF292591 | AF292629 |
| ***Bothrocophias campbelli*** | INMHT | – | – | AF292584 | AF292622 |
| ***Bothrocophias hyoprora*** | FHGO4005 | – | – | AF292576 | AF292614 |
| ***Bothrocophias microphtalmus*** | FHGO2566 | – | – | AF292577 | AF292615 |
| ***Bothropoides alcatraz*** | CBGM002 | – | – | AY865821 | – |
| ***Bothropoides diporus*** | PT3404 | DQ305431 | DQ305454 | DQ305472 | DQ305489 |
| ***Bothropoides erythromelas*** | IB55541 | – | – | AF292588 | AF292626 |
| ***Bothropoides insularis*** | WWWg | AF057216 | AF057263 | AY223596 | AF188705 |
| ***Bothropoides jararaca*** | MM196 | EU867254 | EU867266 | EU867278 | EU867290 |
| ***Bothropoides lutzi*** | MTR14196 | – | – | KF801131 | KF801261 |
| ***Bothropoides marmoratus*** | CEPB8171 | – | – | KF801137 | KF801265 |
| ***Bothropoides matogrossensis*** | NORMAT113 | – | – | KF801149 | KF801277 |
| ***Bothropoides neuwiedi*** | IBSP74565 | – | – | KF801169 | KF801294 |
| ***Bothropoides pauloensis*** | CLP3 | EU867260 | EU867272 | EU867284 | EU867296 |
| ***Bothropoides pubescens*** | NOPA3860 | – | – | KF801227 | KF801344 |
| ***Bothrops asper*** | MZUCR11152 | AF057218 | GQ372868 | EU624301 | FJ985716 |
| ***Bothrops atrox*** | WWW743 | AY223659 | AY223672 | AY223598 | AY223641 |
| ***Bothrops brazili*** | USNM17831 | EU867252 | EU867264 | EU867276 | EU867288 |
| ***Bothrops caribbaeus*** | – | – | – | AF292598 | AF292636 |
| ***Bothrops jararacussu*** | DPL104 | AY223661 | AY223674 | AY223602 | AY223643 |
| ***Bothrops lanceolatus*** | NV | – | – | AF292599 | AF292637 |
| ***Bothrops leucurus*** | CLP195 | EU867255 | EU867267 | EU867279 | EU867291 |
| ***Bothrops lojanus*** | QCAZ6018 | – | FR691566 | FR691566 | FR691536 |
| ***Bothrops marajoensis*** | – | – | – | AF292605 | AF292643 |
| ***Bothrops moojeni*** | ITS418 | EU867257 | EU867269 | EU867281 | EU867293 |
| ***Bothrops osbornei*** | FHGO2166 | – | – | AF292595 | AF292633 |
| ***Bothrops osbornei**** | ANF2005 | KU999231 | KU999160 | KU999113 | KU999120 |
| ***Bothrops osbornei**** | ANF2107 | KU999232 | KU999161 | KU999114 | KU999121 |
| ***Bothrops osbornei**** | MZUTI3542 | KU999233 | KU999162 | KU999115 | KU999122 |
| ***Bothrops osbornei**** | MZUTI3865 | KU999234 | KU999163 | – | – |
| ***Bothrops pictus*** | – | – | – | AF292583 | AF292621 |
| ***Bothrops punctatus**** | AA002 | KU999235 | KU999164 | KU999116 | KU999123 |
| ***Bothrops punctatus**** | ANF1465 | KU999236 | KU999165 | KU999117 | KU999124 |
| ***Bothrops punctatus**** | ANF1575 | KU999237 | KU999166 | KU999118 | KU999125 |
| ***Bothrops punctatus**** | ANF1577 | KU999238 | KU999167 | KU999119 | KU999126 |
| ***Bothrops punctatus**** | ANF2101 | KU999239 | KU999168 | – | KU999127 |
| ***Bothrops punctatus*** | FHGO2452 | – | – | AF292594 | AF292632 |
| ***Lachesis acrochorda*** | CLP319 | JN870187 | JN870197 | JN870204 | JN870212 |
| ***Rhinocerophis alternatus*** | ITS358 | EU867251 | EU867263 | EU867275 | EU867287 |
| ***Rhinocerophis ammodytoides*** | MVZ223514 | AY223658 | AY223671 | – | AY223639 |
| ***Rhinocerophis cotiara*** | WWW | AF057217 | AF057264 | AY223597 | AY223640 |
| ***Rhinocerophis fonsecai*** | IB55543 | – | – | AF292580 | AF292618 |
| ***Rhinocerophis itapetiningae*** | ITS427 | EU867253 | EU867265 | EU867277 | EU867289 |
