# Supplementary material for: Comparative Phylogeography Reveals Cryptic Diversity and Repeated Patterns of Cladogenesis for Amphibians and Reptiles in Northwestern Ecuador
Source: PLoS One. 2016 Apr 27;11(4):e0151746. doi: 10.1371/journal.pone.0151746 (PMC4847877; doi:10.1371/journal.pone.0151746)
Supplement: S2 Table — All PCR protocols included an initial 3-min step at 94°C and a final extension of 10 min at 72°C. (DOCX) [file pone.0151746.s003.docx]

| **Locus** | **Primer name** | **Sequence (5’-3’)** | **Reference** | **PCR profile: denaturation/annealing/extension [number of corresponding cycles]** |
| --- | --- | --- | --- | --- |
| 12S Amphibians | 12S-t-Phe-frog-F | ATAGCRCTGAARAYGCTRAGATG | Wiens *et al.* (2005) | 93 °C (30 sec), 58 – 50 °C (-1 °C/cycle) (30 sec), 72 °C (1 min) [x9]; 93 °C (30 sec), 50 °C (30 sec), 72 °C (1 min) [x26] |
|  | 12S-t-Val-frog-R | TGTAAGCGARAGGCTTTKGTTAAGCT |  |  |
| 12S Amphibians and Reptiles | Snake_12S_F | AAACTGGGATTAGATACCCCACTAT | Kocher *et al.* (1989) | 94 °C (45 sec), 55 °C for amphibians, 52°C for *Bothrops* (1 min), 72 °C (1 min) [x30-32] |
|  | Snake_12S_R | GTRCGCTTACCWTGTTACGACT | Wiens *et al.* (1999) |  |
| 16S Amphibians | 16Sc-F | GTRGGCCTAAAAGCAGCCAC | Darst & Cannatella (2004) | 93 °C (30 sec), 67 – 58 °C (-1 °C/cycle) (30 sec), 72 °C (1 min) [x10]; 93 °C (30 sec), 58 °C (30 sec), 72 °C (1 min) [x18] |
|  | 16Sbr-H-R | CCGGTCTGAACTCAGATCACGT | Palumbi *et al.* (1991) |  |
| 16S Amphibians and Reptiles | Snake_16S_F | CGCCTGTTTAYCAAAAACAT | Palumbi *et al.* (1991) | 94 °C (45 sec), 56-58 °C for amphibians, 53 °C for *Bothrops* and 55 °C for *Alopoglossus* (45 sec), 72 °C (1 min) [x22-28] |
|  | Snake_16S_R | CCGGTCTGAACTCAGATCACGT |  |  |
| Cytb Reptiles | Snake_Cytb_F | GACCTGTGATMTGAAAACCAYCGTTGT | Burbrink *et al.* (2000) | 94 °C (1 min), 50 or 58 °C (1 min), 72 °C (2 min) [x22-35] |
|  | Snake_Cytb_R | CTTTGGTTTACAAGAACAATGCTTTA |  |  |
| ND4 Reptiles | Snake_ND4_F | CACCTATGACTACCAAAAGCTCATGTAGAAGC | Arévalo *et al.* (1994) | 94 °C (25 sec), 56 or 60 °C (1 min), 72 °C (2 min) [x25-30] |
|  | Snake_ND4_R | CATTACTTTTACTTGGATTTGCACC |  |  |
