# Supplementary material for: Comparative Phylogeography Reveals Cryptic Diversity and Repeated Patterns of Cladogenesis for Amphibians and Reptiles in Northwestern Ecuador
Source: PLoS One. 2016 Apr 27;11(4):e0151746. doi: 10.1371/journal.pone.0151746 (PMC4847877; doi:10.1371/journal.pone.0151746)
Supplement: S3 Table — (DOCX) [file pone.0151746.s004.docx]

| **Species** | **Voucher** | **Locality** | **Latitude** | **Longitude** | **Elev.** |
| --- | --- | --- | --- | --- | --- |
| *Alopoglossus festae* | MZUTI 2630 | Esmeraldas, Bilsa | 0.34910 | -79.70967 | 555 |
| *Alopoglossus festae* | MZUTI 2994 | Pichincha, Selva Virgen | 0.10673 | -78.18542 | 355 |
| *Alopoglossus festae* | MZUTI 3281 | Pichincha, Milpe | 0.03905 | -78.87054 | 998 |
| *Alopoglossus festae* | MZUTI 3370 | Azuay, Flor y Selva | -2.65706 | -79.53111 | 136 |
| *Alopoglossus festae* | MZUTI 3381 | El Oro, Buenaventura | -3.66598 | -79.73933 | 1042 |
| *Alopoglossus festae* | MZUTI 3393 | El Oro, Buenaventura | -3.64797 | -79.75507 | 947 |
| *Alopoglossus festae* | MZUTI 3442 | El Oro, California | -3.37146 | -79.73430 | 328 |
| *Alopoglossus festae* | MZUTI 3463 | El Oro, California | -3.37146 | -79.73430 | 328 |
| *Alopoglossus festae* | MZUTI 3751 | Pichincha, Milpe | 0.03076 | -78.86667 | 1162 |
| *Alopoglossus viridiceps* | MZUTI 3550 | Pichincha, Séptimo Paraíso | -0.02808 | -78.76667 | 1537 |
| *Alopoglossus viridiceps* | MZUTI 3551 | Pichincha, Séptimo Paraíso | -0.02808 | -78.76667 | 1537 |
| *Alopoglossus viridiceps* | MZUTI 3552 | Pichincha, Séptimo Paraíso | -0.02808 | -78.76667 | 1537 |
| *Bothrops osbornei* | MZUTI 3542 | Pichincha, Mashpi lodge | 0.16352 | -78.87274 | 1060 |
| *Bothrops osbornei* | MZUTI 3865 | Pichincha, Mashpi lodge | 0.16603 | -78.87862 | 905 |
| *Pristimantis crenunguis* | MZUTI 530 | Pichincha, Séptimo Paraíso | -0.02885 | -78.76599 | 1525 |
| *Pristimantis crenunguis* | MZUTI 531 | Pichincha, Séptimo Paraíso | -0.02885 | -78.76599 | 1525 |
| *Pristimantis crenunguis* | MZUTI 532 | Pichincha, Séptimo Paraíso | -0.02885 | -78.76599 | 1525 |
| *Pristimantis crenunguis* | MZUTI 1398 | Pichincha, Sachatamia | -0.02470 | -78.75909 | 1704 |
| *Pristimantis crenunguis* | MZUTI 1399 | Pichincha, Sachatamia | -0.02513 | -78.75896 | 1716 |
| *Pristimantis crenunguis* | MZUTI 2987 | Pichincha, Séptimo Paraíso | -0.02829 | -78.76596 | 1541 |
| *Pristimantis crenunguis* | MZUTI 3067 | Pichincha, Séptimo Paraíso | -0.02891 | -78.76586 | 1523 |
| *Pristimantis crenunguis* | MZUTI 3068 | Pichincha, Séptimo Paraíso | -0.02891 | -78.76586 | 1523 |
| *Pristimantis crenunguis* | MZUTI 3069 | Pichincha, Séptimo Paraíso | -0.02891 | -78.76586 | 1523 |
| *Pristimantis crenunguis* | MZUTI 3292 | Imbabura, Los Cedros | 0.31842 | -78.78373 | 1764 |
| *Pristimantis crenunguis* | MZUTI 3296 | Imbabura, Los Cedros | 0.31842 | -78.78373 | 1764 |
| *Pristimantis crenunguis* | MZUTI 3304 | Imbabura, Los Cedros | 0.31842 | -78.78373 | 1764 |
| *Pristimantis esmeraldas* | MZUTI 2251 | Esmeraldas, Bilsa | 0.34910 | -79.70967 | 555 |
| *Pristimantis esmeraldas* | MZUTI 2232 | Esmeraldas, Canandé | 0.52645 | -79.20937 | 360 |
| *Pristimantis esmeraldas* | MZUTI 3375 | Esmeraldas, Tundaloma | 1.18317 | -78.75245 | 74 |
| *Pristimantis esmeraldas* | MZUTI 3554 | Esmeraldas, Tundaloma | 1.18317 | -78.75245 | 74 |
| *Pristimantis esmeraldas* | MZUTI 3545 | Esmeraldas, Tundaloma | 1.18317 | -78.75245 | 74 |
| *Pristimantis esmeraldas* | MZUTI 3540 | Esmeraldas, Tundaloma | 1.18317 | -78.75245 | 74 |
| *Pristimantis esmeraldas* | MZUTI 3819 | Esmeraldas, Itapoa | 0.51306 | -79.13396 | 341 |
| *Pristimantis esmeraldas* | MECN 3311 | Esmeraldas, Canandé | 0.52645 | -79.20937 | 360 |
| *Pristimantis esmeraldas* | MZUTI 3199 | Pichincha, Silanche | 0.14577 | -79.14338 | 418 |
| *Pristimantis labiosus* | MZUTI 573 | Pichincha, San Francisco |  |  |  |
| *Pristimantis labiosus* | MZUTI 574 | Pichincha, Chontilla | 0.11187 | -78.90275 | 1191 |
| *Pristimantis labiosus* | MZUTI 577 | Pichincha, Chontilla | 0.11187 | -78.90275 | 1191 |
| *Pristimantis labiosus* | MZUTI 589 | Pichincha, Chontilla | 0.11187 | -78.90275 | 1191 |
| *Pristimantis labiosus* | MZUTI 594 | Pichincha, Chontilla | 0.11187 | -78.90275 | 1191 |
| *Pristimantis labiosus* | MZUTI 1759 | Pichincha, El Abrazo | -0.00916 | -78.81133 | 1086 |
| *Pristimantis labiosus* | MECN 9527 | Esmeraldas, Canandé | 0.52645 | -79.20937 | 360 |
| *Pristimantis labiosus* | MECN 9528 | Esmeraldas, Canandé | 0.52645 | -79.20937 | 360 |
| *Pristimantis labiosus* | MZUTI 3000 | Esmeraldas, Itapoa | 0.51306 | -79.13396 | 341 |
| *Pristimantis labiosus* | MZUTI 3018 | Esmeraldas, Itapoa | 0.51307 | -79.13400 | 321 |
| *Pristimantis labiosus* | MZUTI 3051 | Esmeraldas, Itapoa | 0.51307 | -79.13400 | 321 |
| *Pristimantis labiosus* | MZUTI 3078 | Pichincha, Milpe | 0.03125 | -78.86621 | 1156 |
| *Pristimantis labiosus* | MZUTI 3079 | Pichincha, Milpe | 0.03125 | -78.86621 | 1156 |
| *Pristimantis labiosus* | MZUTI 3080 | Pichincha, Milpe | 0.03125 | -78.86621 | 1156 |
| *Pristimantis laticlavius* | MZUTI 1728 | Imbabura, Los Cedros | 0.31125 | -78.78095 | 1417 |
| *Pristimantis luteolateralis* | MZUTI 2196 | Pichincha, El Abrazo | -0.00913 | -78.81321 | 1064 |
| *Pristimantis luteolateralis* | MZUTI 2115 | Pichincha, El Abrazo | -0.00913 | -78.81321 | 1064 |
| *Pristimantis luteolateralis* | MZUTI 3093 | Pichincha, Milpe | 0.03249 | -78.86576 | 1113 |
| *Pristimantis luteolateralis* | MZUTI 2988 | Pichincha, Séptimo Paraíso | -0.02829 | -78.76596 | 1541 |
| *Pristimantis luteolateralis* | MZUTI 2110 | Pichincha, El Abrazo | -0.00913 | -78.81321 | 1064 |
| *Pristimantis luteolateralis* | MZUTI 2989 | Pichincha, Séptimo Paraíso | -0.02829 | -78.76596 | 1541 |
| *Pristimantis luteolateralis* | MZUTI 1767 | Pichincha, El Abrazo | -0.00913 | -78.81321 | 1064 |
| *Pristimantis luteolateralis* | MZUTI 3183 | Pichincha, Séptimo Paraíso | -0.02829 | -78.76596 | 1541 |
| *Pristimantis luteolateralis* | MZUTI 3092 | Pichincha, Milpe | 0.03249 | -78.86576 | 1113 |
| *Pristimantis luteolateralis* | MZUTI 3521 | Pichincha, Mashpi lodge | 0.16603 | -78.87862 | 905 |
| *Pristimantis luteolateralis* | MZUTI 1734 | Imbabura, Los Cedros | 0.32489 | -78.78094 | 1621 |
| *Pristimantis luteolateralis* | MZUTI 327 | Pichincha, Yellow House | -0.04505 | -78.75938 | 1498 |
| *Pristimantis luteolateralis* | MZUTI 328 | Pichincha, Yellow House | -0.04505 | -78.75938 | 1498 |
| *Pristimantis luteolateralis* | MZUTI 329 | Pichincha, Yellow House | -0.04505 | -78.75938 | 1498 |
| *Pristimantis luteolateralis* | MZUTI 330 | Pichincha, Yellow House | -0.04505 | -78.75938 | 1498 |
| *Pristimantis luteolateralis* | MZUTI 528 | Pichincha, Cascadas de Mindo | -0.07837 | -78.76429 | 1438 |
| *Pristimantis luteolateralis* | MZUTI 529 | Pichincha, Cascadas de Mindo | -0.07899 | -78.76405 | 1409 |
| *Pristimantis luteolateralis* | MZUTI 654 | Pichincha, Chontilla | 0.11187 | -78.90275 | 1241 |
| *Pristimantis luteolateralis* | MZUTI 655 | Pichincha, Chontilla | 0.11187 | -78.90275 | 1241 |
| *Pristimantis luteolateralis* | MZUTI 656 | Pichincha, Chontilla | 0.11187 | -78.90275 | 1241 |
| *Pristimantis luteolateralis* | MZUTI 657 | Pichincha, Chontilla | 0.11187 | -78.90275 | 1241 |
| *Pristimantis luteolateralis* | MZUTI 658 | Pichincha, Chontilla | 0.11187 | -78.90275 | 1241 |
| *Pristimantis luteolateralis* | MZUTI 659 | Pichincha, Chontilla | 0.11187 | -78.90275 | 1241 |
| *Pristimantis luteolateralis* | MZUTI 660 | Pichincha, Chontilla | 0.11187 | -78.90275 | 1241 |
| *Pristimantis luteolateralis* | MZUTI 661 | Pichincha, Chontilla | 0.11187 | -78.90275 | 1241 |
| *Pristimantis luteolateralis* | MZUTI 662 | Pichincha, Chontilla | 0.11187 | -78.90275 | 1241 |
| *Pristimantis luteolateralis* | MZUTI 663 | Pichincha, Chontilla | 0.11187 | -78.90275 | 1241 |
| *Pristimantis luteolateralis* | MZUTI 703 | Pichincha, Sueños de Bambú | -0.06655 | -78.77158 | 1391 |
| *Pristimantis luteolateralis* | MZUTI 1404 | Pichincha, El Abrazo | -0.00914 | -78.81284 | 1074 |
| *Pristimantis luteolateralis* | MZUTI 1405 | Pichincha, El Abrazo | -0.00914 | -78.81284 | 1074 |
| *Pristimantis luteolateralis* | MZUTI 1406 | Pichincha, Yellow House | -0.04418 | -78.75520 | 1492 |
| *Pristimantis luteolateralis* | MZUTI 1742 | Imbabura, Los Cedros | 0.32489 | -78.78094 | 1621 |
| *Pristimantis luteolateralis* | MZUTI 2110 | Pichincha, El Abrazo | -0.00914 | -78.81284 | 1074 |
| *Pristimantis luteolateralis* | MZUTI 2115 | Pichincha, El Abrazo | -0.00914 | -78.81284 | 1074 |
| *Pristimantis luteolateralis* | MZUTI 2988 | Pichincha, Séptimo Paraíso | -0.02829 | -78.76596 | 1541 |
| *Pristimantis luteolateralis* | MZUTI 2989 | Pichincha, Séptimo Paraíso | -0.02829 | -78.76596 | 1541 |
| *Pristimantis luteolateralis* | MZUTI 2990 | Pichincha, Séptimo Paraíso | -0.02829 | -78.76596 | 1541 |
| *Pristimantis luteolateralis* | MZUTI 3092 | Pichincha, Milpe | 0.03249 | -78.86576 | 1113 |
| *Pristimantis luteolateralis* | MZUTI 3093 | Pichincha, Milpe | 0.03249 | -78.86576 | 1113 |
| *Pristimantis luteolateralis* | MZUTI 3182 | Pichincha, Séptimo Paraíso | -0.02886 | -78.76598 | 1522 |
| *Pristimantis mindo* | MZUTI 1381 | Pichincha, Sachatamia | -0.02470 | -78.75909 | 1704 |
| *Pristimantis mindo* | MZUTI1382 | Pichincha, Sachatamia | -0.02470 | -78.75909 | 1704 |
| *Pristimantis mindo* | MZUTI 1383 | Pichincha, Sachatamia | -0.02064 | -78.75928 | 1740 |
| *Pristimantis mindo* | MZUTI 1755 | Imbabura, Los Cedros | 0.31840 | -78.78370 | 1790 |
| *Pristimantis mindo* | MZUTI 1756 | Imbabura, Los Cedros | 0.32328 | -78.78111 | 1581 |
| *Pristimantis mindo* | MZUTI 2109 | Pichincha, Séptimo Paraíso | -0.02886 | -78.76598 | 1522 |
| *Pristimantis mindo* | MZUTI 2284 | Pichincha, Yellow House | -0.04462 | -78.75407 | 1511 |
| *Pristimantis parvillus* | MZUTI 483 | Pichincha, Las Gralarias | -0.00158 | -78.73858 | 1793 |
| *Pristimantis parvillus* | MZUTI 2121 | Pichincha, Tandayapa Lodge | 0.00249 | -78.68083 | 1730 |
| *Pristimantis subsigillatus* | MZUTI 1999 | Pichincha, Selva Virgen | 0.10712 | -79.18007 | 250 |
| *Pristimantis subsigillatus* | MZUTI 2228 | Esmeraldas, Canandé | 0.52615 | -79.21282 | 361 |
| *Pristimantis subsigillatus* | MZUTI 2243 | Esmeraldas, Bilsa | 0.34614 | -79.71299 | 533 |
| *Pristimantis subsigillatus* | MZUTI 2653 | Pichincha, Selva Virgen | 0.10547 | -79.18734 | 345 |
| *Pristimantis subsigillatus* | MZUTI 2995 | Pichincha, Selva Virgen | 0.10615 | -79.18586 | 364 |
| *Pristimantis subsigillatus* | MZUTI 2996 | Pichincha, Selva Virgen | 0.10615 | -79.18586 | 364 |
| *Pristimantis subsigillatus* | MZUTI 2997 | Pichincha, Selva Virgen | 0.10615 | -79.18586 | 364 |
| *Pristimantis subsigillatus* | MZUTI 3087 | Pichincha, Milpe | 0.03076 | -78.86667 | 1162 |
| *Pristimantis subsigillatus* | MZUTI 3088 | Pichincha, Milpe | 0.03076 | -78.86667 | 1162 |
| *Pristimantis subsigillatus* | MZUTI 3196 | Pichincha, Silanche | 0.14528 | -79.14147 | 413 |
| *Pristimantis subsigillatus* | MZUTI 3198 | Pichincha, Silanche | 0.14467 | -79.14318 | 391 |
| *Pristimantis subsigillatus* | MZUTI 3433 | El Oro, California | -3.36799 | -79.73551 | 225 |
| *Pristimantis walkeri* | MECN 2762 | Esmeraldas, Monte Saíno | 0.69833 | -80.02833 | 208 |
| *Pristimantis walkeri* | MECN 2763 | Esmeraldas, Monte Saíno | 0.69833 | -80.02833 | 208 |
| *Pristimantis walkeri* | MZUTI 1770 | Santo Domingo, Otongachi | -0.32145 | -78.95094 | 661 |
| *Pristimantis walkeri* | MZUTI 3781 | Esmeraldas, Mache Chindul | 0.51032 | -79.72552 | 175 |
| *Pristimantis walkeri* | MZUTI 3257 | Cañar, Huatacón | -2.49018 | -79.18223 | 1048 |
| *Pristimantis walkeri* | MZUTI 3247 | Cañar, Huatacón | -2.49018 | -79.18223 | 1048 |
| *Pristimantis walkeri* | MZUTI 3243 | Cañar, Huatacón | -2.49018 | -79.18223 | 1048 |
| *Pristimantis walkeri* | MZUTI 3246 | Cañar, Huatacón | -2.49018 | -79.18223 | 1048 |
| *Pristimantis walkeri* | MZUTI 3255 | Cañar, Huatacón | -2.49018 | -79.18223 | 1048 |
| *Pristimantis walkeri* | MZUTI 3782 | Esmeraldas, Mache Chindul | 0.51032 | -79.72552 | 175 |
| *Pristimantis walkeri* | MZUTI 2990 | Pichincha, Selva Virgen | 0.10615 | -79.18586 | 364 |
| *Pristimantis walkeri* | MZUTI 1768 | Santo Domingo, Otongachi | -0.32145 | -78.95094 | 661 |
| *Pristimantis walkeri* | MZUTI 1770 | Santo Domingo, Otongachi | -0.32145 | -78.95094 | 661 |
| *Pristimantis walkeri* | MZUTI 2993 | Pichincha, Selva Virgen | 0.10547 | -79.18734 | 345 |
| *Pristimantis walkeri* | MZUTI 3183 | Pichincha, Selva Virgen | 0.10547 | -79.18734 | 345 |
