# Supplementary material for: Comparative Phylogeography Reveals Cryptic Diversity and Repeated Patterns of Cladogenesis for Amphibians and Reptiles in Northwestern Ecuador
Source: PLoS One. 2016 Apr 27;11(4):e0151746. doi: 10.1371/journal.pone.0151746 (PMC4847877; doi:10.1371/journal.pone.0151746)
Supplement: S4 Table — In general, localities are given as transcribed from the literature, museum records, Tropical Herping photographic database or HerpNET. Coordinates represent georeferencing attempts from gazetteers under standard guidelines, though some variation from the exact collecting locality will inevitably be present. Similarly, elevations are taken from Google Earth, and may not exactly match the elevations as originally reported. (DOCX) [file pone.0151746.s005.docx]

| **Species** | **Province** | **Locality** | **Latitude** | **Longitude** | **Elev.** | **Source** |
| --- | --- | --- | --- | --- | --- | --- |
| *Alopoglossus festae* | Azuay | Chilcaplaya | -3.31396 | -79.57194 | 562 | Tropical Herping |
| *Alopoglossus festae* | Azuay | Flor y Selva | -2.65706 | -79.53111 | 136 | Tropical Herping |
| *Alopoglossus festae* | Azuay | Produmin | -3.08732 | -79.71493 | 359 | Torres-Carvajal and Lobos 2014 |
| *Alopoglossus festae* | Azuay | Sarayunga | -3.31431 | -79.58069 | 552 | Torres-Carvajal and Lobos 2014 |
| *Alopoglossus festae* | Bolívar | Balzapamba | -1.76670 | -79.18330 | 730 | Köhler et al. 2012 |
| *Alopoglossus festae* | Bolívar | Recinto San Francisco | -2.13427 | -79.07058 | 1377 | Torres-Carvajal and Lobos 2014 |
| *Alopoglossus festae* | Cañar | Huatacón | -2.49014 | -79.18221 | 1049 | Tropical Herping |
| *Alopoglossus festae* | Cañar | Manta Real | -2.55367 | -79.36425 | 257 | Almendariz and Carr 2007 |
| *Alopoglossus festae* | Carchi | Destacamento Militar | 1.18327 | -78.49224 | 244 | MECN 6720 |
| *Alopoglossus festae* | Carchi | Tobar Donoso | 1.18993 | -78.50413 | 223 | Tropical Herping |
| *Alopoglossus festae* | Chimborazo | Pallatanga | -1.99514 | -78.96544 | 1553 | Köhler et al. 2012 |
| *Alopoglossus festae* | Cotopaxi | Corazón–Moraspungo | -1.14643 | -79.15170 | 804 | Torres-Carvajal and Lobos 2014 |
| *Alopoglossus festae* | Cotopaxi | Finca de la familia Tapia | -0.38462 | -78.91377 | 985 | Torres-Carvajal and Lobos 2014 |
| *Alopoglossus festae* | Cotopaxi | La Maná | -0.93839 | -79.22536 | 208 | Torres-Carvajal and Lobos 2014 |
| *Alopoglossus festae* | Cotopaxi | Las Juntas | -0.39509 | -78.91325 | 1002 | Köhler et al. 2012 |
| *Alopoglossus festae* | Cotopaxi | Las Pampas | -0.35879 | -78.99651 | 1097 | Torres-Carvajal and Lobos 2014 |
| *Alopoglossus festae* | El Oro | 13 km W Piñas | -3.64089 | -79.77058 | 653 | Köhler et al. 2012 |
| *Alopoglossus festae* | El Oro | 7 km ESE Machala | -3.29111 | -79.90361 | 16 | Köhler et al. 2012 |
| *Alopoglossus festae* | El Oro | Bella María | -3.49392 | -79.89258 | 19 | Torres-Carvajal and Lobos 2014 |
| *Alopoglossus festae* | El Oro | Buenaventura | -3.64797 | -79.75507 | 947 | Tropical Herping |
| *Alopoglossus festae* | El Oro | California | -3.37146 | -79.73430 | 328 | Tropical Herping |
| *Alopoglossus festae* | El Oro | Piñas | -3.67833 | -79.68229 | 1059 | HerpNET |
| *Alopoglossus festae* | El Oro | Santa Rosa | -3.45098 | -79.95454 | 9 | HerpNET |
| *Alopoglossus festae* | Esmeraldas | 5 km from Playón | 1.05983 | -78.70742 | 96 | Torres-Carvajal and Lobos 2014 |
| *Alopoglossus festae* | Esmeraldas | 7 km W Durango | 1.07660 | -78.66096 | 109 | Torres-Carvajal and Lobos 2014 |
| *Alopoglossus festae* | Esmeraldas | Barro | 0.59508 | -79.96343 | 40 | Torres-Carvajal and Lobos 2014 |
| *Alopoglossus festae* | Esmeraldas | Bilsa | 0.35916 | -79.70055 | 463 | Tropical Herping |
| *Alopoglossus festae* | Esmeraldas | Caimito | 0.69546 | -80.08990 | 120 | Torres-Carvajal and Lobos 2014 |
| *Alopoglossus festae* | Esmeraldas | Cerro Ceibo | 0.66669 | -80.02198 | 55 | Tropical Herping |
| *Alopoglossus festae* | Esmeraldas | Cresta San Francisco | 0.69694 | -80.01861 | 75 | MECN 2871 |
| *Alopoglossus festae* | Esmeraldas | El Placer | 0.92071 | -78.67583 | 390 | Köhler et al. 2012 |
| *Alopoglossus festae* | Esmeraldas | Hacienda Equinox | -0.00721 | -79.39138 | 229 | Köhler et al. 2012 |
| *Alopoglossus festae* | Esmeraldas | La Tortuga | 0.60005 | -79.96806 | 43 | Torres-Carvajal and Lobos 2014 |
| *Alopoglossus festae* | Esmeraldas | Lote Escobar | 1.03669 | -78.60856 | 273 | Yánez-Muñoz 2005 |
| *Alopoglossus festae* | Esmeraldas | Lote Ventanas | 0.89815 | -78.61751 | 517 | Yánez-Muñoz 2005 |
| *Alopoglossus festae* | Esmeraldas | Monte Saíno | 0.69833 | -80.02833 | 208 | MECN 2872 |
| *Alopoglossus festae* | Esmeraldas | Muisne | 0.60000 | -80.01666 | 3 | MECN 1533 |
| *Alopoglossus festae* | Esmeraldas | Quinindé | 0.32753 | -79.47109 | 83 | HerpNET |
| *Alopoglossus festae* | Esmeraldas | Río Santiago | 0.41944 | -79.00333 | 469 | HerpNET |
| *Alopoglossus festae* | Esmeraldas | Río Tongora headwaters | 0.70425 | -80.07254 | 72 | Torres-Carvajal and Lobos 2014 |
| *Alopoglossus festae* | Esmeraldas | San Lorenzo | 1.28831 | -78.83691 | 11 | Köhler et al. 2012 |
| *Alopoglossus festae* | Esmeraldas | Tundaloma | 1.18236 | -78.75250 | 74 | Tropical Herping |
| *Alopoglossus festae* | Guayas | Cerro Blanco | -2.17465 | -80.02135 | 147 | Torres-Carvajal and Lobos 2014 |
| *Alopoglossus festae* | Guayas | Guayaquil | -2.04522 | -79.92553 | 46 | Köhler et al. 2012 |
| *Alopoglossus festae* | Guayas | Hacienda San Miguel | -2.17833 | -79.53141 | 18 | Köhler et al. 2012 |
| *Alopoglossus festae* | Guayas | Milagro | -2.12097 | -79.58221 | 10 | HerpNET |
| *Alopoglossus festae* | Guayas | Naranjal–Pasaje | -2.85937 | -79.70741 | 29 | Köhler et al. 2012 |
| *Alopoglossus festae* | Guayas | Nueva Unión Campesina | -2.72159 | -79.67007 | 18 | Torres-Carvajal and Lobos 2014 |
| *Alopoglossus festae* | Imbabura | Lita | 0.81527 | -78.38835 | 864 | HerpNET |
| *Alopoglossus festae* | Imbabura | Palma Real | 0.33333 | -78.93333 | 644 | HerpNET |
| *Alopoglossus festae* | Imbabura | Paramba | 0.81671 | -78.35002 | 698 | Köhler et al. 2012 |
| *Alopoglossus festae* | Imbabura | Río Mira | 0.83335 | -78.46675 | 1058 | HerpNET |
| *Alopoglossus festae* | Los Ríos | 3 km E Quevedo | -1.01401 | -79.44226 | 56 | HerpNET |
| *Alopoglossus festae* | Los Ríos | 4 km N Quevedo | -0.92776 | -79.49020 | 98 | Köhler et al. 2012 |
| *Alopoglossus festae* | Los Ríos | Buena Fé | -0.89306 | -79.48957 | 104 | HerpNET |
| *Alopoglossus festae* | Los Ríos | Finca Playa Grande | -0.98267 | -79.46961 | 82 | Köhler et al. 2012 |
| *Alopoglossus festae* | Los Ríos | Hacienda Cerro Chico | -0.64310 | -79.44306 | 146 | HerpNET |
| *Alopoglossus festae* | Los Ríos | Pichilangae | -1.06681 | -79.44765 | 44 | HerpNET |
| *Alopoglossus festae* | Los Ríos | Quevedo | -1.02852 | -79.46213 | 51 | Köhler et al. 2012 |
| *Alopoglossus festae* | Los Ríos | Río Baba | -0.33001 | -79.22008 | 423 | Köhler et al. 2012 |
| *Alopoglossus festae* | Los Ríos | Río Palenque | -0.58333 | -79.36667 | 173 | Miyata 1976 |
| *Alopoglossus festae* | Los Ríos | Vinces | -1.55379 | -79.75038 | 16 | Peracca 1904 |
| *Alopoglossus festae* | Manabí | Cerro Pata de Pájaro | 0.03333 | -79.98333 | 328 | Almendariz and Carr 2007 |
| *Alopoglossus festae* | Manabí | Jama Coaque | -0.11556 | -80.12472 | 299 | Torres-Carvajal and Lobos 2014 |
| *Alopoglossus festae* | Manabí | Lalo Loor | -0.08337 | -80.15004 | 75 | Tropical Herping |
| *Alopoglossus festae* | Manabí | Machalilla | -1.54147 | -80.69458 | 515 | MECN |
| *Alopoglossus festae* | Manabí | Near Pedernales | -0.08048 | -80.11890 | 335 | Tropical Herping |
| *Alopoglossus festae* | Manabí | Pacoche | -1.01206 | -80.83328 | 201 | Torres-Carvajal and Lobos 2014 |
| *Alopoglossus festae* | Manabí | Reserva Ayampe | -1.65417 | -80.81833 | 43 | Morales-Mite 2013 |
| *Alopoglossus festae* | Pichincha | El Abrazo del Árbol | -0.00929 | -78.81509 | 1089 | Tropical Herping |
| *Alopoglossus festae* | Pichincha | ENDESA | 0.09821 | -79.11701 | 470 | Torres-Carvajal and Lobos 2014 |
| *Alopoglossus festae* | Pichincha | Manduriacu | 0.28940 | -78.87093 | 1000 | Lynch et al. 2014 |
| *Alopoglossus festae* | Pichincha | Mashpi | 0.16299 | -78.87127 | 1045 | Tropical Herping |
| *Alopoglossus festae* | Pichincha | Milpe | 0.03905 | -78.87054 | 1055 | Tropical Herping |
| *Alopoglossus festae* | Pichincha | Puerto Quito | 0.11667 | -79.26661 | 143 | Tropical Herping |
| *Alopoglossus festae* | Pichincha | Río Blanco | -0.33088 | -78.87330 | 1055 | HerpNET |
| *Alopoglossus festae* | Pichincha | Río Caoni | 0.07555 | -79.05025 | 546 | Köhler et al. 2012 |
| *Alopoglossus festae* | Pichincha | Río Chirapi headwaters | 0.11623 | -78.91994 | 813 | Torres-Carvajal and Lobos 2014 |
| *Alopoglossus festae* | Pichincha | Selva Virgen | 0.10712 | -79.18007 | 355 | Tropical Herping |
| *Alopoglossus festae* | Santa Elena | Comuna Loma Alta | -1.83442 | -80.70291 | 72 | MECN |
| *Alopoglossus festae* | Santo Domingo | 24 S Santo Domingo | -0.42966 | -79.31905 | 275 | Köhler et al. 2012 |
| *Alopoglossus festae* | Santo Domingo | 37 km S Santo Domingo | -0.51157 | -79.36394 | 205 | HerpNET |
| *Alopoglossus festae* | Santo Domingo | Rancho Santa Teresita | -0.25277 | -79.37946 | 288 | Köhler et al. 2012 |
| *Alopoglossus festae* | Santo Domingo | Río Mulaule | -0.08722 | -79.14555 | 435 | HerpNET |
| *Alopoglossus festae* | Santo Domingo | Santo Domingo | -0.26291 | -79.15306 | 567 | Köhler et al. 2012 |
| *Alopoglossus viridiceps* | Esmeraldas | El Cristal | 0.85737 | -78.47911 | 1260 | Tropical Herping |
| *Alopoglossus viridiceps* | Imbabura | Charguayacu alto | 0.26871 | -78.67811 | 1292 | Köhler et al. 2012 |
| *Alopoglossus viridiceps* | Imbabura | Intag | 0.43478 | -78.67446 | 1552 | HerpNET |
| *Alopoglossus viridiceps* | Pichincha | Nanegal | 0.13142 | -78.67652 | 1165 | HerpNET |
| *Alopoglossus viridiceps* | Pichincha | Nanegalito | 0.06181 | -78.68215 | 1601 | HerpNET |
| *Alopoglossus viridiceps* | Pichincha | Santa Lucía | 0.11778 | -78.60755 | 1879 | Savit 2006 |
| *Alopoglossus viridiceps* | Pichincha | Saragoza–Río Cinto | -0.12891 | -78.75437 | 1522 | MECN 7369 |
| *Alopoglossus viridiceps* | Pichincha | Séptimo Paraíso | -0.02891 | -78.76586 | 1537 | Tropical Herping |
| *Alopoglossus viridiceps* | Pichincha | Yellow House | -0.04371 | -78.75351 | 1520 | Tropical Herping |
| *Bothrops osbornei* | Chimborazo | Sacramento | -1.98336 | -78.96024 | 1642 | Freire-Lascano 1991 |
| *Bothrops osbornei* | Cotopaxi | Las Pampas | -0.34836 | -79.07601 | 1248 | Schätti and Kramer 1991 |
| *Bothrops osbornei* | Pichincha | Downtown Mindo | -0.05313 | -78.77503 | 1252 | Tropical Herping |
| *Bothrops osbornei* | Pichincha | El Cinto | -0.06265 | -78.83489 | 1261 | Tropical Herping |
| *Bothrops osbornei* | Pichincha | El Monte Lodge | -0.06912 | -78.76195 | 1316 | Tropical Herping |
| *Bothrops osbornei* | Pichincha | Las Tangaras | -0.08089 | -78.76678 | 1383 | Tropical Herping |
| *Bothrops osbornei* | Pichincha | Las Tolas | 0.04626 | -78.78464 | 1605 | Yánez-Muñoz et al. 2009 |
| *Bothrops osbornei* | Pichincha | Lower Mashpi | 0.16154 | -78.88172 | 775 | Tropical Herping |
| *Bothrops osbornei* | Pichincha | Mashpi | 0.16352 | -78.87274 | 1060 | Tropical Herping |
| *Bothrops osbornei* | Pichincha | Mindo Garden | -0.07335 | -78.75423 | 1358 | Tropical Herping |
| *Bothrops osbornei* | Pichincha | Nanegal | 0.13142 | -78.67652 | 1165 | Tropical Herping |
| *Bothrops osbornei* | Pichincha | Pedro Vicente Maldonado | 0.05361 | -78.92109 | 938 | Tropical Herping |
| *Bothrops osbornei* | Pichincha | Río Blanco | -0.40189 | -78.79909 | 1657 | HerpNET |
| *Bothrops osbornei* | Pichincha | Séptimo Paraíso | -0.02891 | -78.76586 | 1541 | Tropical Herping |
| *Bothrops osbornei* | Pichincha | Tandapi | -0.41522 | -78.79728 | 1455 | Tropical Herping |
| *Bothrops osbornei* | Pichincha | Yellow House | -0.04234 | -78.75941 | 1489 | Tropical Herping |
| *Bothrops punctatus* | Carchi | Tobar Donoso | 1.18993 | -78.50413 | 223 | Yanez-Muñoz et al. 2009 |
| *Bothrops punctatus* | Esmeraldas | Alto Tambo | 0.90010 | -78.54533 | 806 | Tropical Herping |
| *Bothrops punctatus* | Esmeraldas | Canandé | 0.52993 | -79.03541 | 594 | Yánez-Muñoz et al. 2004 |
| *Bothrops punctatus* | Esmeraldas | Durango | 1.06340 | -78.59397 | 206 | Tropical Herping |
| *Bothrops punctatus* | Esmeraldas | Itapoa | 0.51307 | -79.13401 | 431 | Tropical Herping |
| *Bothrops punctatus* | Esmeraldas | Mataje | 1.35573 | -78.71782 | 56 | Tropical Herping |
| *Bothrops punctatus* | Esmeraldas | Otokiki | 0.88728 | -78.54119 | 856 | Tropical Herping |
| *Bothrops punctatus* | Esmeraldas | Playa de Oro | 1.26897 | -78.79641 | 46 | HerpNET |
| *Bothrops punctatus* | Esmeraldas | Río Onzole | 0.87433 | -79.05721 | 15 | Tropical Herping |
| *Bothrops punctatus* | Esmeraldas | Río Santiago | 0.41944 | -79.00333 | 469 | HerpNET |
| *Bothrops punctatus* | Esmeraldas | Tangareal | 0.77311 | -79.09499 | 71 | Morales 2004 |
| *Bothrops punctatus* | Esmeraldas | Tesoro Escondido | 0.54187 | -79.14495 | 267 | Tropical Herping |
| *Bothrops punctatus* | Imbabura | 5 km E Lita | 0.84918 | -78.42623 | 705 | Campbell and Lamar 1992 |
| *Bothrops punctatus* | Imbabura | Chuchubi | 0.84935 | -78.43014 | 789 | Tropical Herping |
| *Bothrops punctatus* | Imbabura | Lita | 0.81527 | -78.38835 | 864 | Tropical Herping |
| *Bothrops punctatus* | Pichincha | Selva Virgen | 0.10974 | -79.18545 | 341 | Tropical Herping |
| *Bothrops punctatus* | Santo Domingo | 18 km W Santo Domingo | -0.25268 | -79.37782 | 290 | Amaral 1923 |
| *Bothrops punctatus* | Santo Domingo | La Florida | -0.31928 | -78.98646 | 781 | Tropical Herping |
| *Pristimantis buenaventura* | El Oro | 12 km W Piñas | -3.62992 | -79.76097 | 1039 | Lynch and Duellman 1997 |
| *Pristimantis buenaventura* | El Oro | California | -3.36799 | -79.73551 | 225 | Tropical Herping |
| *Pristimantis buenaventura* | El Oro | Cascadas de Manuel | -3.21341 | -79.72598 | 806 | MECN 11339 |
| *Pristimantis buenaventura* | El Oro | Marcabelí | -3.73918 | -79.89429 | 662 | MECN 10888 |
| *Pristimantis buenaventura* | El Oro | Playa Limón | -3.50096 | -79.74701 | 816 | MECN 10778 |
| *Pristimantis buenaventura* | El Oro | Reserva Buenaventura | -3.65858 | -79.77078 | 534 | MECN 9472 |
| *Pristimantis buenaventura* | El Oro | Vicinities of Buenaventura | -3.66014 | -79.77928 | 565 | Tropical Herping |
| *Pristimantis buenaventura* | Loja | 33 km SSE Portovelo | -3.89563 | -79.54668 | 1070 | Lynch and Duellman 1997 |
| *Pristimantis crenunguis* | Imbabura | Los Cedros | 0.31842 | -78.78373 | 1764 | Tropical Herping |
| *Pristimantis crenunguis* | Pichincha | 12.6 km E La Palma | -0.36941 | -78.78738 | 1678 | Lynch and Duellman 1997 |
| *Pristimantis crenunguis* | Pichincha | 2.1 km E Tandapi | -0.37587 | -78.76828 | 1675 | Lynch and Duellman 1997 |
| *Pristimantis crenunguis* | Pichincha | 3.5 km NE Mindo | -0.03116 | -78.75617 | 1638 | Lynch and Duellman 1997 |
| *Pristimantis crenunguis* | Pichincha | La Palma | -0.31098 | -78.89450 | 1268 | Lynch and Duellman 1997 |
| *Pristimantis crenunguis* | Pichincha | Las Gralarias | -0.00158 | -78.73858 | 1793 | Tropical Herping |
| *Pristimantis crenunguis* | Pichincha | Las Tolas | 0.04626 | -78.78464 | 1605 | Yánez-Muñoz et al. 2009 |
| *Pristimantis crenunguis* | Pichincha | Maquipucuna | 0.11757 | -78.67447 | 1490 | Tropical Herping |
| *Pristimantis crenunguis* | Pichincha | Nanegal | 0.13142 | -78.67652 | 1165 | Lynch and Duellman 1997 |
| *Pristimantis crenunguis* | Pichincha | Nanegalito | 0.06181 | -78.68215 | 1601 | Lynch and Duellman 1997 |
| *Pristimantis crenunguis* | Pichincha | Pachijal | 0.01609 | -78.72641 | 1688 | Lynch and Duellman 1997 |
| *Pristimantis crenunguis* | Pichincha | Quebrada la Plata | -0.36943 | -78.77618 | 1598 | Lynch and Duellman 1997 |
| *Pristimantis crenunguis* | Pichincha | Road to Mindo | -0.03116 | -78.75617 | 1638 | Tropical Herping |
| *Pristimantis crenunguis* | Pichincha | Sachatamia | -0.02513 | -78.75896 | 1716 | Tropical Herping |
| *Pristimantis crenunguis* | Pichincha | Séptimo Paraíso | -0.02891 | -78.76586 | 1541 | Tropical Herping |
| *Pristimantis crenunguis* | Pichincha | Tamboquinde | 0.00933 | -78.67343 | 1646 | Yánez-Muñoz et al. 2009 |
| *Pristimantis crenunguis* | Pichincha | Tandapi | -0.41522 | -78.79728 | 1455 | Lynch and Duellman 1997 |
| *Pristimantis crenunguis* | Pichincha | Tandayapa | 0.00205 | -78.67880 | 1734 | Tropical Herping |
| *Pristimantis crenunguis* | Pichincha | Yellow House | -0.04234 | -78.75941 | 1489 | Lynch and Duellman 1997 |
| *Pristimantis crenunguis* | Santo Domingo | 16 km E La Palma | -0.36117 | -78.80241 | 1639 | Lynch and Duellman 1997 |
| *Pristimantis crenunguis* | Santo Domingo | Guajalito | -0.21341 | -78.85016 | 1768 | Reyes 2008 |
| *Pristimantis crenunguis* | Santo Domingo | Río Faisanes | -0.31667 | -78.87001 | 1549 | Lynch and Duellman 1997 |
| *Pristimantis labiosus* | Carchi | Río Baboso | 0.88333 | -78.44999 | 629 | Lynch and Duellman 1997 |
| *Pristimantis labiosus* | Cotopaxi | Guayacán-Pucayacu | -0.80236 | -79.16046 | 505 | Tropical Herping |
| *Pristimantis labiosus* | Cotopaxi | Hacienda La Mariela | -1.14854 | -79.10070 | 939 | MECN 1746 |
| *Pristimantis labiosus* | Cotopaxi | La Envidia | -1.00970 | -79.23769 | 425 | Tropical Herping |
| *Pristimantis labiosus* | Cotopaxi | La Maná | -0.93839 | -79.22536 | 208 | Tropical Herping |
| *Pristimantis labiosus* | Esmeraldas | Alto Tambo | 0.90010 | -78.54533 | 806 | Lynch and Duellman 1997 |
| *Pristimantis labiosus* | Esmeraldas | Canandé | 0.52993 | -79.03541 | 594 | Yánez-Muñoz et al. 2004 |
| *Pristimantis labiosus* | Esmeraldas | Charco Vicente | 0.69618 | -78.91089 | 79 | Morales 2004 |
| *Pristimantis labiosus* | Esmeraldas | Durango | 1.06340 | -78.59397 | 206 | Tropical Herping |
| *Pristimantis labiosus* | Esmeraldas | Golondrinas | 0.52301 | -78.89471 | 795 | MECN 3901 |
| *Pristimantis labiosus* | Esmeraldas | Gualpí | 0.78173 | -79.15993 | 63 | Morales 2004 |
| *Pristimantis labiosus* | Esmeraldas | Hacienda Equinox | -0.00721 | -79.39138 | 229 | Lynch and Duellman 1997 |
| *Pristimantis labiosus* | Esmeraldas | Itapoa | 0.51307 | -79.13401 | 431 | Tropical Herping |
| *Pristimantis labiosus* | Esmeraldas | La Concordia | -0.00027 | -79.41194 | 194 | Tropical Herping |
| *Pristimantis labiosus* | Esmeraldas | La Tabla | 0.84526 | -78.74467 | 130 | Morales 2004 |
| *Pristimantis labiosus* | Esmeraldas | Lote Quijano | 1.00058 | -78.62473 | 204 | Yánez-Muñoz 2005 |
| *Pristimantis labiosus* | Esmeraldas | Río Cupa | 0.38333 | -79.48334 | 102 | HerpNET |
| *Pristimantis labiosus* | Esmeraldas | Río Negro | 0.91667 | -78.58332 | 681 | MECN 3303 |
| *Pristimantis labiosus* | Esmeraldas | Salto del Bravo | 0.83182 | -78.75802 | 291 | Tropical Herping |
| *Pristimantis labiosus* | Esmeraldas | Tangareal | 0.77311 | -79.09499 | 71 | Morales 2004 |
| *Pristimantis labiosus* | Imbabura | Cotacachi Cayapas | 0.57073 | -78.68374 | 749 | Tropical Herping |
| *Pristimantis labiosus* | Imbabura | Lita | 0.81527 | -78.38835 | 864 | Lynch and Duellman 1997 |
| *Pristimantis labiosus* | Imbabura | Río Naranjal | 0.35155 | -78.91703 | 799 | MECN 3512 |
| *Pristimantis labiosus* | Los Ríos | Río Palenque | -0.58333 | -79.36667 | 173 | Lynch and Duellman 1997 |
| *Pristimantis labiosus* | Pichincha | 16 km NE La Palma | -0.18224 | -78.88003 | 989 | Lynch and Duellman 1997 |
| *Pristimantis labiosus* | Pichincha | 5 km E La Palma | -0.31184 | -78.90321 | 1106 | Lynch and Duellman 1997 |
| *Pristimantis labiosus* | Pichincha | Cordillera de Chontilla | 0.11187 | -78.90322 | 1161 | MZUTI 574 |
| *Pristimantis labiosus* | Pichincha | El Abrazo del Árbol | -0.00916 | -78.81133 | 1086 | Tropical Herping |
| *Pristimantis labiosus* | Pichincha | El Chalpi-Saguangal | 0.21503 | -78.83852 | 769 | Yánez-Muñoz et al. 2009 |
| *Pristimantis labiosus* | Pichincha | Fundación Durini | 0.27472 | -79.14623 | 306 | MECN 2606 |
| *Pristimantis labiosus* | Pichincha | La Palma | -0.31452 | -78.92664 | 926 | Lynch et al. 1976 |
| *Pristimantis labiosus* | Pichincha | Manduriacu | 0.28940 | -78.87093 | 1000 | Tropical Herping |
| *Pristimantis labiosus* | Pichincha | Mangaloma | 0.11080 | -78.98909 | 701 | Tropical Herping |
| *Pristimantis labiosus* | Pichincha | Mashpi | 0.16663 | -78.87695 | 950 | Tropical Herping |
| *Pristimantis labiosus* | Pichincha | Mashpi Río Amagusa | 0.15864 | -78.85378 | 1272 | Tropical Herping |
| *Pristimantis labiosus* | Pichincha | Río Pitsará | 0.24333 | -79.11333 | 380 | Lynch and Duellman 1997 |
| *Pristimantis labiosus* | Pichincha | Río Silanche | 0.08782 | -79.04908 | 391 | Tropical Herping |
| *Pristimantis labiosus* | Pichincha | Un Poco de Chocó | 0.05382 | -78.84310 | 1183 | Tropical Herping |
| *Pristimantis labiosus* | Santo Domingo | 11 km E Santo Domingo | -0.23209 | -79.03862 | 824 | Lynch and Duellman 1997 |
| *Pristimantis labiosus* | Santo Domingo | 5 km NW La Florida | -0.22521 | -79.05155 | 764 | Lynch and Duellman 1997 |
| *Pristimantis labiosus* | Santo Domingo | 6 km E Santo Domingo | -0.23826 | -79.09549 | 872 | Lynch and Duellman 1997 |
| *Pristimantis labiosus* | Santo Domingo | Centinela | -0.58334 | -79.35003 | 164 | Lynch and Duellman 1997 |
| *Pristimantis labiosus* | Santo Domingo | La Florida | -0.31928 | -78.98646 | 781 | Tropical Herping |
| *Pristimantis labiosus* | Santo Domingo | La Perla | 0.13417 | -79.49432 | 132 | Tropical Herping |
| *Pristimantis labiosus* | Santo Domingo | Otongachi | -0.32039 | -78.95176 | 846 | Tropical Herping |
| *Pristimantis labiosus* | Santo Domingo | Santo Domingo | -0.26291 | -79.15306 | 567 | Lynch and Duellman 1997 |
| *Pristimantis labiosus* | Santo Domingo | Tinalandia | -0.30315 | -79.05229 | 747 | Tropical Herping |
| *Pristimantis luteolateralis* | Cotopaxi | Las Pampas | -0.34836 | -79.07601 | 1248 | Tropical Herping |
| *Pristimantis luteolateralis* | Cotopaxi | Otonga | -0.42633 | -79.02406 | 1638 | Tropical Herping |
| *Pristimantis luteolateralis* | Imbabura | Los Cedros | 0.31842 | -78.78373 | 1764 | MZUTI 1742 |
| *Pristimantis luteolateralis* | Imbabura | Manduriacu | 0.31147 | -78.86240 | 1402 | Lynch et al. 2014 |
| *Pristimantis luteolateralis* | Pichincha | 3.5 km NE Mindo | -0.03143 | -78.79781 | 1268 | Lynch and Duellman 1997 |
| *Pristimantis luteolateralis* | Pichincha | Cascadas de Mindo | -0.07837 | -78.76429 | 1438 | Tropical Herping |
| *Pristimantis luteolateralis* | Pichincha | Cordillera de Chontilla | 0.11509 | -78.89180 | 1257 | Tropical Herping |
| *Pristimantis luteolateralis* | Pichincha | Curipogio | 0.13112 | -78.67632 | 1171 | MECN 1766 |
| *Pristimantis luteolateralis* | Pichincha | El Abrazo del Árbol | -0.00937 | -78.81378 | 1089 | Tropical Herping |
| *Pristimantis luteolateralis* | Pichincha | El Cedral | 0.11196 | -78.59164 | 1745 | Tropical Herping |
| *Pristimantis luteolateralis* | Pichincha | El Monte Lodge | -0.06912 | -78.76195 | 1316 | Tropical Herping |
| *Pristimantis luteolateralis* | Pichincha | Gavilán Orongo | 0.17186 | -78.66177 | 1259 | Tropical Herping |
| *Pristimantis luteolateralis* | Pichincha | Guambupe | 0.11167 | -78.90255 | 1164 | Tropical Herping |
| *Pristimantis luteolateralis* | Pichincha | La Hesperie | -0.35026 | -78.85091 | 1328 | Tropical Herping |
| *Pristimantis luteolateralis* | Pichincha | Las Tangaras | -0.08089 | -78.76678 | 1383 | Tropical Herping |
| *Pristimantis luteolateralis* | Pichincha | Las Tolas | 0.04626 | -78.78464 | 1605 | MECN 5810 |
| *Pristimantis luteolateralis* | Pichincha | Maquipucuna | 0.12040 | -78.68451 | 1401 | Tropical Herping |
| *Pristimantis luteolateralis* | Pichincha | Mashpi | 0.16603 | -78.87862 | 905 | Tropical Herping |
| *Pristimantis luteolateralis* | Pichincha | Milpe | 0.03249 | -78.86576 | 1113 | Tropical Herping |
| *Pristimantis luteolateralis* | Pichincha | Mindo Lago | -0.04834 | -78.77402 | 1270 | Tropical Herping |
| *Pristimantis luteolateralis* | Pichincha | Nanegal | 0.13142 | -78.67652 | 1165 | Tropical Herping |
| *Pristimantis luteolateralis* | Pichincha | Nuevo Mundo | 0.10006 | -78.86691 | 1342 | MECN 2011 |
| *Pristimantis luteolateralis* | Pichincha | Pachijal | 0.02771 | -78.78948 | 1277 | Tropical Herping |
| *Pristimantis luteolateralis* | Pichincha | Río Orito | -0.29458 | -78.88296 | 1405 | Tropical Herping |
| *Pristimantis luteolateralis* | Pichincha | Santa Lucía | 0.11778 | -78.60755 | 1879 | Tropical Herping |
| *Pristimantis luteolateralis* | Pichincha | Santa Rosa | -0.00234 | -78.72726 | 1755 | Tropical Herping |
| *Pristimantis luteolateralis* | Pichincha | Saragoza–Río Cinto | -0.12891 | -78.75437 | 1522 | Yánez-Muñoz et al. 2009 |
| *Pristimantis luteolateralis* | Pichincha | Sueños de Bambú | -0.06655 | -78.77158 | 1391 | Tropical Herping |
| *Pristimantis luteolateralis* | Pichincha | Tandapi | -0.41522 | -78.79728 | 1455 | Lynch and Duellman 1997 |
| *Pristimantis luteolateralis* | Pichincha | Tandayapa Lodge | 0.00249 | -78.68083 | 1730 | Tropical Herping |
| *Pristimantis luteolateralis* | Pichincha | Upper Río Sardinas | 0.19456 | -78.83548 | 1150 | Tropical Herping |
| *Pristimantis luteolateralis* | Pichincha | Yellow House | -0.04234 | -78.75941 | 1489 | Tropical Herping |
| *Pristimantis luteolateralis* | Pichincha | 4 km NE Dos Ríos | -0.30188 | -78.88698 | 1415 | Lynch and Duellman 1997 |
| *Pristimantis luteolateralis* | Santo Domingo | 10 km E La Palma | -0.33508 | -78.83537 | 1543 | Lynch and Duellman 1997 |
| *Pristimantis luteolateralis* | Santo Domingo | 14 km W Chiriboga | -0.24352 | -78.84991 | 1731 | Lynch and Duellman 1997 |
| *Pristimantis luteolateralis* | Santo Domingo | 16 km E La Palma | -0.36117 | -78.80241 | 1639 | Lynch and Duellman 1997 |
| *Pristimantis luteolateralis* | Santo Domingo | Guajalito | -0.22875 | -78.82248 | 1790 | Tropical Herping |
| *Pristimantis luteolateralis* | Santo Domingo | Río Faisanes | -0.31667 | -78.87001 | 1549 | Lynch and Duellman 1997 |
| *Pristimantis luteolateralis* | Santo Domingo | Toachi-Chiriboga | -0.30355 | -78.86922 | 1444 | Tropical Herping |
| *Pristimantis mindo* | Imbabura | Junín | 0.27009 | -78.64975 | 1688 | Arteaga et al. 2013 |
| *Pristimantis mindo* | Imbabura | Los Cedros | 0.31842 | -78.78373 | 1764 | Arteaga et al. 2013 |
| *Pristimantis mindo* | Pichincha | Cascadas de Mindo | -0.08002 | -78.76251 | 1381 | Tropical Herping |
| *Pristimantis mindo* | Pichincha | Curipogio | 0.13112 | -78.67632 | 1171 | Tropical Herping |
| *Pristimantis mindo* | Pichincha | La Favorita | -0.22833 | -78.76503 | 1810 | Tropical Herping |
| *Pristimantis mindo* | Pichincha | Las Tolas | 0.04626 | -78.78464 | 1605 | Yánez-Muñoz et al. 2009 |
| *Pristimantis mindo* | Pichincha | Mashpi | 0.16537 | -78.87244 | 1060 | Tropical Herping |
| *Pristimantis mindo* | Pichincha | Milpe | 0.03905 | -78.87054 | 1055 | Tropical Herping |
| *Pristimantis mindo* | Pichincha | Sachatamia Lodge | -0.02064 | -78.75928 | 1740 | Tropical Herping |
| *Pristimantis mindo* | Pichincha | Saragoza–Río Cinto | -0.12891 | -78.75437 | 1522 | Tropical Herping |
| *Pristimantis mindo* | Pichincha | Séptimo Paraíso | -0.02794 | -78.76640 | 1530 | Tropical Herping |
| *Pristimantis mindo* | Pichincha | Yellow House | -0.04323 | -78.75259 | 1562 | Tropical Herping |
| *Pristimantis nietoi* | Esmeraldas | 4 km E Durango | 1.06699 | -78.56060 | 388 | Lynch and Duellman 1997 |
| *Pristimantis nietoi* | Esmeraldas | Canandé | 0.52227 | -79.21309 | 363 | Tropical Herping |
| *Pristimantis nietoi* | Esmeraldas | Charco Vicente | 0.69618 | -78.91089 | 79 | Morales 2004 |
| *Pristimantis nietoi* | Esmeraldas | Gualpí | 0.78173 | -79.15993 | 63 | Morales 2004 |
| *Pristimantis nietoi* | Esmeraldas | La Tabla | 0.84526 | -78.74467 | 130 | Morales 2004 |
| *Pristimantis nietoi* | Esmeraldas | Lote Ventanas | 0.89815 | -78.61751 | 517 | Yánez-Muñoz 2005 |
| *Pristimantis nietoi* | Esmeraldas | San Miguel de Cayapas | 1.05553 | -78.62370 | 142 | Lynch and Duellman 1997 |
| *Pristimantis nietoi* | Esmeraldas | Tangareal | 0.77311 | -79.09499 | 71 | Morales 2004 |
| *Pristimantis nietoi* | Pichincha | Itapoa Reserve | 0.51307 | -79.13401 | 321 | Tropical Herping |
| *Pristimantis subsigillatus* | Bolívar | Balzapamba | -1.76670 | -79.18330 | 730 | Lynch and Duellman 1997 |
| *Pristimantis subsigillatus* | Cañar | Manta Real | -2.55367 | -79.36425 | 257 | Lynch and Duellman 1997 |
| *Pristimantis subsigillatus* | El Oro | Buenaventura | -3.64797 | -79.75507 | 947 | Yánez-Muñoz et al. 2009 |
| *Pristimantis subsigillatus* | El Oro | California | -3.36799 | -79.73551 | 225 | Tropical Herping |
| *Pristimantis subsigillatus* | Esmeraldas | Alto Tambo | 0.86672 | -78.53334 | 1056 | Lynch and Duellman 1997 |
| *Pristimantis subsigillatus* | Esmeraldas | Bilsa | 0.35916 | -79.70055 | 463 | Lynch and Duellman 1997 |
| *Pristimantis subsigillatus* | Esmeraldas | Caimito | 0.69546 | -80.08990 | 120 | Tropical Herping |
| *Pristimantis subsigillatus* | Esmeraldas | Canandé | 0.52615 | -79.21282 | 361 | Tropical Herping |
| *Pristimantis subsigillatus* | Esmeraldas | Cresta San Francisco | 0.69694 | -80.01861 | 75 | MECN 2780 |
| *Pristimantis subsigillatus* | Esmeraldas | Hacienda Equinox | -0.00721 | -79.39138 | 229 | Lynch and Duellman 1997 |
| *Pristimantis subsigillatus* | Esmeraldas | La Boca | 0.53697 | -79.44499 | 39 | Lynch and Duellman 1997 |
| *Pristimantis subsigillatus* | Esmeraldas | La Tabla | 0.84526 | -78.74467 | 130 | Morales 2004 |
| *Pristimantis subsigillatus* | Esmeraldas | Laguna de Cube | 0.38333 | -79.67498 | 317 | Tropical Herping |
| *Pristimantis subsigillatus* | Esmeraldas | Lote Escobar | 1.03669 | -78.60856 | 273 | Yánez-Muñoz 2005 |
| *Pristimantis subsigillatus* | Esmeraldas | Lote Quijano | 1.00058 | -78.62473 | 204 | Yánez-Muñoz 2005 |
| *Pristimantis subsigillatus* | Esmeraldas | Punta Galeras | 0.80623 | -80.04810 | 27 | Ortega-Andrade and Altamirano 2004 |
| *Pristimantis subsigillatus* | Esmeraldas | Río San Francisco | 0.69944 | -80.01805 | 41 | MECN 2778 |
| *Pristimantis subsigillatus* | Esmeraldas | Salidero | 0.91333 | -78.56893 | 706 | Lynch and Duellman 1997 |
| *Pristimantis subsigillatus* | Esmeraldas | Tangareal | 0.77311 | -79.09499 | 71 | Morales 2004 |
| *Pristimantis subsigillatus* | Esmeraldas | Tundaloma | 1.18166 | -78.74945 | 74 | Tropical Herping |
| *Pristimantis subsigillatus* | Los Ríos | Río Baba | -0.33001 | -79.22008 | 423 | Lynch and Duellman 1997 |
| *Pristimantis subsigillatus* | Los Ríos | Río Palenque | -0.58333 | -79.36667 | 173 | Lynch and Duellman 1997 |
| *Pristimantis subsigillatus* | Manabí | Cerro Pata de Pájaro | 0.03333 | -79.98333 | 328 | Almendariz and Carr 2007 |
| *Pristimantis subsigillatus* | Manabí | Jama Coaque | -0.11556 | -80.12472 | 299 | Tropical Herping |
| *Pristimantis subsigillatus* | Manabí | Lalo Loor | -0.08337 | -80.15004 | 75 | Tropical Herping |
| *Pristimantis subsigillatus* | Manabí | Machalilla | -1.54147 | -80.69458 | 515 | Lynch and Duellman 1997 |
| *Pristimantis subsigillatus* | Manabí | Reserva Ayampe | -1.65417 | -80.81833 | 43 | Morales-Mite 2013 |
| *Pristimantis subsigillatus* | Manabí | Three Forests Trail | -0.09973 | -80.12656 | 398 | Tropical Herping |
| *Pristimantis subsigillatus* | Pichincha | El Abrazo del Árbol | -0.00918 | -78.81316 | 1092 | Tropical Herping |
| *Pristimantis subsigillatus* | Pichincha | El Chalpi-Saguangal | 0.21503 | -78.83852 | 769 | Yánez-Muñoz et al. 2009 |
| *Pristimantis subsigillatus* | Pichincha | El Paraíso | 0.07370 | -79.03573 | 580 | Tropical Herping |
| *Pristimantis subsigillatus* | Pichincha | Mashpi | 0.16403 | -78.87073 | 1060 | Tropical Herping |
| *Pristimantis subsigillatus* | Pichincha | Milpe | 0.03489 | -78.86713 | 1070 | Tropical Herping |
| *Pristimantis subsigillatus* | Pichincha | Río Sardinas | 0.19527 | -78.85194 | 803 | Tropical Herping |
| *Pristimantis subsigillatus* | Pichincha | Río Silanche | 0.14528 | -79.14147 | 413 | Tropical Herping |
| *Pristimantis subsigillatus* | Pichincha | Selva Virgen | 0.10547 | -79.18734 | 345 | Tropical Herping |
| *Pristimantis subsigillatus* | Pichincha | Road to Los Bancos | -0.00811 | -78.95688 | 902 | Tropical Herping |
| *Pristimantis subsigillatus* | Santa Elena | Comuna Loma Alta | -1.83442 | -80.70291 | 72 | Tropical Herping |
| *Pristimantis subsigillatus* | Santo Domingo | Centinela | -0.58334 | -79.35003 | 164 | Lynch and Duellman 1997 |
| *Pristimantis subsigillatus* | Santo Domingo | Hacienda Espinosa | -0.24111 | -79.25886 | 420 | Lynch and Duellman 1997 |
| *Pristimantis subsigillatus* | Santo Domingo | La Florida | -0.31928 | -78.98646 | 781 | Lynch and Duellman 1997 |
| *Pristimantis subsigillatus* | Santo Domingo | La Perla | 0.13417 | -79.49432 | 132 | Tropical Herping |
| *Pristimantis subsigillatus* | Santo Domingo | La Unión del Toachi | -0.32133 | -78.95467 | 893 | Tropical Herping |
| *Pristimantis subsigillatus* | Santo Domingo | Santo Domingo | -0.26291 | -79.15306 | 567 | Lynch and Duellman 1997 |
| *Pristimantis subsigillatus* | Santo Domingo | Tinalandia | -0.30316 | -79.05202 | 752 | Tropical Herping |
| *Pristimantis walkeri* | Azuay | 11 km W Luz María | -2.66459 | -79.51789 | 347 | Lynch and Duellman 1997 |
| *Pristimantis walkeri* | Bolívar | 11 km E Moraspungo | -1.15558 | -79.09177 | 961 | Lynch and Duellman 1997 |
| *Pristimantis walkeri* | Bolívar | 6 ESE Balsapamba | -1.76546 | -79.17263 | 845 | Lynch and Duellman 1997 |
| *Pristimantis walkeri* | Bolívar | Balzapamba | -1.76670 | -79.18330 | 730 | Lynch and Duellman 1997 |
| *Pristimantis walkeri* | Bolívar | Above Cumandá | -2.18562 | -79.11304 | 487 | Tropical Herping |
| *Pristimantis walkeri* | Cañar | Huatacón | -2.49018 | -79.18223 | 1048 | Tropical Herping |
| *Pristimantis walkeri* | Cañar | Manta Real | -2.55367 | -79.36425 | 257 | Lynch and Duellman 1997 |
| *Pristimantis walkeri* | Cotopaxi | 20 km W Pilaló | -0.88436 | -79.15534 | 438 | Lynch and Duellman 1997 |
| *Pristimantis walkeri* | Cotopaxi | La Envidia | -1.00970 | -79.23769 | 425 | Tropical Herping |
| *Pristimantis walkeri* | Cotopaxi | Las Juntas | -0.39509 | -78.91325 | 1002 | Lynch and Duellman 1997 |
| *Pristimantis walkeri* | Cotopaxi | Guayacán-Pucayacu | -0.70273 | -79.05681 | 972 | Tropical Herping |
| *Pristimantis walkeri* | Esmeraldas | Bilsa | 0.35916 | -79.70055 | 463 | Tropical Herping |
| *Pristimantis walkeri* | Esmeraldas | Cabo San Francisco | 0.65484 | -80.08175 | 78 | Tropical Herping |
| *Pristimantis walkeri* | Esmeraldas | Charco Vicente | 0.69618 | -78.91089 | 79 | Morales 2004 |
| *Pristimantis walkeri* | Esmeraldas | Cresta San Francisco | 0.69694 | -80.01861 | 75 | MECN 2765 |
| *Pristimantis walkeri* | Esmeraldas | Hacienda Equinox | -0.00721 | -79.39138 | 229 | Lynch and Duellman 1997 |
| *Pristimantis walkeri* | Esmeraldas | Laguna de Cube | 0.38333 | -79.67498 | 317 | Tropical Herping |
| *Pristimantis walkeri* | Esmeraldas | Monte Saíno | 0.69833 | -80.02833 | 208 | MECN 2761 |
| *Pristimantis walkeri* | Esmeraldas | Punta Galeras | 0.80623 | -80.04810 | 27 | Ortega-Andrade and Altamirano 2004 |
| *Pristimantis walkeri* | Imbabura | Río Aguas Verdes | 0.33101 | -78.93152 | 680 | Tropical Herping |
| *Pristimantis walkeri* | Los Ríos | Río Baba | -0.33157 | -79.22009 | 421 | Lynch and Duellman 1997 |
| *Pristimantis walkeri* | Los Ríos | Río Palenque | -0.58333 | -79.36667 | 173 | Lynch and Duellman 1997 |
| *Pristimantis walkeri* | Manabí | 12 N Puerto Cayo | -1.25934 | -80.74006 | 53 | Tropical Herping |
| *Pristimantis walkeri* | Manabí | Cerro Pata de Pájaro | 0.03333 | -79.98333 | 328 | Tropical Herping |
| *Pristimantis walkeri* | Manabí | Jama Coaque | -0.11556 | -80.12472 | 299 | Tropical Herping |
| *Pristimantis walkeri* | Manabí | Machalilla | -1.54147 | -80.69458 | 515 | Almendariz and Carr 2007 |
| *Pristimantis walkeri* | Manabí | Reserva Ayampe | -1.65417 | -80.81833 | 43 | MECN 10118 |
| *Pristimantis walkeri* | Manabí | Río Ayampe | -1.66945 | -80.74711 | 66 | Lynch and Duellman 1997 |
| *Pristimantis walkeri* | Manabí | San Sebastián | -1.60002 | -80.69974 | 602 | MECN 6054 |
| *Pristimantis walkeri* | Manabí | Near Jama Coaque | -0.11929 | -80.12123 | 410 | Tropical Herping |
| *Pristimantis walkeri* | Pichincha | 4 km NE Dos Ríos | -0.30748 | -78.89865 | 1155 | Lynch and Duellman 1997 |
| *Pristimantis walkeri* | Pichincha | OCP Puerto Quito | 0.08137 | -78.96651 | 795 | Valencia and Garzón 2013 |
| *Pristimantis walkeri* | Pichincha | La Palma | -0.31452 | -78.92664 | 926 | Lynch and Duellman 1997 |
| *Pristimantis walkeri* | Pichincha | Mashpi Shungo | 0.15889 | -78.90222 | 645 | Tropical Herping |
| *Pristimantis walkeri* | Pichincha | Pedro Vicente Maldonado | 0.05361 | -78.92109 | 938 | Lynch and Duellman 1997 |
| *Pristimantis walkeri* | Pichincha | San Juan de Puerto Quito | 0.06939 | -78.95823 | 143 | MECN 8604 |
| *Pristimantis walkeri* | Pichincha | Río Caoni | 0.07555 | -79.05025 | 546 | Lynch and Duellman 1997 |
| *Pristimantis walkeri* | Pichincha | Río Silanche | 0.14467 | -79.14318 | 391 | Tropical Herping |
| *Pristimantis walkeri* | Pichincha | Río Toachi | -0.31383 | -78.95531 | 887 | Lynch and Duellman 1997 |
| *Pristimantis walkeri* | Pichincha | Selva Virgen | 0.10503 | -79.18705 | 348 | Tropical Herping |
| *Pristimantis walkeri* | Santa Elena | Comuna Loma Alta | -1.83442 | -80.70291 | 72 | MECN 3163 |
| *Pristimantis walkeri* | Santo Domingo | 10 km E Patricia Pilar | -0.55205 | -79.30915 | 367 | Lynch and Duellman 1997 |
| *Pristimantis walkeri* | Santo Domingo | 18 km W Santo Domingo | -0.25268 | -79.37782 | 290 | Lynch and Duellman 1997 |
| *Pristimantis walkeri* | Santo Domingo | 5 km NW La Florida | -0.22521 | -79.05155 | 764 | Lynch and Duellman 1997 |
| *Pristimantis walkeri* | Santo Domingo | 5 km W La Florida | -0.26298 | -79.06803 | 769 | Lynch and Duellman 1997 |
| *Pristimantis walkeri* | Santo Domingo | 6 km E Santo Domingo | -0.23826 | -79.09549 | 872 | Lynch and Duellman 1997 |
| *Pristimantis walkeri* | Santo Domingo | 8 km SE Santo Domingo | -0.29415 | -79.09692 | 951 | Lynch and Duellman 1997 |
| *Pristimantis walkeri* | Santo Domingo | 9 km W Santo Domingo | -0.24032 | -79.29053 | 382 | Lynch and Duellman 1997 |
| *Pristimantis walkeri* | Santo Domingo | Centinela | -0.58334 | -79.35003 | 164 | Lynch and Duellman 1997 |
| *Pristimantis walkeri* | Santo Domingo | La Unión del Toachi | -0.32133 | -78.95467 | 893 | Tropical Herping |
| *Pristimantis walkeri* | Santo Domingo | Otongachi | -0.32145 | -78.95094 | 661 | Tropical Herping |
| *Pristimantis walkeri* | Santo Domingo | Tinalandia | -0.30488 | -79.05061 | 765 | Tropical Herping |
